# Microglial CD2AP deficiency exerts protection in an Alzheimer’s disease model of amyloidosis

**DOI:** 10.1186/s13024-024-00789-7

**Published:** 2024-12-18

**Authors:** Lingliang Zhang, Lingling Huang, Yuhang Zhou, Jian Meng, Liang Zhang, Yunqiang Zhou, Naizhen Zheng, Tiantian Guo, Shanshan Zhao, Zijie Wang, Yuanhui Huo, Yingjun Zhao, Xiao-fen Chen, Honghua Zheng, David M. Holtzman, Yun-wu Zhang

**Affiliations:** 1 Xiamen Key Laboratory of Brain Center, The First Affiliated Hospital of Xiamen University, and Fujian Provincial Key Laboratory of Neurodegenerative Disease and Aging Research, Institute of Neuroscience, School of Medicine, Xiamen University, Xiamen, Fujian 361102 China; 2 Department of Neurology, Hope Center for Neurological Disorders, Knight Alzheimer’s Disease Research Center, Washington University School of Medicine, St. Louis, MO USA

**Keywords:** β-amyloid, Alzheimer’s disease, C1q, CD2AP, CSF1R, Disease-associated microglia, Microglia

## Abstract

**Background:**

The CD2-associated protein (CD2AP) was initially identified in peripheral immune cells and regulates cytoskeleton and protein trafficking. Single nucleotide polymorphisms (SNPs) in the *CD2AP* gene have been associated with Alzheimer’s disease (AD). However, the functional role of CD2AP, especially its role in microglia during AD onset, remains elusive.

**Methods:**

CD2AP protein levels in cultured primary cells and in 5xFAD mice was studied. Microglial CD2AP-deficient mice were crossed with 5xFAD mice and the offspring were subjected to neuropathological assessment, behavioral tests, electrophysiology, RNA-seq, Golgi staining, and biochemistry analysis. Primary microglia were also isolated for assessing their uptake and morphology changes.

**Results:**

We find that CD2AP is abundantly expressed in microglia and its levels are elevated in the brain of AD patients and the 5xFAD model mice at pathological stages. We demonstrate that CD2AP haploinsufficiency in microglia significantly attenuates cognitive and synaptic deficits, weakens the response of microglia to Aβ and the formation of disease-associated microglia (DAM), and alleviates synapse loss in 5xFAD mice. We show that CD2AP-deficient microglia exhibit compromised uptake ability. In addition, we find that CD2AP expression is positively correlated with the expression of the complement C1q that is important for synapse phagocytosis and the formation of DAM in response to Aβ deposition. Moreover, we reveal that CD2AP interacts with colony stimulating factor 1 receptor (CSF1R) and regulates CSF1R cell surface levels, which may further affect C1q expression.

**Conclusions:**

Our results demonstrate that CD2AP regulates microgliosis and identify a protective function of microglial CD2AP deficiency against Aβ deposition, suggesting the importance of detailed investigation of AD-associated genes in different brain cells for thoroughly understanding their exact contribution to AD.

**Supplementary Information:**

The online version contains supplementary material available at 10.1186/s13024-024-00789-7.

## Background

Alzheimer’s disease (AD) is the most common form of dementia. In addition to the deposition of extracellular β-amyloid (Aβ) plaques and intracellular neuronal tau tangles as its two classic pathological hallmarks, AD is accompanied by synapse loss and neuroinflammation [1–4]. Altered Aβ homeostasis plays a crucial role in AD pathogenesis: mutations in genes involved in Aβ generation, including *APP*, *PSEN1*, and *PSEN2* are known to cause early-onset, familial AD (EOAD) [5, 6]; and as the most important risk factor for late-onset, sporadic AD (LOAD), *APOE* ε4-encoded ApoE4 accelerates early seeding of Aβ pathology and compromises Aβ metabolism and clearance through multiple pathways [7–9]. Recent genome-wide association studies (GWAS) have also identified single nucleotide polymorphisms (SNPs) in multiple other genes to be associated with LOAD [10, 11]. Interestingly, many of these genes, such as *TREM2*, *CR1*, *CD33*, etc., are highly expressed in microglia, indicating the importance of microglial dysfunction during AD onset [12, 13]. Microglia are innate immune cells residing in the brain and play important roles not only in neuroinflammation and phagocytosis of harmful substances, but also in synapse pruning during brain development and homeostasis maintenance of functional neuronal connections [14–16]. Microgliosis and disease-associated microglia (DAM) have been found in the brain of AD patients and animal models [17–19]. Excessive elimination of synapses in AD animal models, presumably mediated by a dysregulated complement system, has also been proposed [20–22]. Therefore, a complete elucidation on how AD-associated genes regulate microglial functions shall strengthen our understanding on the molecular mechanism underlying AD pathogenesis and help identify new targets for AD therapeutics.

CD2-associated protein (CD2AP), an adaptor protein, was named for its interaction with CD2, a transmembrane protein in T cells [23]. CD2AP contains many actin-binding and membrane protein-binding sites and thus can regulate the cytoskeleton and stabilize membrane proteins [23–26]. CD2AP is also involved in receptor endocytosis and vesicle transport [27, 28]. During recent GWAS and whole-genome sequencing analyses, SNPs around the *CD2AP* gene have been found to associate with AD [29–32]. However, so far functional studies on exploring the role of CD2AP in AD are limited. In a *Drosophila* model of AD, the fly ortholog of *CD2AP*, *cindr*, was implicated as a modulator of tau-mediated neurotoxicity; and loss of *cindr* promoted tau-induced neuronal loss and reduced synaptic strength and life span in flies [33, 34]. Downregulation of CD2AP in cell cultures could trap APP in early endosomes to limit its degradation and thereby promote Aβ generation, whereas overexpression of CD2AP showed the opposite effects [35, 36]. CD2AP-deficient mice exhibited compromised blood–brain barrier integrity [37]. One recent study also reported that CD2AP was required for maintaining neuronal structure and function [38]. These findings, together with the finding that *CD2AP* expression was decreased in peripheral blood lymphocytes in LOAD patients [39], implicate that CD2AP deficiency may contribute to AD pathogenesis. However, some other studies found that overexpression of CD2AP promoted APP cleavage and that downregulation of CD2AP reduced cell surface levels of APP and Aβ release in cell cultures [40, 41]. Moreover, haploinsufficiency of CD2AP in one AD model mice had no effect on Aβ deposition in the brain [41]. Therefore, detailed functions of CD2AP in different brain cells should be determined to fully establish the role of CD2AP in AD.

In the present study, we found that CD2AP was abundantly expressed in microglia and its level significantly increased in the brain of AD patients and AD model mice at pathological stages. Haploinsufficiency of microglial *Cd2ap* alleviated cognition and synaptic plasticity impairments in the 5xFAD model of amyloid deposition. Microglial CD2AP deficiency also attenuated Aβ-induced microgliosis and disease-associated microglia (DAM) generation. Moreover, CD2AP deficiency in microglia compromised their uptake ability. Finally, we found that CD2AP interacted with and regulated cell surface levels of colony stimulating factor 1 receptor (CSF1R) that is essential for microglial function [19, 42–44].

## Methods

### Animals

*Cd2ap*^*fl/*+^ mice (C57BL/6 background) were created by Cyagen Biosciences using CRISPR/Cas9-mediated genome engineering. Briefly, a homology region covering mouse *Cd2ap* exon 4 and adjacent sequences was subcloned into the targeting vector. Both *Cd2ap* intron 3 and intron 4 were introduced with a Loxp site. The target vector, a gRNA to *Cd2ap*, and the Cas9 mRNA were co-injected into fertilized mouse eggs to generate targeted conditional knockout offspring (Supplemental Fig. 3A). PCR primers were designed to amplify a region containing the Loxp site in intron 3 for mouse genotyping; and they amplify a 109-bp fragment for the WT allele and a 168-bp fragment for the flox allele (Supplemental Fig. 3B). *Cd2ap*^*fl/*+^ mice were crossed with *Cx3cr1*-cre mice (from Cyagen Biosciences, C001032) to generate *Cd2ap*^*fl/*+^;*Cx3cr1*-cre mice that have CD2AP haploinsufficiency specifically in microglia. These mice were then crossed with 5xFAD mice (from Jackson Lab, 034848-JAX) to generate *Cd2ap*^*fl/*+^, *Cd2ap*^*fl/*+^;*Cx3cr1*-cre, 5xFAD;*Cd2ap*^*fl/*+^, and 5xFAD;*Cd2ap*^*fl/*+^;*Cx3cr1*-cre mice. Alternatively, *Cd2ap*^*fl/*+^;*Cx3cr1*-cre were crossed with *Cd2ap*^*fl/*+^ mice to generate *Cd2ap*^*fl/fl*^;*Cx3cr1*-cre mice and their control (*Cd2ap*^*fl/fl*^) mice. Both male and female mice were used unless otherwise specified. All mice were housed in a pathogen-free environment with a 12-h light/day cycle and with ad libitum access to food and water. Primer sequences used for genotyping were listed as follows. For *Cd2ap*^*fl/*+^, forward primer: 5’-CAAGAAGCCTCCTGGTTATAGCATC-3’; reverse primer: 5’-CAGCACAGACACTAGCACTCCTAC-3’. For *Cx3cr1*-cre, forward primer: 5’-GACATTTGCCTTGCTGGAC-3’; reverse primer: 5’-GCAGGGAAATCTGATGCAAG-3’. For 5xFAD, forward primer: 5’-AATAGAGAACGGCAGGAGCA-3’; reverse primer: 5’-GCCATGAGGGCACTAATCAT-3’.

### Microglia depletion

Pexidartinib (PLX3397, Selleck, S7818) was mixed in rodent chow (300 mg PLX3397 per kilogram of diet), and continuously provided to the animals ad libitum for 1 month at 4 months of age.

### Western blotting and antibodies

Protein lysates of brain tissues or cell cultures were separated by SDS–polyacrylamide gel, transferred onto polyvinylidene difluoride membranes, and immunoblotted with indicated antibodies. Primary antibodies used were: anti-APP (Millipore, mab348, 1:1000), anti-CD2AP (Proteintech, 51046–1–AP, 1:2000), anti-GAPDH (Abway, ab0038, 1:10000), anti-NeuN (Abcam, ab177487, 1:1000), anti-GFAP (Cell Signaling Technology, 3670S, 1:1000), anti-Iba1 (Wako, 016–20001, 1:1000), anti-β-actin (Cell Signaling Technology, 8457S, 1:10000), anti-CX3CR1 (Abclonal, a2890, 1:1000), anti-CSF1R (Abcam, ab254357, 1:2000), anti-CD11b (Biolegend, 101202, 1:1000), anti-p-ERK (Cell Signaling Technology, 4370, 1:1000), anti-C1q (Hycult Biotech, HM1096BT, 1:1000), anti-CD93 (Abcam, ab134079), anti-MYC (Cell Signaling Technology, 2276S, 1:2000), anti-HA (Proteintech, 51064–2-AP, 1:2000), and anti-ERK (Cell Signaling Technology, 4695, 1:1000), Secondary antibodies used were: Goat anti-Rabbit IgG (H + L) Secondary Antibody, HRP (Thermo Fisher Scientific, 31460, 1:5000), and Goat anti-Mouse IgG (H + L) Secondary Antibody, HRP (Thermo Fisher Scientific, 31430, 1:5000).

### Cell cultures and treatments

HEK293T cells were cultured in Dulbecco’s modified Eagle’s medium (DMEM) containing 10% fetal bovine serum (FBS). Mouse primary neurons, microglia, and astrocytes were prepared and cultured as reported previously [45]. In some experiments, cultured primary microglia were treated with or without CSF1 (R&D Systems, 415-ML, 100 ng/ml) for 24 h before further studies.

### Behavioral analysis

Mice at 7 months of age were sequentially subjected to behavioral tests including Y maze, T maze, Morris water maze, and novel object recognition, following protocols reported previously [46, 47]. Each test was conducted on different days. All behavioral tests were carried out between 9:00 am and 5:00 pm in a blinded manner. Mice were touched three days before the start of the experiment to allow them to acclimatize.

### Electrophysiology

Following anesthesia with isoflurane, mice at 7 months of age were rapidly decapitated. The brain was dissected and cut into slices (400 μm thick). Brain slices were subjected to long-term potentiation (LTP) recording at the hippocampal CA3-CA1 Schaffer’s collateral pathway, following a protocol previously described [46]. Both male and female mice were used for electrophysiological analysis.

### RNA-seq and data analysis

Total RNA was isolated from hippocampal tissues of 7-month-old male mice using the TRIzol reagent (Yeasen, 10606ES60). RNA-seq and data processing were carried out by Beijing Genomics Institute (BGI, Shenzhen, China) using standard protocols. Analyses of RNA-seq data, including differentially expressed gene identification (log2 (fold change) > 0.3; *P* < 0.05), Gene Ontology search, Venn diagram comparison, and heatmap drawing were carried out using Dr. TOM, an in-house customized data mining system of the BGI. The RNA-seq data have been deposited into CNGB Sequence Archive (CNSA) [48] of China National GeneBank DataBase (CNGBdb) [49] with the accession number CNP0004099.

### Quantitative real-time PCR (qRT-PCR)

RNA isolation, reverse transcription, and qRT-PCR were performed as previously described [47]. Primers used were as follows. For *C1qa*, Forward: 5’-CTGGCATCCGGACTGGTATC-3’; Reverse: 5’-CTTTCACGCCCTTCAGTCCT-3’. For *C1qb*, Forward: 5’- CCAGGATTCCATACACAGGAAGC-3’; Reverse: 5’- CCAAACTCACCAAGGTCTCCA-3’. For *C1qc*, Forward: 5’- AGTCCCTTACACCCTCAGGA -3’; Reverse: 5’-GGCTGGGATTCCTGGCTCT -3’. For *Cx3cr1*, Forward: 5’-TTCCCATCTGCTCAGGACCTC-3’; Reverse: 5’- AGACCGAACGTGAAGACGAG-3’. For *Trem2,* Forward: 5’- GTCCCAAGCCCTCAACACC-3’; Reverse: 5’-TCCTCACCCAGCTGCCGACA -3’. For *Cd68*, Forward: 5’-ATCCCCACCTGTCTCTCTCA-3’; Reverse: 5’- ACCGCCATGTAGTCCAGGTA-3’. For *Itgax*, Forward: 5’- CTGGATAGCCTTTCTTCTGCTG-3’; Reverse: 5’- GCACACTGTGTCCGAACTCA-3’. For *Clec7a*, Forward: 5’- ATGGTTCTGGGAGGATGGAT-3’; Reverse: 5’-CCTGGGGAGCTGTATTTCTG -3’. For *Ccl6*, Forward: 5’-TCAAGCCGGGCATCATCTTT-3’; Reverse: 5’- CTGCCCTCCTTCTCAAGCAA-3’. For *Cd9*, Forward: 5’- TACCATGCCGGTCAAAGGAG-3’; Reverse: 5’- GGAACCCGAAGAACAATCCC -3’. For *Csf1r*, Forward: 5’-AATCCACCTCCACTGGCATC -3’; Reverse: 5’- GGGGATTTCCAGCTTGGTGT-3’. For *Gapdh*, Forward: 5’- TGTCCGTCGTGGATCTGAC-3’; Reverse: 5’- CCTGCTTCACCACCTTCTTG-3’. For *Tnfa*, Forward: 5’-GTCTACTGAACTTCGGGGTGAT-3’; Reverse: 5’- CTGAGTGTGAGGGTCTGGGC-3’. For *Il1b*, Forward: 5’- CAGGCAGGCAGTATCACTCATTG-3’; Reverse: 5’- GCTTTTTTGTTGTTCATCTCGGA-3’. For *Il6*, Forward: 5’- CAATGGCAATTCTGATTGTATG-3’; Reverse: 5’- AGGACTCTGGCTTTGTCTTTC-3’. For *Arg1*, Forward: 5’- CACAGTCTGGCAGTTGGAAGC-3’; Reverse: 5’- CTTTGGCAGATATGCAGGGAG-3’. For *Ym1*, Forward: 5’- CAGGTCTGGCAATTCTTCTGAA-3’; Reverse: 5’- GTCTTGCTCATGTGTGTAAGTGA-3’.

### Thioflavin S staining

Mouse brain slices were incubated with 1 mg/ml Thioflavin S (Sigma, T1892-25G) for 10 min. After washing with 70% ethanol, Thioflavin S-labeled amyloid plaques were imaged with the Olympus FV1000MPE-B confocal microscope.

### Immunofluorescence staining

Mouse brain slices or cultured cells were permeabilized, incubated with primary antibodies at 4 °C overnight, incubated with appropriate fluorescence-labeled secondary antibodies for 1 h at room temperature, stained with DAPI for 10 min, and observed under a confocal microscope. Images were further analyzed by ImageJ software (National Institutes of Health). Primary antibodies used included: anti-Iba1 (Wako, 019–19741, 1:200), anti-GFAP (Cell Signaling Technology, 3670S, 1:200), anti-mDectin1 (Invivogen, mabg-mdect, 1:200), anti-Aβ (6E10, Biolegend, 803014, 1:200), anti-CD68 (Biolegend, 137001, 1:200), anti-LAMP1 (Abcam, ab24170, 1:200), anti-synapsin 1 (Proteintech, 20258–1–AP, 1:200), anti-PSD95 (Millipore, MABN1194, 1:200), and anti-Ki-67 (Abcam, ab16667, 1:200). Secondary antibodies used included: Alexa Fluor 488 Goat anti-Rabbit IgG (Thermo Fisher Scientific, A-11008, 1:500), Alexa Fluor 488 Goat anti-mouse IgG (Thermo Fisher Scientific, A-11001, 1:500), Alexa Fluor 594 Goat anti-Rabbit IgG (Thermo Fisher Scientific, A-11012, 1:500), Alexa Fluor 594 Goat antiMouse IgG (Thermo Fisher Scientific, A-11005, 1:500), and Alexa Fluor 647 Donkey anti-Rat IgG (Abcam, Ab150155, 1:500).

### Aβ plaque analysis

The analysis of total area and average size of Aβ plaques and Aβ compaction was performed following previously reported methods [50]. Briefly, the Aβ or ThioS fluorescent signal within the analysis area was adjusted to an appropriate threshold using Image J, and the total plaque area, as well as the area of individual plaques, was measured. The plaques were categorized into three sizes: small (1–20 μm^2^), medium (20–200 μm^2^), and large (> 200 μm^2^). The average plaque area was then calculated based on the areas of the individual plaques. Aβ compaction was evaluated by determining the ratio of Thioflavin S–stained (ThioS-positive) plaque area over the anti-Aβ antibody 6E10-immunostained (6E10-positive) plaque area, incorporating assessments at both the global and individual scales.

### Microglial morphology reconstruction

3D reconstruction of microglial morphology and analysis of engulfed PSD95^+^ puncta were performed as previously described [45]. Briefly, after immunofluorescence staining, each image field was captured in a z-stack direction with 1 μm step size for 15 pictures. Pictures were superimposed into one representative 2D image to show microglial branches. Microglia images were reconstructed into 3D using the Imaris 9.2.0 software (Bitplane). The Imaris function “Fliaments” was used to quantify process length and the number of branch points. The Imaris function “Surface” was used to assess the numbers of PSD95^+^ puncta entirely within the microglial volume but outside the nucleus region.

### Microglial cytoskeleton assay

To visualize microglial cytoskeleton, cultured microglia were stained with fluorescence-labeled phalloidin (Beyotime Biotechnology, C2201S, diluted at 1:200 with PBS contained 2% BSA and 0.1% Triton X-100), following the manufacturer’s protocol. Cells were counterstained with DAPI before image capture using Olympus FV1000MPE-B. Images were further analyzed by ImageJ software.

### Aβ42 ELISA

Sample preparation was performed as previously described [46]. Aβ42 levels were quantified using a human ELISA kit (Thermo Fisher Scientific, KHB3441), following the manufacturer’s protocol.

### Stereotactic injection of oAβ42 oligomer (oAβ42)

TAMRA-Aβ42 (Anaspec, AS-60476) was oligomerized following previously reported protocols [51]. Stereotactic injection of oAβ42 was performed as previously described [52]. Briefly, 2-month-old *Cd2ap*^*fl/*+^ mice and *Cd2ap*^*fl/*+^;*Cx3cr1*-cre littermates were anesthetized and TAMRA-oAβ42 (1.5 μg in 2 μL vehicle/side) was bilaterally injected into the hippocampus at the following coordinates: anteroposterior, − 2.0 mm from the bregma; mediolateral, ± 2.0 mm; dorsoventral, − 1.8 mm. Mice were sacrificed sixteen hours after injection and immunofluorescence staining was performed.

### Golgi staining

Golgi staining was performed using the FD Rapid Golgi Stain™ kit (FD Neuro Technologies, PK401). After staining and sealing, Z-stack images of the secondary branches of pyramidal neurons located in the CA1 subfield of the hippocampus were obtained with a laser scanning confocal microscope (Olympus, FV1000). At least 13–20 branches (with at least 10 μm of length) of neurons were randomly observed in each mouse for spine density counting using Image J.

### Uptake assay

Synaptosomes were prepared and conjugated with the pHrodo red dye (Thermo Fisher Scientific, P36600) as described previously [45]. For uptake activity assays, pHrodo red dye-labeled synaptosomes, pHrodo green *E. coli* Bioparticles (Thermo Fisher Scientific, P35366), FAM-oAβ, or TAMRA-oAβ were incubated with cultured primary microglia for 1 h. In some experiments, the proteasome inhibitor MG132 and the lysosome inhibitor chloroquine were added 30 min before the phagosomes and microglial cells were incubated to inhibit protein degradation. After washing off unengulfed objects with 1xPBS, microglia were subjected to either immunofluorescence staining or flow cytometry analysis.

### Biotinylation assays

Cell surface protein biotinylation was performed as described previously [45].

### Biomolecular Fluorescence Complementation (BiFC) assay

VC-CSF1R and VN-CD2AP plasmids were generated by inserting target gene fragments amplified by PCR into the pBIFC-VC155 and pBIFC-VC173 vectors, respectively. Primer sequences used for plasmid construction were the following. For VC-CSF1R, forward: 5’-ACCTCCATAGAAGATTCTAGAATGGGCCCAGGAGTTCTGC-3’; reverse: 5’-GTTCTTCTGCTTGTCTCTAGAGCAGAACTGATAGTTGTTGGGCT-3’. For VN-CD2AP, forward: 5’-ACCTCCATAGAAGATTCTAGAATGGTTGACTATATTGTGGAGTATGACTAT-3’; reverse: 5’-GCCCTTGCTCACCATTCTAGAAGAAGACAGGACAGCTTTTTTCAGC-3’. The two plasmids and control plasmid were transfected into HEK293T cells individually or collectively, using the TurboFect Transfection Reagent (Thermo Fisher Scientific, R0534). 48 h after transfection, cells were fixed in 4% PFA, stained with DAPI, and observed under the Olympus FV1000MPE-B microscope.

### Co-immunoprecipitation

CSF1R-HA and CD2AP-MYC plasmids were generated by inserting target gene fragments into the pcDNA 3.1-HA and pcDNA 3.3-MYC vectors, respectively. The two plasmids and control plasmid were transfected into HEK293T cells individually or collectively, using the TurboFect Transfection Reagent (Thermo Fisher Scientific, R0534). 24 h after transfection, cells were collected. For co-immunoprecipitation, cells or mouse brain tissues were lysed in a TNEN buffer (50 mM Tris–HCl [pH 8.0], 150 mM NaCl, 2 mM EDTA, and 1% Nonidet P-40) supplemented with protease inhibitor (MedChemExpress, HY-K0010). Equal amounts of cell or brain tissue lysates, antibodies, and Protein A/G MagBeads (Yeasen, 36417ES08) were co-incubated at 4 °C overnight. Immunocomplexes and 10% amounts of cell or brain tissue lysates for immunoprecipitation (as input) were analyzed by western blot.

### Statistics

Statistical analysis was performed using GraphPad Prism software (version 8). For comparisons between 2 groups, unpaired Student’s *t* test was used. For comparisons among 3 or 4 groups, 1-way or 2-way ANOVA followed by appropriate corrections were used. Detailed statistical method for each comparison is provided in corresponding figure legend. Correlation analysis was performed by Spearman correlation analysis. Data are presented as mean ± SEM. Statistical significance was defined as *P* < 0.05.

## Results

### CD2AP expression is enriched in microglia and increased in the brain of AD patients and 5xFAD mice

**Fig. 1:**
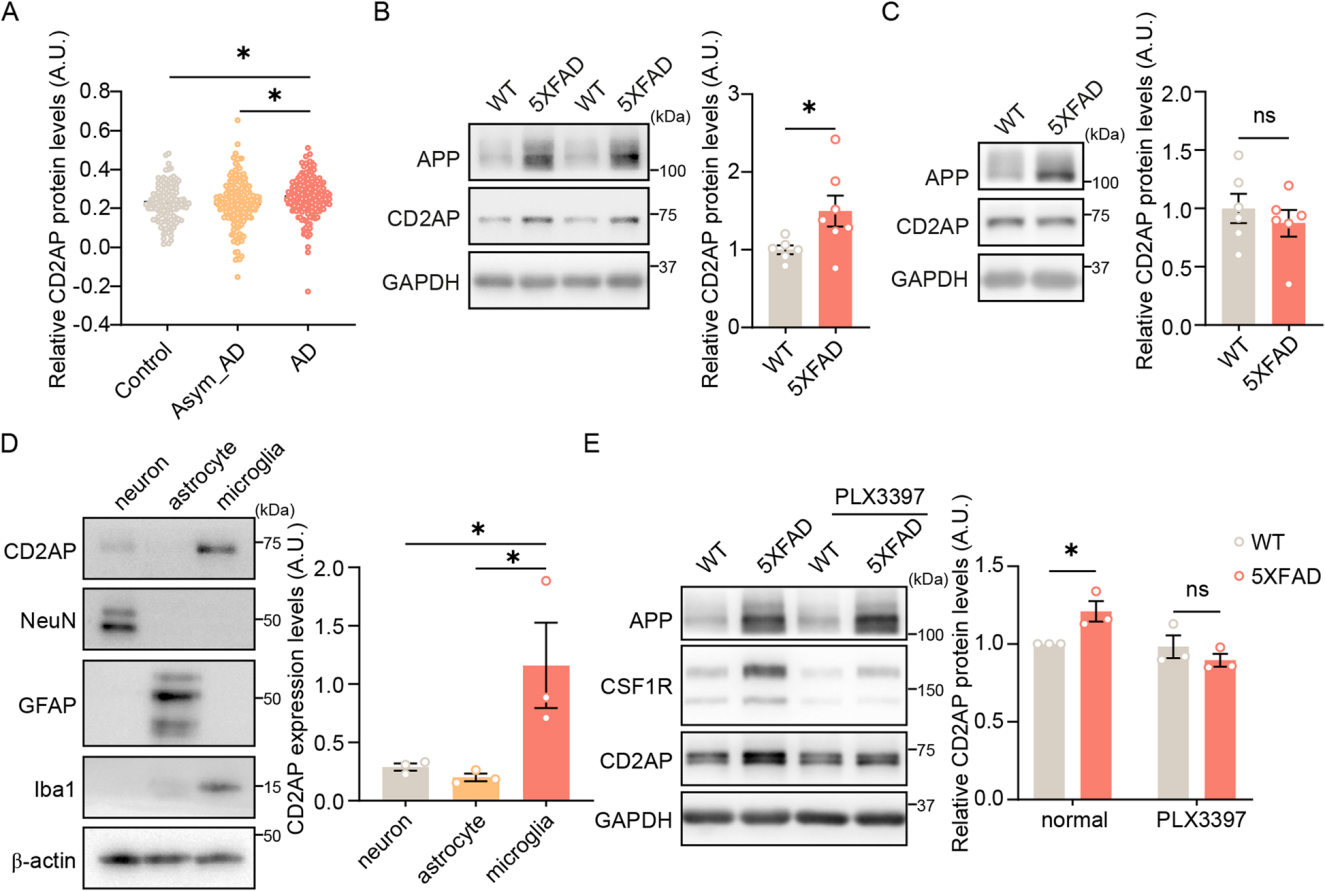
CD2AP expression is increased in the brain of AD patients and 5xFAD mice. **A** Comparison of CD2AP protein levels in the brain of AD patients (*n* = 173), asymptomatic AD (Asym_AD, *n* = 177) patients, and control subjects (*n* = 117) from a proteomic study [53]. 1-way ANOVA with two-sided Holm correction. **B** CD2AP protein levels in hippocampal tissues of WT (*n* = 6) and 5xFAD (*n* = 7) mice at 7 months of age were analyzed by western blot and quantified for comparison. Unpaired Student’s *t* test. **C** CD2AP protein levels in hippocampal tissues of WT and 5xFAD mice at 1.5 months of age were analyzed by western blot and quantified for comparison. *n* = 6 per group. Unpaired Student’s *t* test. **D** CD2AP protein levels in cultured primary neurons, microglia, and astrocytes were analyzed by western blot and quantified for comparison. NeuN, GFAP, and Iba1 were markers for neurons, astrocytes, and microglia, respectively. *n* = 3. 1-way ANOVA followed by Dunnett's post hoc test. **E** CD2AP protein levels in hippocampal tissues of 5-month-old WT and 5xFAD mice treated with or without PLX3397 were analyzed by western blot and quantified for comparison. CSF1R was studied to indicate the effect of PLX3397 treatment. *n* = 3. 2-way ANOVA followed by Sidak’s post hoc test. Data are presented as mean ± SEM. *P < 0.05; ns, not significant

To investigate the role of CD2AP in AD, we first checked CD2AP protein levels reported in a proteomic study with large datasets [53] and found that CD2AP was significantly increased in the brain of AD patients compared to control subjects and asymptomatic AD patients (Fig. 1A). The protein sequence identity between human and mouse CD2AP is about 86%. We then studied and found that CD2AP levels were also significantly increased in the hippocampal tissues of 5- (Fig. 1E) and 7-month-old (Fig. 1B), but not those of 1.5–2 month-old (Fig. 1C and Supplemental Fig. 7A) 5xFAD mice, an AD model carrying five familial AD mutations [54], suggesting that the change of CD2AP expression in the hippocampal tissues of 5xFAD mice is dynamic and dependent on amyloid pathology development. Moreover, we found that CD2AP was expressed much higher in cultured mouse primary microglia than neurons and astrocytes (Fig. 1D). In an open database originally generated by Dr. Ben Barres and collaborators (www.brainrnaseq.org; [55, 56]), we also noticed that *CD2AP* expression levels were highly enriched in microglia compared to other cell types in the human brain (Supplemental Fig. 1). To explore whether increased CD2AP levels in the hippocampus of 5xFAD mice are mainly attributed to their change in microglia, we fed 5xFAD mice with the CSF1R inhibitor, pexidartinib (PLX3397) to eliminate microglia and indeed found that the increase of CD2AP in the hippocampus of 5xFAD mice was abolished by PLX3397 treatment (Fig. 1E and Supplemental Fig. 2). These results indicate that CD2AP is upregulated in microglia in the setting of amyloid deposition.

### Haploinsufficiency of CD2AP in microglia attenuates cognition and synaptic plasticity deficits in 5xFAD mice

We generated *Cd2ap* conditional knockout mice (*Cd2ap*^*fl/*+^, Supplemental Fig. 3A and B). By crossing them with *Cx3cr1*^*Cre/*+^ mice and then with 5xFAD mice, we acquired *Cd2ap*^*fl/*+^, 5xFAD;*Cd2ap*^*fl/*+^, *Cd2ap*^*fl/*+^;*Cx3cr1*-cre, and 5xFAD;*Cd2ap*^*fl/*+^;*Cx3cr1*-cre mice, with the latter two having microglial CD2AP haploinsufficiency (Supplemental Fig. 3C). By comparing CD2AP protein levels in cultured primary neurons, astrocytes, and microglia derived from *Cd2ap*^*fl/*+^ and *Cd2ap*^*fl/*+^;*Cx3cr1*-cre mice, we found that CD2AP levels were significantly reduced by ~ 50% in microglia (Supplemental Fig. 3D), but not altered in neurons (Supplemental Fig. 3E) or astrocytes (Supplemental Fig. 3F) with only one copy of *Cd2ap*, confirming a specific microglial heterozygous deletion of *Cd2ap* in *Cd2ap*^*fl/*+^;*Cx3cr1*-cre mice.

**Fig. 2:**
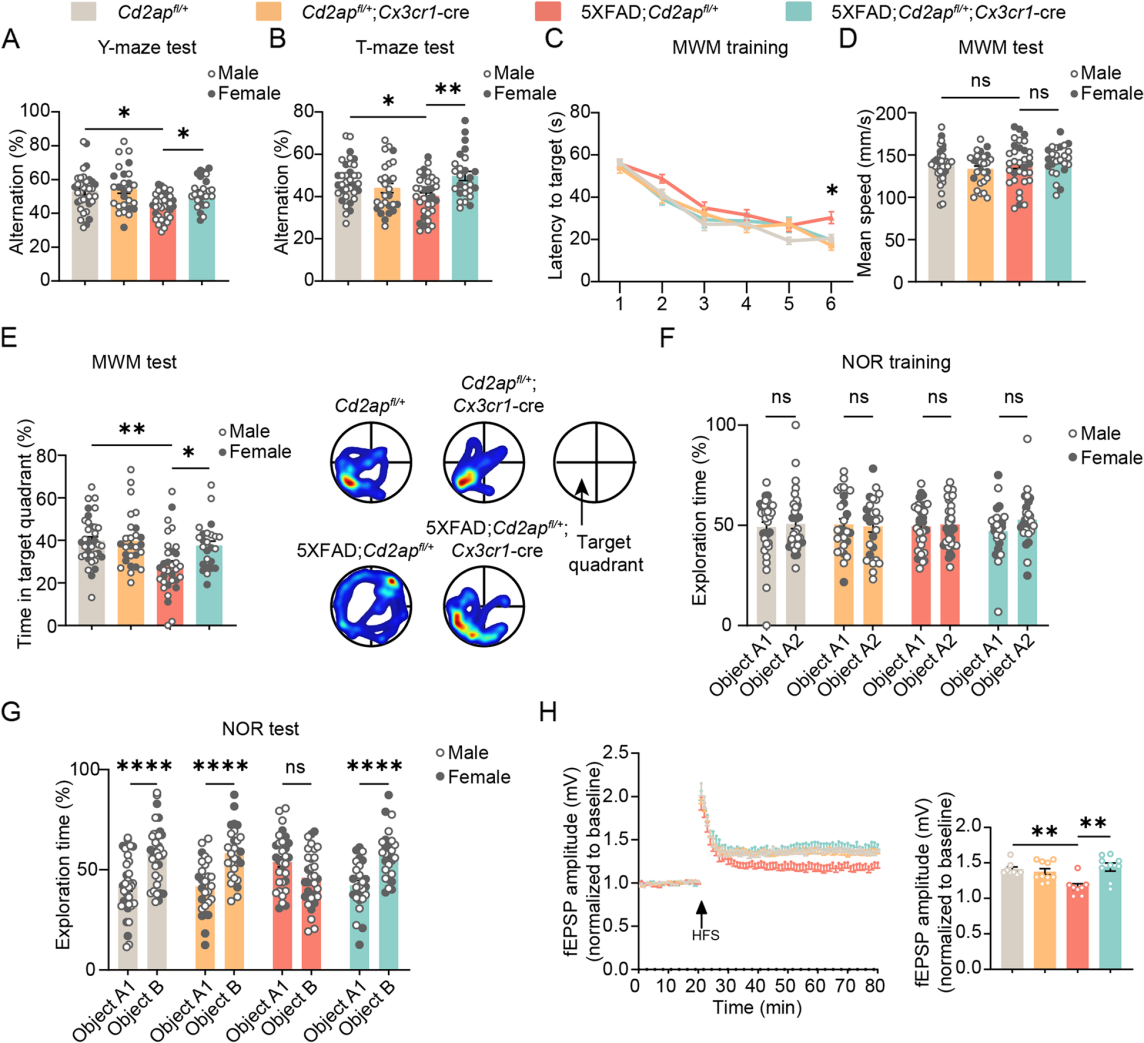
Microglial CD2AP haploinsufficiency attenuates cognitive and synaptic deficits in 5xFAD mice. **A**, **B** In the Y- (**A**) and T-maze (**B**) tests, the percentages of spontaneous alternation were analyzed in 7-month-old *Cd2ap*^*fl/*+^ (*n* = 23 males, *n* = 12 females), *Cd2ap*^*fl/*+^;*Cx3cr1*-cre (*n* = 17 males, *n* = 10 females), 5xFAD;*Cd2ap*^*fl/*+^ (*n* = 22 males, *n* = 12 females), and 5xFAD;*Cd2ap*^*fl/*+^;*Cx3cr1*-cre (*n* = 14 males, *n* = 12 females) mice. 2-way ANOVA followed by Tukey’s post hoc test. **C**-**E** In the Morris water maze (MWM) test, latency to target during a 6-day training (**C**), and mean speed (**D**) and percentage of time spent in the target quadrant (**E**) during the test on the 7th day were analyzed in 7-month-old *Cd2ap*^*fl/*+^ (*n* = 23 males, *n* = 12 females), *Cd2ap*^*fl/*+^;*Cx3cr1*-cre (*n* = 17 males, *n* = 10 females), 5xFAD;*Cd2ap*^*fl/*+^ (*n* = 21 males, *n* = 12 females), and 5xFAD;*Cd2ap*^*fl/*+^;*Cx3cr1*-cre (*n* = 13 males, *n* = 12 females) mice. 2-way ANOVA followed by Tukey’s post hoc test. **F**, **G** In the novel object recognition (NOR) test, the percentages of exploration time to objects A1 and A2 during the training phase (**F**) and to objects A1 and B during the test phase (**G**) were analyzed in 7-month-old *Cd2ap*^*fl/*+^ (*n* = 23 males, *n* = 12 females), *Cd2ap*^*fl/*+^;*Cx3cr1*-cre (*n* = 17 males, *n* = 10 females), 5xFAD;*Cd2ap*^*fl/*+^ (*n* = 19 males, *n* = 12 females), and 5xFAD;*Cd2ap*^*fl/*+^;*Cx3cr1*-cre (*n* = 14 males, *n* = 12 females) mice. 2-way ANOVA followed by Tukey’s post hoc test. **H** LTP in the hippocampal Schaffer’s collaterals was induced by high frequency stimulation (HFS) and fEPSP slopes in the CA1 region was recorded in acute slices from 7-month-old mice. fEPSP amplitude values of the last 10 min of LTP recording were averaged for comparison. *Cd2ap*^*fl/*+^: *n* = 8 slices from 8 mice; *Cd2ap*^*fl/*+^;*Cx3cr1*-cre, *n* = 10 slices from 4 mice; 5xFAD;*Cd2ap*^*fl/*+^: *n* = 8 slices from 4 mice; and 5xFAD;*Cd2ap*^*fl/*+^;*Cx3cr1*-cre: *n* = 9 slices from 4 mice. 1-way ANOVA followed by Tukey’s post hoc test. Data are presented as mean ± SEM. *P < 0.05; **P < 0.01; ****P < 0.0001; ns, not significant

We next carried out behavioral studies in the order of Y maze, T maze, Morris water maze, and novel object recognition tests in mice at 7 months of age to assess the effects of microglial CD2AP haploinsufficiency on AD-related cognitive dysfunction (Supplemental Fig. 4A). When both male and female mice were combined for analysis, we found that 5xFAD mice had reduced spontaneous alternation compared to controls in Y- and T-maze tests. While microglial CD2AP haploinsufficiency attenuated the reducion of spontaneous alternation in 5xFAD mice in both tests (Fig. 2A and B). In the Morris water maze test, 5xFAD mice showed elevated latency to the target platform on the 6th day of the training and decreased time spent in the target quadrant during the test phase on the 7th day, whereas both of which were improved in 5xFAD mice haploinsufficient for microglial CD2AP (Fig. 2C and E). No mean swim speed differences were found between different groups of mice (Fig. 2D). In the novel object recognition test, 5xFAD mice were unable to distinguish novel and familiar objects; and this was improved by microglial CD2AP haploinsufficiency (Fig. 2F and G). These results confirm impaired learning and memory in 5xFAD mice and more importantly, demonstrate that microglial CD2AP haploinsufficiency can rescue these impairments.

When male and female mice were analyzed separately (Supplemental Fig. 4B-M), there were some differences between the results. Female 5xFAD mice showed no changes in their spontaneous alternation in Y- and T-maze tests and their latency to the target during the training phase in the Morris water maze test (Supplemental Fig. 4B-D). But they had reduced time spent in the target quadrant during the Morris water maze testing phase (Supplemental Fig. 4F) and were unable to distinguish novel and familiar objects in the novel object recognition test (Supplemental Fig. 4G), of which the latter was reversed by microglial CD2AP haploinsufficiency. While male mice showed impaired learning and memory in all these behavioral tests (Supplemental Fig. 4H-M); and their increased latency to the target in the training phase during the Morris water maze test, reduced time spent in the target quadrant during the Morris water maze testing phase, and their inability to distinguish novel and familiar objects in the novel object recognition test were rescued by microglial CD2AP haploinsufficiency. Altogether, these results suggest that disease-like phenotypes in female 5xFAD mice are slightly less than in male 5xFAD mice at the age studied and that microglial CD2AP haploinsufficiency generally exerts protection in both sexes.

We also evaluated synaptic plasticity in these mice by electrophysiological studies. The results showed that 5xFAD mice had impaired LTP in the hippocampal CA3-CA1 pathway and this was rescued by microglial CD2AP haploinsufficiency (Fig. 2H). Notably, both behavioral and electrophysiological studies revealed no differences between *Cd2ap*^*fl/*+^ mice and *Cd2ap*^*fl/*+^;*Cx3cr1*-cre mice, suggesting that microglial *Cd2ap* deficiency has no effect on the physiological activity of mice at non-disease state.

### Microglial CD2AP haploinsufficiency attenuates microglia activation in 5xFAD mice

**Fig. 3:**
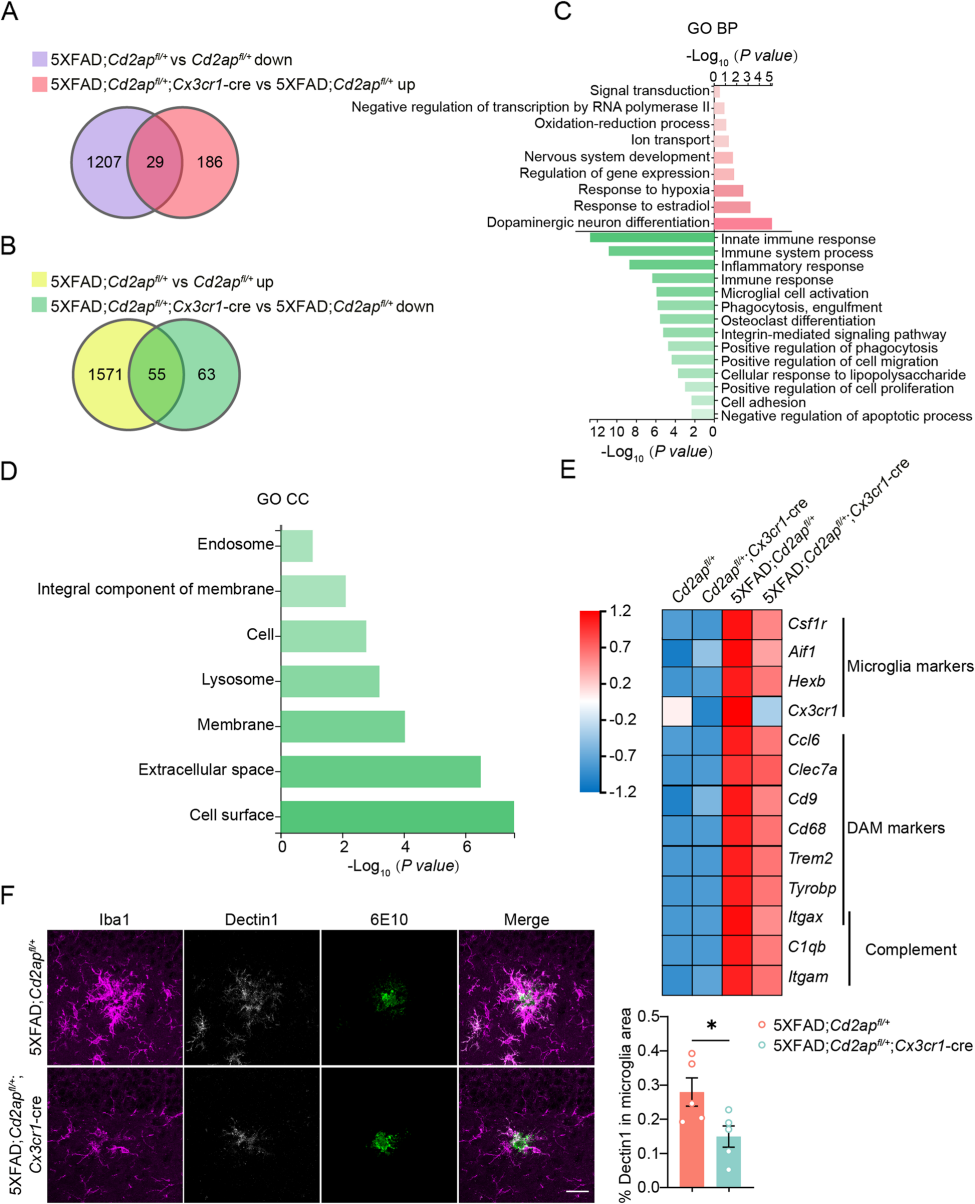
Microglial CD2AP haploinsufficiency attenuates microglia activation in 5xFAD mice. **A**, **B** Venn diagram analysis revealed that the expressions of 29 downregulated genes (**A**) and 55 upregulated genes (**B**) in 5xFAD mice compared to controls were reversed when microglial CD2AP was deficient. **C** Important Gene Ontology terms for Biological Process (GO BP) based on the 29 genes from (**A**, in pink) and the 55 genes from (**B**, in green). **D** Important Gene Ontology terms for Cellular Component (GO CC) based on the 55 genes from (**B**). **E** Gene expression heatmap of selected microglia-related genes identified in the 55 genes from (**B**). **F** Representative images showing co-immunostaining of Iba1 (magenta), Dectin1 (white), and 6E10 (green) in the hippocampus of 7-month-old 5xFAD;*Cd2ap*^*fl/*+^ and 5xFAD;*Cd2ap*^*fl/*+^;*Cx3cr1*-cre mice. Scale bars: 20 μm. The percentages of Dectin1^+^ microglia in total microglia were compared. *n* = 5 mice per genotype. Unpaired Student’s *t* test. Data are presented as mean ± SEM. *P < 0.05

We carried out RNA-seq using hippocampal tissues from the four groups of male mice at 7 months of age to identify genes whose expression change in 5xFAD mice can be reversed by microglial CD2AP haploinsufficiency, as these genes may be crucial for mediating the protection by microglial CD2AP haploinsufficiency. We identified 29 genes whose expression was downregulated in 5xFAD;*Cd2ap*^*fl/*+^ versus *Cd2ap*^*fl/*+^ but upregulated in 5xFAD;*Cd2ap*^*fl/*+^;*Cx3cr1*-cre versus 5xFAD;*Cd2ap*^*fl/*+^ (Fig. 3A). Enrichment analysis of gene ontology terms for the biological process (GO BP) of the 29 genes showed that some of them were enriched in processes involved in neuronal activity (Fig. 3C), implicating that neuronal function is impaired in 5xFAD mice and improved by microglial CD2AP haploinsufficiency. We also identified 55 genes whose expression was upregulated in 5xFAD;*Cd2ap*^*fl/*+^ versus *Cd2ap*^*fl/*+^ but downregulated in 5xFAD;*Cd2ap*^*fl/*+^;*Cx3cr1*-cre versus 5xFAD;*Cd2ap*^*fl/*+^ (Fig. 3B). Interestingly, GO BP enrichment analysis revealed that these genes were largely enriched in processes related to immune responses (Fig. 3C), implying that increased immune responses mainly caused by aberrant microglial activity in 5xFAD mice can be attenuated by microglial CD2AP haploinsufficiency, thereby directly linking microglial CD2AP deficiency to microglial activity. GO cellular component (GO CC) enrichment analysis also suggested that many of the 55 genes identified in Fig. 3B were cell surface components (Fig. 3D) and this is consistent with the notion that signal transduction through cell surface receptors are crucial for regulating microglial activity. Moreover, the dramatically increased expression of many microglial marker genes (*Csf1r*, *Aif1*, *Hexb*, and *Cx3cr1*), DAM marker genes (*Ccl6*, *Clec7a*, *Cd9*, *Cd68*, *Trem2*, *Tyrobp*, and *Itgax*), and complement pathway genes (*C1qb*, *Itgam*, and *Itgax*) in 5xFAD mice at 7 months of age was reversed by microglial CD2AP deficiency (Fig. 3E).

DAM cells are generated and distributed spatially specifically around Aβ plaques with the progression of amyloidosis [18]. Although the gene signature associated with DAM has generally been associated with neuroprotection, some recent studies have shown that reducing cells with the DAM signature may be protective in mouse models with AD-type pathology [57, 58]. Herein, we further studied the expression change of DAM markers by qRT-PCR and confirmed the RNA-seq results in the four groups of mice (Supplemental Fig. 5C-H). Given that *Cx3cr1*-Cre may lead to DAM activation [59, 60], we also studied *Cx3cr1*-Cre mice and 5xFAD;*Cx3cr1*-Cre mice as additional controls. As previously reported [61], *Cx3cr1* expression was significantly decreased in *Cre*-containing mice compared to non-*Cre*-containing mice; and this is probably due to a heterozygosity of *Cx3cr1* expression resulting from the *Cre* transgene insertion (Supplemental Fig. 5A). However, a decreased *Cx3cr1* expression seemed to have no effect on CX3CR1 protein levels in mice carrying *Cx3cr1*-cre (Supplemental Fig. 5B). More importantly, Microglial CD2AP haploinsufficiency did not further reduce *Cx3cr1* expression in mice carrying *Cx3cr1*-cre (Supplemental Fig. 5A) and *Cx3cr1*-cre had no effect on the expresion of DAM-associated genes detected here except *Itgax*, for which expression was increased rather than decreased (Supplemental Fig. 5C-H). Therefore, *Cx3cr1*-cre may only minimally interfere with the effect of CD2AP haploinsufficiency on microglial activities.

We also labeled DAM by co-immunostaining Dectin1 (the protein encoded by *Clec7a*) and the microglial marker Iba1 with Aβ plaques, and found significantly fewer Dectin1-labeled microglia in 5xFAD;*Cd2ap*^*fl/*+^;*Cx3cr1*-cre mice than in 5xFAD;*Cd2ap*^*fl/*+^ mice (Fig. 3F). These results further suggest that microglial CD2AP haploinsufficiency may inhibit the generation of DAM.

Although the expression of pro-inflammatory factors (*Il1b*, *Il6*, and *Tnfa*) was significantly increased in 5xFAD mice at pathological stages as expected, we found that microglial CD2AP haploinsufficiency did not reverse their expression (Supplemental Fig. 6A). Instead, microglial CD2AP haploinsufficiency reversed the expression reduction of *Arg1* and increased the expression of *Ym1* in 7-month-old 5xFAD mice (Supplemental Fig. 6B). *Arg1* and *Ym1* are markers for the anti-inflammatory M2 type of microglia. Therefore, there is a possibility that microglial CD2AP haploinsufficiency improves the inflammatory environment in the brain of 7-month-old 5xFAD mice, and this deserves further scrutiny.

On the other hand, when CD2AP levels were not yet significantly increased in 2-month-old 5xFAD mice (Supplemental Fgiure 7A), we noticed that microglial CD2AP haploinsufficiency had no effect on inflammatory factor expression in 5xFAD mice at this age (Supplemental Fgiure 7B). Although it was reported that amyloid burden and gliosis begin at 2 months of age in 5xFAD mice [54], we did not detect Aβ plaques (Supplemental Fig. 7C) and microgliosis (Supplemental Fig. 7D) in our studied 5xFAD mice at this age. Different facility maintenance conditions which can alter things like the microbiome may affect the pathology occurrence time. In addition, our studied 5xFAD mice were offspring from those crossed with *Cd2ap*^*fl/*+^;*Cx3cr1*-cre mice, and the offspring genetic background may also affect the pathology occurrence time. However, since we used littermates as controls, the alteration of pathology occurrence time will not affect the conclusion of our study.

### CD2AP deficiency in microglia reduces their responses to and phagocytosis of oligomerized Aβ42 without affecting Aβ plaques in pathological 5xFAD mice

**Fig. 4:**
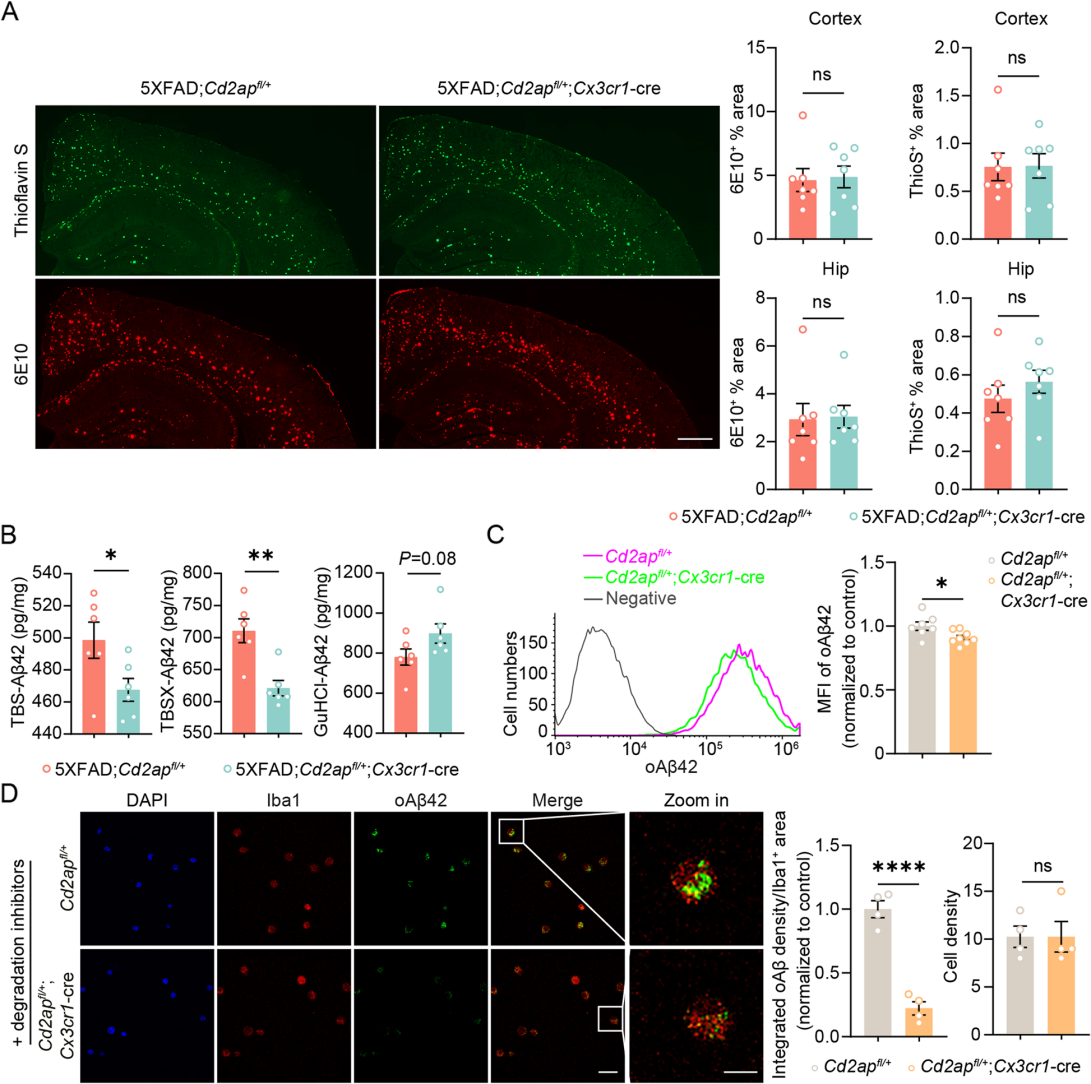
Microglial CD2AP haploinsufficiency reduces their phagocytosis of Aβ without affecting Aβ plaques in 5xFAD mice. **A** Representative images of Aβ plaques stained with Thioflavin S (ThioS, in green) and immunostained with an antibody against Aβ (6E10, in red) in the brain of 7-month-old 5xFAD;*Cd2ap*^*fl/*+^ and 5xFAD;*Cd2ap*^*fl/*+^;*Cx3cr1*-cre mice. Scale bars: 500 μm. Percentages of 6E10-immunostained and ThioS-stained areas in the cortex and hippocampus (Hip) were quantified for comparison. *n* = 7 mice per genotype. Unpaired Student’s* t* test. **B** Aβ42 levels in TBS-extractions, TBSX-extractions, and GuHCl-extractions of the hippocampal tissues from 7-month-old 5xFAD;*Cd2ap*^*fl/*+^ and 5xFAD;*Cd2ap*^*fl/*+^;*Cx3cr1*-cre mice were analyzed by ELISA. *n* = 5 mice per genotype. Unpaired Student’s* t* test. **C** Primary microglia from *Cd2ap*^*fl/*+^ and *Cd2ap*^*fl/*+^;*Cx3cr1*-cre mice were treated with TAMRA-oAβ and analyzed by flow cytometry. Untreated WT microglia were used as a negative control. The Mean fluorescence intensity (MFI) of TAMRA-oAβ^+^ primary microglia was used to indicate the ability of TAMRA-oAβ uptake and compared between different genotype groups. *Cd2ap*^*fl/*+^ group, *n* = 7; *Cd2ap*^*fl/*+^;*Cx3cr1*-cre group, *n* = 8. Unpaired Student’s* t* test. **D** Representative images and analysis of FAM-oAβ (in green) engulfed by Iba1^+^ microglia (in red) pretreated with MG132 and chloroquine. The Nuclei were stained with DAPI (in blue). The fluorescence intensity of FAM-oAβ in Iba1^+^ microglia and cell density were quantified for comparison. Scale bars for regular and zoom in images are 50 μm and 5 μm, respectively. *n* = 4. An average of data from approximately 12 cells in each replicate was indicated. Unpaired Student’s* t* test. Data are presented as mean ± SEM. *P < 0.05; **P < 0.01; ****P < 0.0001; ns, not significant

We used both Thioflavin S and the Aβ antibody 6E10 to label Aβ plaques in 7-month-old 5xFAD mice. The results showed that haploinsufficiency of CD2AP in microglia did not affect the area (Fig. 4A) and the average size (Supplemental Fig. 8A and B) of Aβ plaques, nor the numbers of Aβ plaques with different sizes (Supplemental Fig. 8A and B). We also measured Aβ compaction by calculating the ratio of Thioflavin S-positive plaque area over 6E10-positive plaque area [50] (Supplemental Fig. 8C-E) and found that neither the ratio of the total plaque area (Supplemental Fig. 8D) nor the ratio of the individual plaque area (Supplemental Fig. 8E) were altered upon CD2AP haploinsufficiency, suggesting that CD2AP haploinsufficiency has no effect on Aβ compaction. We further quantified hippocampal Aβ42 levels by ELISA and found that microglial CD2AP haploinsufficiency significantly reduced Aβ42 levels in TBS-soluble and TBSX-soluble fractions but not in the GuHCl-soluble fraction in 7-month-old 5xFAD mice (Fig. 4B). However on the other hand, microglial CD2AP haploinsufficiency in 2-month-old 5xFAD mice significantly increased TBS-soluble Aβ42 but not TBSX-soluble Aβ42 levels (Supplemental Fig. 7E). We could not detect GuHCl-soluble Aβ42 fraction in 2-month-old 5xFAD mice (data not shown).

Subsequently, we acquired primary microglia from *Cd2ap*^*fl/*+^;*Cx3cr1*-cre mice and *Cd2ap*^*fl/fl*^;*Cx3cr1*-cre mice. CD2AP levels were reduced by ~ 50% in the former (Supplemental Fig. 3D) and nearly completely depleted in the latter (Supplemental Fig. 9A). When these primary microglia were incubated with TAMRA-labeled oligomerized Aβ42 (oAβ42) for 1 h, flow cytometry (Fig. 4C) and immunofluorescence (Supplemental Fig. 9C and Supplemental Fig. 10A) assays revealed that oAβ42 levels within CD2AP-deficient microglia were significantly less than those in control cells. When microglia were pre-treated with the proteasome inhibitor MG132 and the lysosome inhibitor chloroquine to inhibit protein degradation and then incubated with FAM-labeled oAβ42 for 1 h, microglia with CD2AP haploinsufficiency still contained significantly less FAM-labeled oAβ42 levels than controls after incubation (Fig. 4D). These results indicate that CD2AP deficiency reduces microglial uptake of oAβ42. Moreover, it seems that CD2AP affects oAβ42 uptake in a dose-dependent manner, as microglia with complete CD2AP depletion (reduced to 28% of control, Supplemental Fig. 9C) had decreased oAβ42 uptake ability compared to those with CD2AP haploinsufficiency (reduced to 43% of control, Supplemental Fig. 10A).

**Fig. 5:**
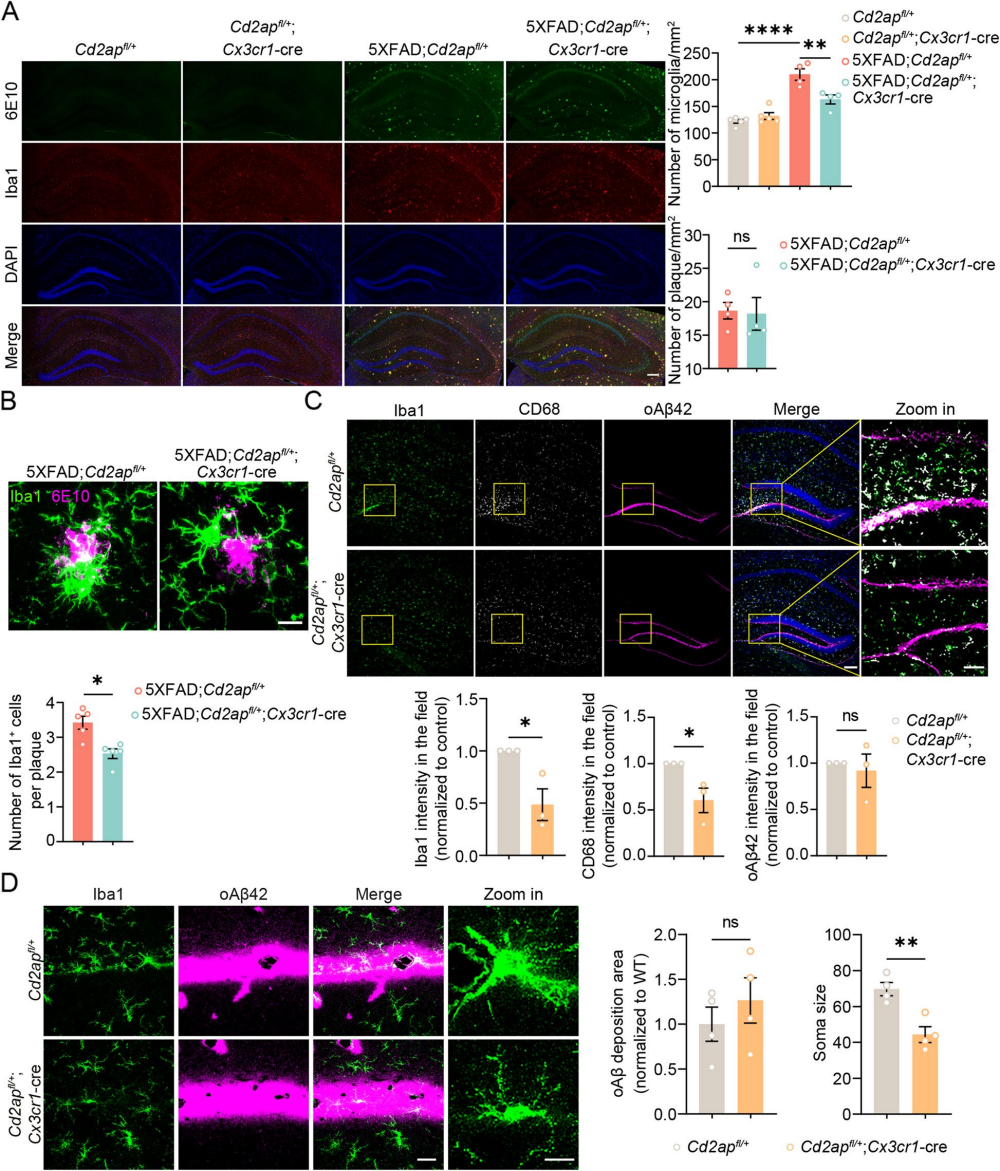
Microglial CD2AP haploinsufficiency suppresses Aβ-induced microglial activation. **A** Representative images showing hippocampal regions of 7-month-old mice with different genotypes immunostained with antibodies against Aβ (6E10, in green) and Iba1 (in red) and stained with DAPI (in blue). Scale bar: 200 μm. Microglia numbers (1-way ANOVA followed by Tukey’s post hoc test) and Aβ plaque numbers (unpaired Student’s *t* test) were quantified for comparison. *n* = 4 or 5 mice per genotype. **B** Representative images showing hippocampal regions of 7-month-old mice with different genotypes immunostained with antibodies against Aβ (6E10, in magenta) and Iba1 (in green). Scale bar: 10 μm. The numbers of microglia surrounding Aβ plaques with 20 μm in diameter were quantified for comparison. *n* = 5 mice per genotype. An average of data from 5 plaques in each mouse was indicated. Unpaired Student’s *t* test. **C** Representative images of 2-month-old *Cd2ap*^*fl/*+^ and *Cd2ap*^*fl/*+^;*Cx3cr1*-cre mice showing hippocampal regions injected with TAMRA-oAβ (in magenta), immunostained with antibodies against Iba1 (in green) and CD68 (in white), and stained with DAPI (in blue). Scale bars for regular and zoom in images are 50 μm and 20 μm, respectively. Intensities of Iba1, CD68, and oAβ in zoom in images were quantified for comparison. *n* = 3 mice per genotype. Unpaired Student’s *t* test. **D** Representative images of 2-month-old *Cd2ap*^*fl/*+^ and *Cd2ap*^*fl/*+^;*Cx3cr1*-cre mice showing regions injected with TAMRA-oAβ (in magenta) and immunostained with an anti-Iba1 antibody (in green). Scale bar: 30 μm. The area with oAβ injection and the soma size of Iba1^+^ microglia were quantified for comparison. *n* = 4 mice per genotype. Unpaired Student’s *t* test. Data are presented as mean ± SEM. *P < 0.05; **P < 0.01; ****P < 0.0001; ns, not significant

It is well-known that Aβ deposition can induce gliosis [19]. We confirmed that microgliosis and astrogliosis were significantly increased in 7-month-old 5xFAD mice, and found that microglial CD2AP haploinsufficiency reversed microgliosis (Fig. 5A) but had no effect on astrogliosis (Supplemental Fig. 8F and G). Furthermore, the numbers of microglia surrounding Aβ plaques were significantly reduced (Fig. 5B). When we stereotactically injected TAMRA-labeled oAβ42 into the mouse hippocampus, there were much less microglia that accumulated around oAβ42 regions and much lower intensity of CD68 (a lysosome marker indicative of microglia activation) in *Cd2ap*^*fl/*+^;*Cx3cr1*-cre mice than in *Cd2ap*^*fl/*+^ mice (Fig. 5C). In addition, microglia with CD2AP haploinsufficiency exhibited reduced soma size in response to oAβ42 (Fig. 5D). Together, these findings suggest that CD2AP haploinsufficiency reduces microgliosis in response to Aβ.

On the other hand, we noticed that in the absence of Aβ, basal microglial numbers in mice with microglial CD2AP haploinsufficiency (Fig. 5A) and in mice with complete microglial CD2AP depletion (Supplemental Fig. 9B) were not altered when compared to respective controls. These results implicate that CD2AP regulates microgliosis in response to toxicity and its deficiency has no marked effect on microglial activity under physiological conditions.

### Microglial CD2AP deficiency attenuates synapse loss in 5xFAD mice

**Fig. 6:**
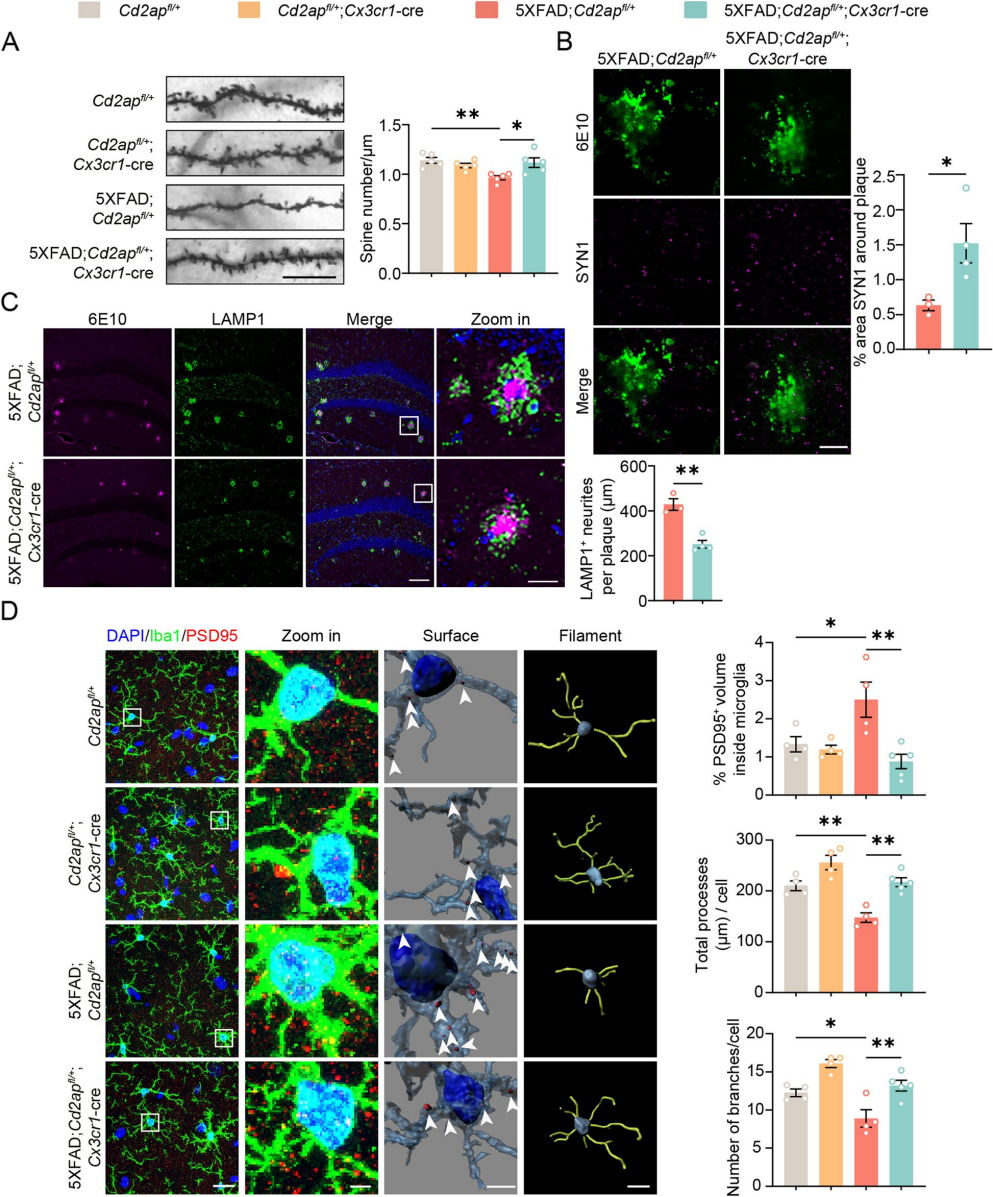
Microglial CD2AP haploinsufficiency reverses synapse loss in 5xFAD mice. **A** Representative images of Golgi staining of the hippocampal CA1 neuronal spines from 7-month-old mice. Scale bar: 10 μm. Spine numbers were quantified for comparison. *n* = 5 mice per genotype. An average of data from approximately 8 neurites in each mouse was indicated. 1-way ANOVA followed by Tukey’s post hoc test. **B** Representative images of the hippocampus of 7-month-old mice immunostained with antibodies against synapsin 1 (SYN1, in magenta) and Aβ (6E10, in green). Scale bar: 5 μm. The percentages of SYN1 area were quantified for comparison. *n* = 3 or 4 mice per genotype. An average of data from 4 plaques in each mouse was indicated. Unpaired Student’s *t* test. **C** Representative images of the hippocampus of 7-month-old mice immunostained with the 6E10 antibody (in magenta) and an anti-LAMP1 antibody (in green) and stained with DAPI (in blue). Scale bars for regular and zoom images are 100 μm and 20 μm, respectively. Aβ plaque–associated LAMP1-positive dystrophic neurites were quantified for comparison. *n* = 4 or 5 mice per genotype. An average of data from approximately 6 cells in each mouse was indicated. Unpaired Student’s* t* test. **D** Representative images and 3D surface and filament rendering of Iba1^+^ microglia (in green) containing PSD95^+^ puncta (in red) and the nuclei (in blue) in the hippocampus of 7-month-old mice with different genotypes. Each 2D image is generated from superimposing a series of 15 pictures with 1 μm step size in a z-stack direction to maximally reveal microglial branches. White arrows indicate PSD95^+^ puncta in microglia. Scale bars for regular, zoom in, 3D surface, and filament rendering images are 20 μm, 5 μm, 4 μm, and 10 μm, respectively. The percentages of PSD95^+^ puncta volume entirely within the microglia volume but outside the nucleus region, and total processes and branch numbers of microglia were quantified for comparison. *n* = 4 or 5 mice per genotype. An average of data from approximately 12 cells in each replicate was indicated. 1-way ANOVA followed by Tukey’s post hoc test. Data are presented as mean ± SEM. *P < 0.05; **P < 0.01; ns, not significant

To explore the effect of microglial CD2AP deficiency on neurons, we studied spine numbers by Golgi staining. The results showed that 7-month-old 5xFAD mice had a significant loss of spine numbers in hippocampal neurons; and this was rescued by microglial CD2AP haploinsufficiency (Fig. 6A). Immunostaining studies also revealed that the levels of a presynaptic membrane marker, synapsin 1 (SYN1) around Aβ plaques in 7-month-old 5xFAD mice were markedly increased by microglial CD2AP haploinsufficiency (Fig. 6B). We also found that levels of LAMP1-positive dystrophic neurites around Aβ plaques in 7-month-old 5xFAD mice were significantly reduced by microglial CD2AP haploinsufficiency (Fig. 6C). Through 3D reconstruction analysis of co-immunostained Iba1 and a postsynaptic marker, PSD95 in vivo, we further found significantly increased PSD95^+^ puncta amounts within Iba1^+^ microglia in 7-month-old 5xFAD mice; and this was reversed by microglial CD2AP haploinsufficiency (Fig. 6D). While PSD95^+^ puncta amounts within Iba1^+^ microglia in 2-month-old 5xFAD mice were not significantly altered, neither were they affected by microglial CD2AP haploinsufficiency at this age (Supplemental Fig. 7F). Microglia use their ramified branches to survey brain microenvironments under physiological conditions. Once detecting a pathological stimulus, microglia are activated and retract their branches to adopt a less complex structure [62]. We also found that the numbers and total length of microglial branches were reduced in 7-month-old 5xFAD mice and these changes were reversed by microglial CD2AP haploinsufficiency (Fig. 6D). Together, these results indicate that microglial CD2AP haploinsufficiency attenuates synapse loss in pathological 5xFAD mice probably through interfering with microglial uptake of synapses. Indeed, both immunofluorescence (Supplemental Fig. 10B and E) and flow cytometry (Supplemental Fig. 10C and D) experiments showed that synaptosomes (Supplemental Fig. 10B and C) and pHrodo green *E. coli* bioparticles (Supplemental Fig. 10D and E) were uptaken much less by microglia with CD2AP haploinsufficiency than by controls, with an uptake reduction of 76% of control for synaptosomes (Supplemental Fig. 10B) and 49% for bioparticles (Supplemental Fig. 10E). Moreover, we found that microglia with complete CD2AP depletion exhibited further decreased uptake abilities of synaptosomes (reduced to 20% of control, Supplemental Fig. 9D) and bioparticles (reduced to 36% of control, Supplemental Fig. 9E) when compared to microglia with CD2AP haploinsufficiency.

### Microglial CD2AP haploinsufficiency reverses elevated C1q expression in pathological 5xFAD mice

**Fig. 7:**
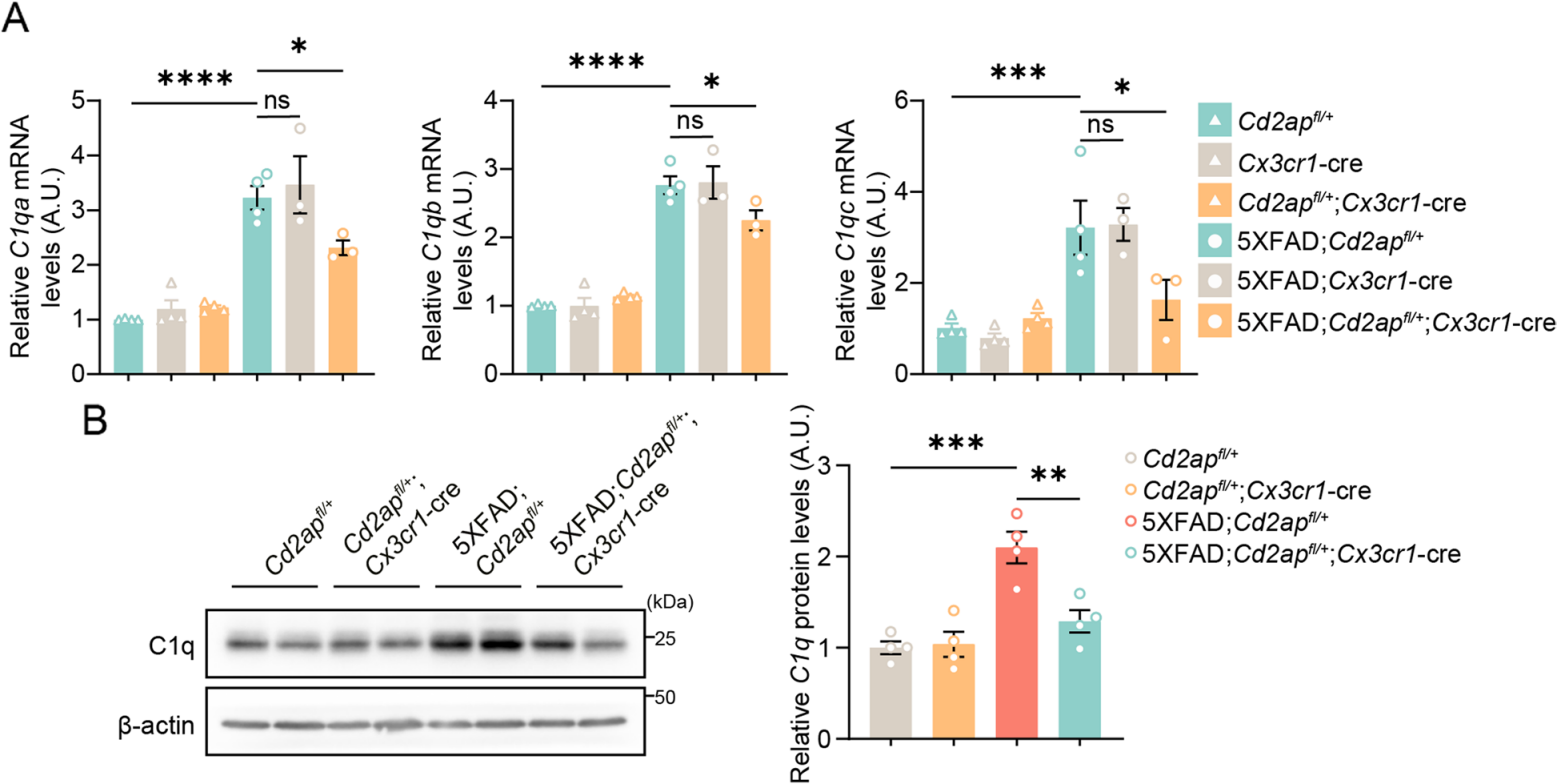
Microglial CD2AP haploinsufficiency reverses increased C1q expression in 5xFAD mice. **A** mRNA levels of *C1qa, C1qb, and C1qc* relative to those of *Gapdh* in the hippocampus of 7-month-old *Cd2ap*^*fl/*+^ (*n* = 4), *Cx3cr1*-cre (*n* = 4), *Cd2ap*^*fl/*+^;*Cx3cr1*-cre (*n* = 4), 5xFAD;*Cd2ap*^*fl/*+^ (*n* = 4), 5xFAD; *Cx3cr1*-cre (*n* = 3), and 5xFAD;*Cd2ap*^*fl/*+^;*Cx3cr1*-cre (*n* = 3) mice were determined for comparison. 1-way ANOVA followed by Dunnett’s post hoc test. **B** C1q protein levels in the hippocampus of 7-month-old mice with different genotypes were analyzed by western blot and quantified for comparison. *n* = 4 mice per genotype. 1-way ANOVA followed by Tukey’s post hoc test. Data are presented as mean ± SEM. *P < 0.05; **P < 0.01; ***P < 0.001; ****P < 0.0001; ns, not significant

The complement system plays an important role in microglia-mediated synaptic pruning. Recent studies have demonstrated that an altered complement system contributes to synapse loss in AD [20–22]. In our RNA-seq data, we found that the expression of some complement and related genes was altered in 7-month-old 5xFAD mice and reversed by microglial CD2AP haploinsufficiency (Fig. 3E). One of them, *C1qb*, encodes a subcomponent of the C1q protein. We then carried out qRT-PCR to study the expression of *C1qb, C1qa, and C1qc,* the latter two of which encode the other two subcomponents of C1q. We found that microglial CD2AP haploinsufficiency had no effect on their expression in a non-disease state but markedly reversed their increased expression in the 5xFAD background (Fig. 7A). Western blot also revealed significantly increased C1q protein levels in 7-month-old 5xFAD mice and this was reversed by microglial CD2AP haploinsufficiency (Fig. 7B). While C1q protein levels were neither increased nor altered by microglial CD2AP haploinsufficiency in 2-month-old 5xFAD mice (Supplemental Fig. 7G). By analyzing data from a previously reported AD proteomics study [53], we discovered that CD2AP levels were positively correlated with C1qa, C1qb, and C1qc levels in nearly all groups (control, asymptomatic AD patients, AD patients, and combined), except with C1qa and C1qb in AD groups (Supplemental Fig. 11A-C). Therefore, it is possible that decreased C1q levels underlie the protection against synapse loss in pathological 5xFAD mice by microglial CD2AP haploinsufficiency.

### CD2AP modulates CSF1R function

**Fig. 8:**
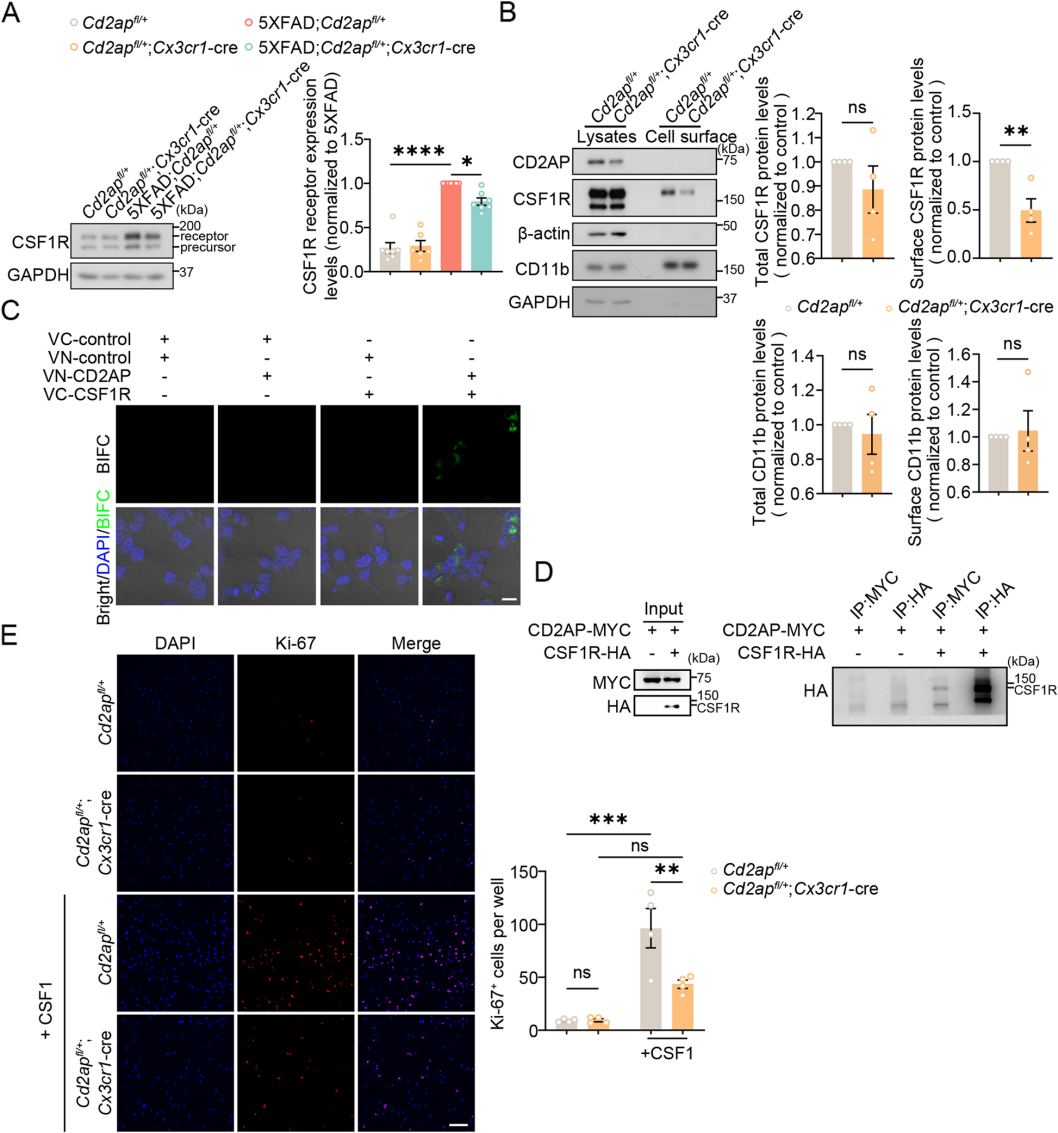
CD2AP interacts with CSF1R and modulates CSF1R function. **A** CSF1R protein levels in the hippocampus of 7-month-old mice with different genotypes were analyzed by western blot and quantified for comparison. *n* = 4 or 5 mice per genotype. 1-way ANOVA followed by Tukey’s post hoc test. **B** Total and cell surface levels of CSF1R and CD11b in cultured primary microglia derived from different mice were analyzed by western blot and quantified for comparison. *n* = 4 per genotype. Unpaired Student’s* t* test.** C** Representative confocal images of the BiFC assay showing green fluorescence specifically in cells co-transfected with VN-CD2AP and VC-CSF1R, indicative of an interaction between CD2AP and CSF1R. The nuclei were stained with DAPI (in blue). Scale bar: 20 μm. **D** HEK293T cells were transfected with CD2AP-MYC in the presence or absence of CSF1R-HA. Equal amounts of cell lysates were incubated with antibodies against MYC and HA for immunoprecipitation (IP) and then immunoblotted with an anti-HA antibody. **E** Primary microglia derived from *Cd2ap*^*fl/*+^ and *Cd2ap*^*fl/*+^;*Cx3cr1*-cre pups were treated with or without CSF1, and then immunostained with an antibody against Ki-67 (in red) to visualize microglial proliferation. The nuclei were stained with DAPI (in blue). The numbers of Ki-67^+^ cells were quantified for comparison. Scale bar: 100 μm. *n* = 4 independent experiments. An average of data from 4 fields of view taken in each experiment was indicated. 2-way ANOVA followed by Tukey’s post hoc test. Data are presented as mean ± SEM. *P < 0.05; **P < 0.01; ***P < 0.001; ****P < 0.0001; ns, not significant

The cell surface receptor CSF1R is an essential protein for microglia survival and function. Inhibition of CSF1R can deplete microglia and has been reported to reduce synapse loss in AD models [63–65]. Because our RNA-seq results showed that *Csf1r* expression was increased in 5xFAD mice and reversed by microglial CD2AP haploinsufficiency (Fig. 3E), we further studied the effect of CD2AP on CSF1R. We confirmed that CSF1R protein levels were increased in 5xFAD mice and reversed by microglial CD2AP haploinsufficiency (Fig. 8A). Although CD2AP haploinsufficiency had no effect on total levels of CSF1R in cultured primary microglia, it significantly reduced CSF1R cell surface levels without affecting those of CD11b (Fig. 8B). In addition, both microglia with complete CD2AP depletion (Supplemental Fig. 9F) and with CD2AP haploinsufficiency (Supplemental Fig. 12A) exhibited decreased ERK1/2 phosphorylation, suggesting an impaired CSF1R signaling in CD2AP-deficient microglia.

CD2AP is known to bind to many cell surface proteins and by searching the Human Protein–Protein Interactions Prediction Database [66, 67], we noticed that CD2AP has a high possibility of interacting with CSF1R (likelihood score >  = 12.5) (Supplemental Fig. 12B). We then carried out a Bimolecular Fluorescence Complementation (BiFC) assay and verified the CD2AP-CSF1R interaction (Fig. 8C). Co-immunoprecipitation experiments also showed that an anti-MYC antibody immunoprecipitated CSF1R-HA in cells co-expressing CD2AP-MYC and CSF1R-HA (Fig. 8D) and that an anti-CSF1R antibody but not an anti-CD93 antibody immunoprecipitated endogenous CD2AP in mouse brain samples (Supplemental Fig. 12C and D), further suggesting the interaction between CD2AP and CSF1R.

We also treated microglia with CSF1 to stimulate CSF1R signaling and immunostained Ki-67 to study microglial proliferation. The results showed that microglial proliferation activity was significantly enhanced in control microglia but not in CD2AP-deficient microglia by CSF1 treatment (Fig. 8E).

CD2AP is known to regulate the cytoskeleton [23, 25, 26]. By staining the actin cytoskeleton of cultured primary microglia with phalloidin, we found that CD2AP-deficient microglia had rounder morphologies than controls (Supplemental Fig. 12E). This result is different from the data showing that in vivo CD2AP-deficient microglia had a trend of branch increase (though not significant, Fig. 6D). Because microglia isolated from the newborn rodent brain are not mature and when they are cultured in petri dish without the support of brain microenvironment, microglia exhibit amoeboid-like shape and are generally in much more reactive state than they are in vivo [68]. Therefore, it is possible that CD2AP regulates microglial morphology in a microenvironment- and cell activation status-dependent manner.

Previous studies have shown that microglial morphology could turn into long strips in response to CSF1 [69]. We confirmed this and further found that CD2AP deficiency compromised microglial morphology change in response to CSF1 (Supplemental Fig. 12E).

We also examined the expression of different C1q subcomponents in cultured primary microglia with CSF1R haploinsufficiency. As expected, the expression of *C1qa*, *C1qb*, and *C1qc* were all decreased by CSF1R deficiency (Supplemental Fig. 13A). Furthermore, we found that the morphologies of microglia with CSF1R haploinsufficiency were also rounder than controls (Supplemental Fig. 13B), resembling those found in microglia with CD2AP haploinsufficiency. These results provide indirect evidence to suggest that CSF1R reduction may be responsible for the attenuation of microgliosis and synapse phagocytosis in pathological 5xFAD mice with microglial CD2AP haploinsufficiency.

## Discussion

SNPs located near the *CD2AP* locus, especially rs9349407 that locates in *CD2AP* intron 1, have been associated with LOAD by multiple genetic analyses [39, 70]. However, whether and how disease-associated SNPs affect CD2AP expression are scarcely studied. In one study analyzing human brain gene expression data including 10 brain regions from neuropathologically normal individuals of European descent from Braineac dataset, UK Brain Expression Consortium (UKBEC) [71], researchers found that the minor allele of rs9349407 was associated with decreased CD2AP expression only in cerebellar cortex [72]. However, in another study showing that the SNP rs9473117 located upstream of *CD2AP* was associated with EOAD, the authors also analyzed the Braineac dataset but found that the minor allele of rs9473117 was associated with increased CD2AP expression in thalamus and cerebellar cortex [32]. Results from functional studies are also controversial. For example, some cell culture studies showed that CD2AP knockdown blocked APP degradation and promoted Aβ generation [35, 36], whereas some other studies found that CD2AP knockdown reduced cell surface levels of APP and Aβ release [40, 41]. Although loss of the *CD2AP* ortholog *cindr* promoted tau-induced neuronal loss and reduced synaptic strength and life span in flies [33, 34], haploinsufficiency of CD2AP in the APPPS1 AD model mice had no effect on Aβ deposition in the brain [41]. Moreover, most of previous studies focused on the function of CD2AP in neurons and endothelial cells [33, 35, 37]. Although CD2AP was initially identified in immune cells, its function in microglia was not studied to-date to our knowledge. Herein, we found that CD2AP was highly expressed in microglia compared to neurons and astrocytes in both human and mice. We further noticed that CD2AP expression was dramatically increased in the brain of AD patients and 5xFAD mice at pathological stages, especially in microglia since their elimination abolished the increase of CD2AP in 5xFAD mice. These results clearly suggest an important role of microglial CD2AP in the brain under both physiological and pathological conditions. We then generated 5xFAD mice with heterozygous knockout of *Cd2ap* specifically in microglia. Unexpectedly, we found that CD2AP haploinsufficiency in microglia significantly attenuated cognitive and synaptic deficits in 5xFAD mice, indicating that microglial CD2AP deficiency protects against rather than worsens Aβ-linked microglial reactivity and injury.

Aberrant microglial activities including microgliosis, neuroinflammation, formation of DAM, and excessive synapse phagocytosis have been found in AD patients and animal models [4, 17–19, 22]. Herein, we confirmed these phenomena in pathological 5xFAD mice and further demonstrated that microglial CD2AP deficiency reduced microgliosis, DAM formation, and synapse uptake in pathological 5xFAD mice. Although CD2AP deficiency had no effect on pro-inflammatory factor generation, it tended to turn microglia into a more homeostatic phenotype. Microglial CD2AP haploinsufficiency also reversed the increased expression of many genes participating in aberrant microglial activities in pathological 5xFAD mice. These results suggest that CD2AP deficiency decreases microglial reactivity in response to amyloid burden that leads to neurotoxicity. Indeed, we found that CD2AP-deficient microglia exhibited altered morphology and reduced uptake of beads, synaptosomes, and oAβ42 in vitro, as well as reduced uptake of synapses and oAβ42 in vivo.

Dysregulated microglia homeostasis plays a very complicated role in AD [73]. For example, both ApoE and one of its important microglial receptor TREM2 are crucial for microglial activation in AD [13, 74]. Deletions of TREM2 and ApoE lead to impaired microglial cell response to amyloid plaques [75–77]; and ApoE4 impairs microglial response in the presence of amyloid [78]. These findings implicate that decreased microglial activity contributes to AD progression. However, elimination of microglia using inhibitors to CSF1R that plays an essential role in microglial survival, proliferation, and function [19, 42–44], exerts protection in AD model mice with or without affecting amyloid plaque burden [64, 65]. Elimination of microglia also blocks neurodegeneration in tauopathy model mice [79]. Here we found that microglial CD2AP haploinsufficiency compromised microglial activity and exerted protection in 5xFAD mice, a pattern similar to that found in CSF1R inhibitor-treated mice. Consistent with previous findings showing that CSF1R expression was elevated in the brain of AD patients and mouse models [80, 81], we also noticed that CSF1R expression was increased in 5xFAD mice and this increase was reversed by microglial CD2AP haploinsufficiency, probably because CD2AP deficiency reduces microgliosis under pathological conditions. Therefore, we further studied the relationship between CD2AP and CSF1R. Our results showed that CD2AP interacted with CSF1R. Moreover, CD2AP deficiency resulted in decreased cell surface levels of CSF1R in microglia and reduced CSF1-induced morphological changes and proliferation of microglia. While CSF1R-deficient microglia exhibited morphological changes and decreased C1q gene expression resembling those found in CD2AP-deficient microglia. These findings implicate that CD2AP deficiency affects microglial functions through compromising CSF1R and its signaling pathway. Consistent with this notion, it has been suggested that reduced cell surface levels of CSF1R not only contribute to a decrease in the number of Aβ-induced microgliosis but also a decrease in synapse loss [64, 65]. Some recent studies have further shown that inhibition of the CSF1R signaling pathway can alleviate cognitive impairment in multiple mouse models of neurodegeneration [63–65, 82–84]. Nevertheless, to ascertain that CSF1R reduction is responsible for CD2AP deficiency-caused changes of microglial functions, convincing data would be to show that restoring CSF1R expression reverses altered phenotypes in CD2AP-deficient microglia. Unfortunately, we tried but were not able to express exogenous CSF1R in CD2AP-deficient microglia both in vitro and in vivo (data not shown). Further study with advanced techniques to effectively modulating microglial gene expression may help ascertain whether CSF1R mediates CD2AP functions in microglia.

Endolysosomal dysfunction has been noticed as an important pathological feature in AD and multiple endolysosomal genes have been associated with AD risk, such as *SORL1*, *BIN1*, and *CD2AP* [85, 86]. CD2AP contains many actin-binding and membrane protein-binding sites and thus can regulate the cytoskeleton and membrane protein trafficking [23–28]. CD2AP knockdown in cell cultures could trap APP in early endosomes to limit its degradation, whereas overexpression of CD2AP showed the opposite effects [35, 36]. Here we found that CD2AP interacted with CSF1R and CD2AP deficiency resulted in decreased cell surface levels of CSF1R, implicating that CD2AP regulates CSF1R endocytosis and trafficking in microglia. Moreover, the increased *Csf1r* gene expression in 5xFAD mice was reversed by microglial CD2AP haploinsufficiency, raising a possibility that CD2AP also genetically modulates CSF1R.

Cells with a DAM signature are distributed spatially specifically around Aβ plaques and increase in number with the progression of amyloidosis [18]. The DAM signature has received ongoing attention since its discovery in amyloid depositing mice and in AD [17]. DAM activation is initiated in a TREM2-independent manner and followed by the activation of a TREM2-dependent program. Many marker genes are elevated in DAM, such as *CLEC7A*, *CD9*, *CCL6*, and *TREM2* [17]. Although DAM were initially considered neuroprotective in the setting of amyloid deposition, some recent studies have revealed that reducing cells with the DAM signature may halt components of the neurodegeneration process in the presence of AD [57, 58]. We found that microglia with the DAM signature was increased in pathological 5xFAD mice and decreased with microglial CD2AP haploinsufficiency. One study showed that use of CSF1R inhibitors could inhibit microglial proliferation, impair the development of DAM, and alleviate synapse loss [44], providing an indirect evidence to support that CD2AP deficiency may modulate DAM formation also through CSF1R and its signaling pathway. Of note, we also studied but failed to determine whether or not CD2AP regulates TREM2, a crucial DAM gene, due to a lack of reliable mouse TREM2 antibody. Further study is required to ascertain the role of CD2AP in DAM.

On the other hand, the elevated expression of many other genes important for microglial function, such as C1q in pathological 5xFAD mice was also attenuated by CD2AP haploinsufficiency, implicating that these genes also mediate the protection exerted by CD2AP deficiency. Compared to counts of Aβ plaques, tau tangles, and neuronal loss, synapse loss is more strongly correlated with the degree of dementia [1–3]. Multiple lines of evidence indicate that complement-mediated microglial phagocytosis of synapses is a key factor for synapse loss in AD. For example, deposition of C1q precedes Aβ plaque formation and is more likely to be deposited in areas with high synapse loss in AD model mice [22]. While the absence of C1q protects against synapse loss [20, 22]. Knockdown or inhibition of complement C3, as well as its receptor C3aR can also provide neuroprotection and protect against synapse loss [21, 22, 87, 88]. Herein, we found that increased expression of the three C1q subcomponent genes (including *C1qa*, *C1qb*, and *C1qc*) in 5xFAD mice was reversed upon microglial CD2AP haploinsufficiency. By analyzing the dataset of a reported proteomic study of AD and control patients [53], we further found that CD2AP levels were positively correlated with C1q levels. Furthermore, the expression of C1q genes was decreased in CSF1R-deficient microglia. Therefore, the reduction of C1q is a possible mechanism underlying microglial CD2AP deficiency-mediated attenuation of synapse loss in 5xFAD mice. However, since C1q is highly and almost exclusively expressed by microglia in the mouse brain [55] and microglial numbers were decreased in 5xFAD mice upon microglial CD2AP haploinsufficiency, C1q reduction may be attributed to either an overall reduction in microglia numbers or a decreased C1q expression in microglia; and this deserves further investigation.

We did not observe that microglial CD2AP deficiency significantly affected synapses under non-disease conditions in vivo, perhaps because microglial uptake of synapses is minimal under physiological conditions in adult mice.

A compromise of microglial functions may impair microglia-mediated Aβ clearance. We also found that CD2AP-deficient microglia had reduced Aβ uptake, and one might expect that CD2AP deficiency results in increased Aβ levels under AD conditions. Haploinsufficiency of microglial CD2AP in 2-month-old 5xFAD mice indeed significantly increased TBS-soluble Aβ42 levels, though Aβ plaques and GuHCl-soluble Aβ42 fraction were not detectable in mice at this age. However, haploinsufficiency of microglial CD2AP had no effect on Aβ plaque burden but significantly reduced TBS-soluble and TBSX-soluble Aβ42 levels in 7-month-old 5xFAD mice. A previous study also found that complete loss of CD2AP resulted in a decreased Aβ42/Aβ40 ratio while haploinsufficiency of CD2AP had no effect on Aβ deposition in the brain of PS1APP mice [41]. Emerging evidence suggests that the timing of microglial activity is crucial for amyloid pathology: TREM2 deficiency was found to ameliorate amyloid pathology early but exacerbate it late in the APPPS1-21 AD model mice [89]. CSF1R inhibition starting prior to Aβ pathology onset could reduce plaque burden but not when plaque load already exists [64, 65]. Therefore, one possible explanation for our contrasting findings between different ages is that although CD2AP-deficient microglia uptake less Aβ at early stages, they have decreased proinflammatory response to Aβ and phagocytosis of functional synapses at later stages, so that neurons in these mice are healthier and have less Aβ release than control neurons at the time of our study. Additionally, the reduction of microglial activation upon CD2AP deficiency may also reduce microglia-mediated neurotoxic Aβ process and spread [90–92]. Nevertheless, herein we only studied the effects of CD2AP deficiency on Aβ pathology at two timepoints. Further study on mice at additional pathological stages, such as when mice just start to develop Aβ plaques and when mice exhibit very severe Aβ plaque pathologies, will in the future provide a more thorough elucidation on how CD2AP dynamically modulates Aβ pathologies.

In conclusion, our study demonstrates that microglial CD2AP deficiency protects against disease-like phenotypes in AD mice, revealing a novel role of CD2AP in regulating microglial functions and their responses to Aβ toxicity in AD. One limitation of this study is that due to the technical difficulty of modulating CSF1R levels in CD2AP-deficient microglia, we are unable to ascertain whether the reduction of CSF1R is indeed responsible for the altered functions and morphology of CD2AP-deficent microglia. Further elucidating the underlying mechanisms, as well as determining the functions of CD2AP in other brain cells shall provide a comprehensive view on the contribution of CD2AP alteration in AD and help determine whether CD2AP may become a therapeutic target for AD intervention.

## Supplementary Information

Supplementary Material 1.

Supplementary Material 2.

## Data Availability

The RNA-seq data have been deposited into CNGB Sequence Archive (CNSA) of China National GeneBank DataBase (CNGBdb) with the accession number CNP0004099. Other data are available from the corresponding author upon reasonable request.

## Abbreviations

Aβ: β-Amyloid
AD: Alzheimer’s disease
BiFC: Biomolecular fluorescence complementation
CD2AP: CD2-associated protein
CSF1R: Colony stimulating factor 1 receptor
CX3CR1: C-X3-C motif chemokine receptor 1
DAM: Disease-associated microglia
DMEM: Dulbecco’s modified Eagle’s medium
FBS: Fetal bovine serum
GWAS: Genome-wide association studies
GO BP: Gene ontology terms for the biological process
GO CC: Gene ontology terms for the cellular component
LOAD: Late-onset, sporadic AD
LTP: Long-term potentiation
SNPs: Single nucleotide polymorphisms
SYN1: Synapsin 1
