# Supplementary material for: Microglial CD2AP deficiency exerts protection in an Alzheimer’s disease model of amyloidosis
Source: Mol Neurodegener. 2024 Dec 18;19:95. doi: 10.1186/s13024-024-00789-7 (PMC11658232; doi:10.1186/s13024-024-00789-7)
Supplement: Supplementary file 1 — Supplementary Material 1. [file 13024_2024_789_MOESM1_ESM.pdf]

Supplemental Figures and legends

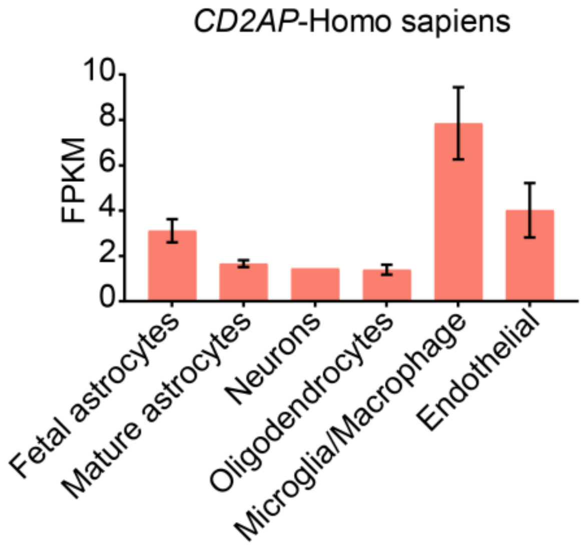

Supplemental Figure 1. CD2AP is highly expressed in human microglia. Expression of *CD2AP* in different human brain cell types.

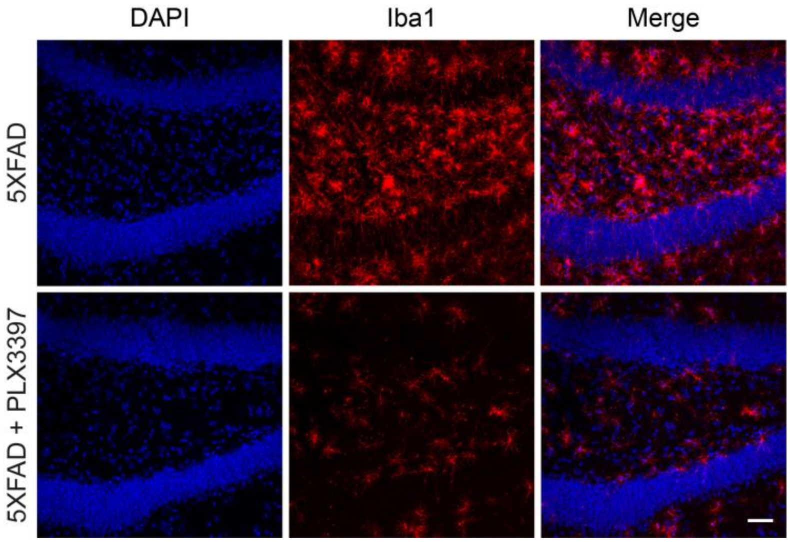

Supplemental Figure 2. Microglia depletion in 5xFAD mice. Representative images showing Iba1$^+$ microglia in the hippocampal region of 5xFAD mice fed with or without PLX3397. Scale bar: 50 μm.

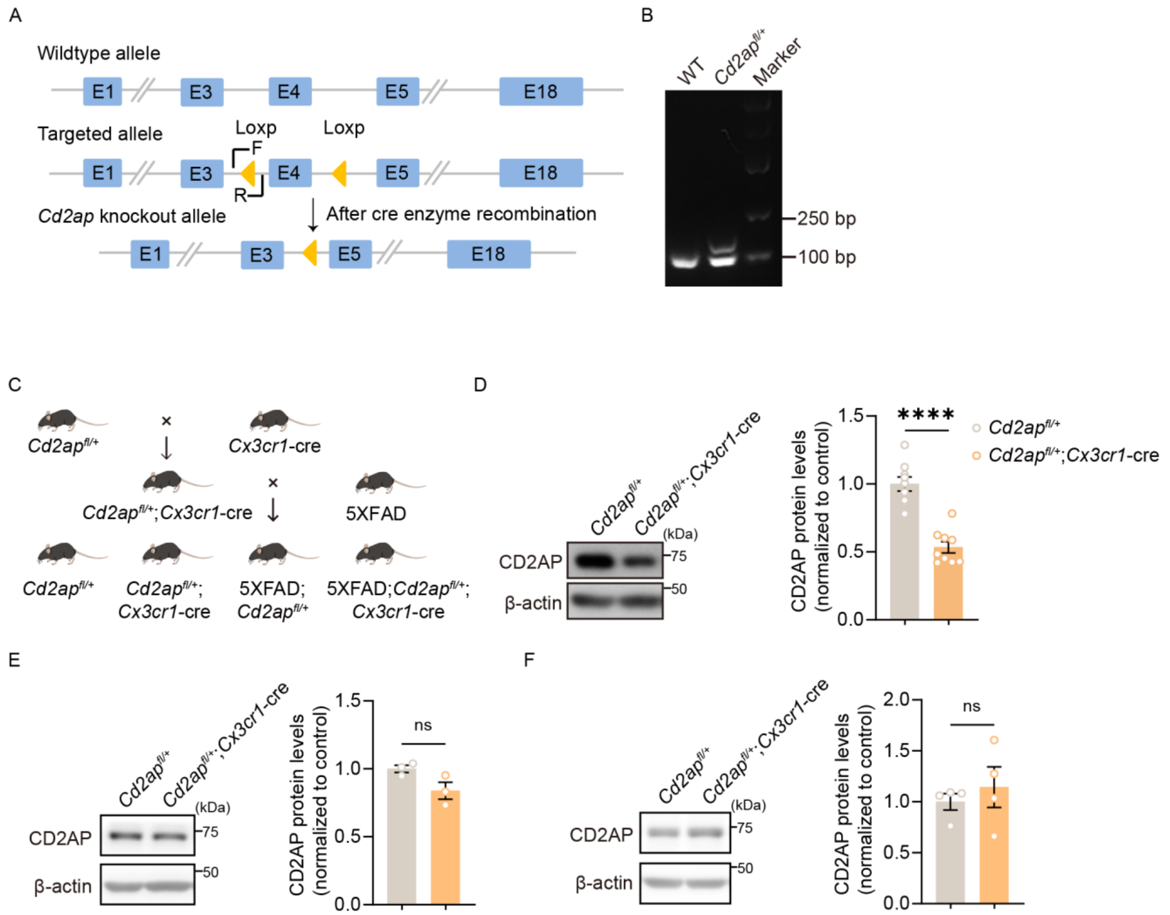

Supplemental Figure 3. Generation of microglial *Cd2ap*-deficient mice. (**A**) The strategy used for constructing *Cd2ap* conditional knockout mice. Yellow arrow, Loxp sites. F, binding site of the forward genotyping primer. R, binding site of the reverse genotyping primer. (**B**) Representative PCR results of *Cd2ap*$^{fl/+}$ mouse genotyping. (**C**) Schematic diagram for generating *Cd2ap*$^{fl/+}$, *Cd2ap*$^{fl/+}$;*Cx3cr1*-cre, 5xFAD;*Cd2ap*$^{fl/+}$, and 5xFAD;*Cd2ap*$^{fl/+}$;*Cx3cr1*-cre mice. (**D-F**) CD2AP protein levels in cultured primary microglia **(D)**, neurons **(E)**, and astrocytes **(F)** of *Cd2ap*$^{fl/+}$ and *Cd2ap*$^{fl/+}$;*Cx3cr1*-cre mice were analyzed by western blot and quantified for comparison. n = 9 for microglia, n = 3 for neurons, and n = 4 for astrocytes. Unpaired Student's *t* test. Data are presented as mean ± SEM. *****P* < 0.0001; ns, not significant.

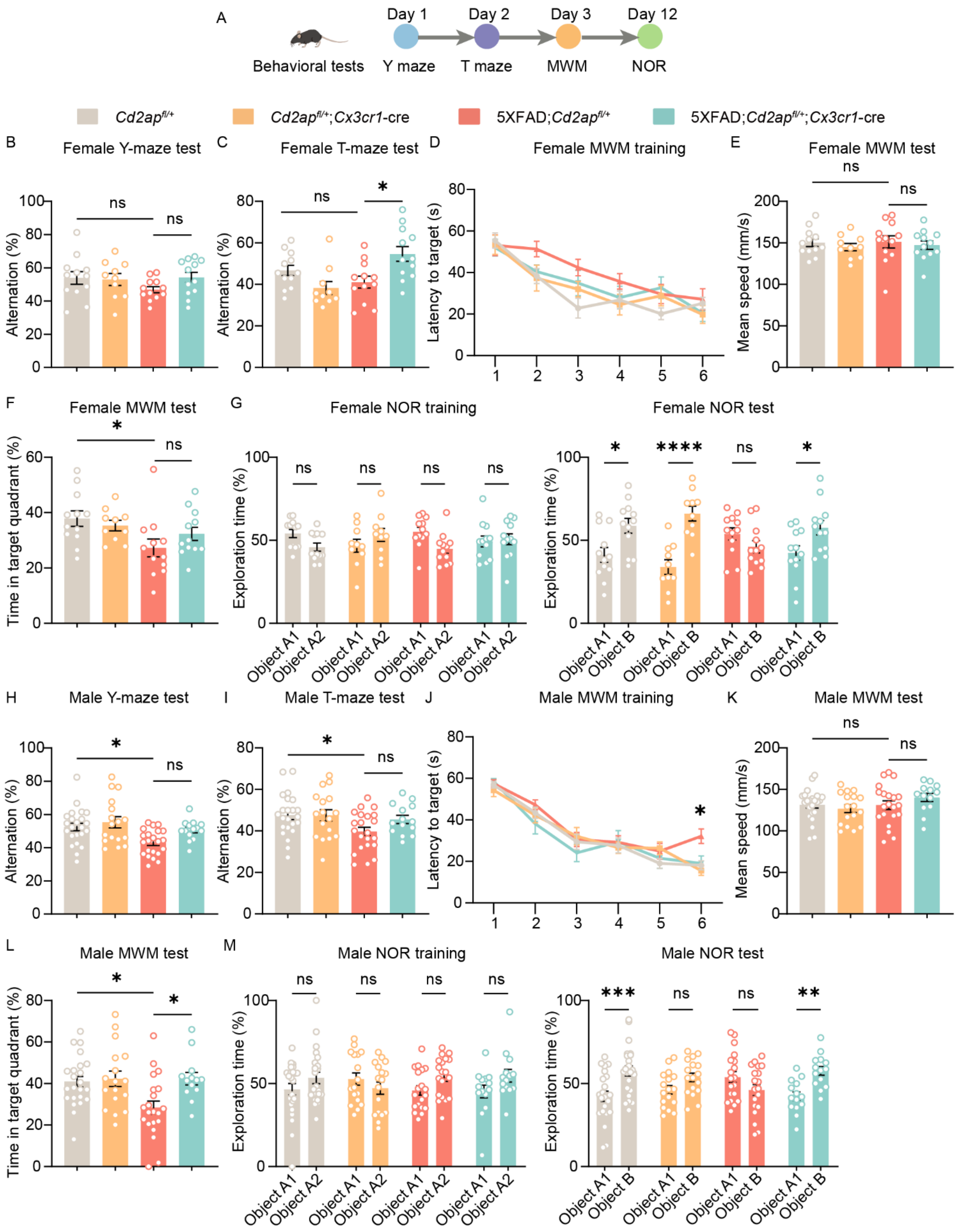

Supplemental Figure 4. Behavioral tests of male and female mice. (**A**) The timeline of behavioral tests. (**B,C**) In the Y-**(B)** and T-maze **(C)** tests, the percentages of spontaneous alternation were analyzed in 7-month-old *Cd2ap*$^{fl/+}$ (n = 12), *Cd2ap*$^{fl/+}$;*Cx3cr1*-cre (n = 10),

5xFAD;*Cd2ap*$^{fl/+}$ (n = 12), and 5xFAD;*Cd2ap*$^{fl/+}$;*Cx3cr1*-cre (n = 12) female mice. 1-way ANOVA followed by Tukey's post hoc test. (**D**-**F**) In the Morris water maze (MWM) test, latency to target during a six-day training **(D)**, and mean swim speed **(E)** and the percentages of time spent in the target quadrant **(F)** during the test on the 7th day were analyzed in 7-month-old female mice. Mouse numbers per group were the same as in **(B,C)**. 2-way ANOVA followed by Tukey's post hoc test for (**D**) and 1-way ANOVA followed by Tukey's post hoc test for (**E,F**). (**G**) In the novel object recognition (NOR) test, the percentages of exploration time to objects A1 and A2 during the training phase and to objects A1 and B during the test phase were analyzed in 7-month-old female mice. Mouse numbers per group was the same as in **(B,C)**. 2-way ANOVA followed by Tukey's post hoc test. (**H,I**) In the Y-**(H)** and T-maze **(I)** tests, the percentages of spontaneous alternation were analyzed in 7-month-old *Cd2ap*$^{fl/+}$ (n = 23), *Cd2ap*$^{fl/+}$;*Cx3cr1*-cre (n = 17), 5xFAD;*Cd2ap*$^{fl/+}$ (n = 22), and 5xFAD;*Cd2ap*$^{fl/+}$;*Cx3cr1*-cre (n = 14) male mice. 1-way ANOVA followed by Tukey's post hoc test. (**J**-**L**) In the MWM test, latency to target during the training **(J)**, and mean swim speed **(K)** and the percentages of time spent in the target quadrant **(L)** during the test were analyzed in 7-month-old *Cd2ap*$^{fl/+}$ (n = 23), *Cd2ap*$^{fl/+}$;*Cx3cr1*-cre (n = 17), 5xFAD;*Cd2ap*$^{fl/+}$ (n = 21, one mouse died after the T maze test), and 5xFAD;*Cd2ap*$^{fl/+}$;*Cx3cr1*-cre (n = 13, one mouse could not swim and was not tested for MWM) male mice. 2-way ANOVA followed by Tukey's post hoc test for (**J**) and 1-way ANOVA followed by Tukey's post hoc test for (**K,L**). (**M**) In the NOR test, the percentages of exploration time to objects A1 and A2 during the training phase and to

objects A1 and B during the test phase were analyzed in 7-month-old *Cd2ap*$^{fl/+}$ (n = 23), *Cd2ap*$^{fl/+}$;*Cx3cr1*-cre (n = 17), 5xFAD;*Cd2ap*$^{fl/+}$ (n = 19, 2 mice died after MWM), and 5xFAD;*Cd2ap*$^{fl/+}$;*Cx3cr1*-cre (n = 14, the mouse not tested for MWM was tested for NOR) male mice. 2-way ANOVA followed by Tukey's post hoc test. Data are presented as mean ± SEM. *$P < 0.05$; **$P < 0.01$; ***$P < 0.001$; ****$P < 0.0001$; ns, not significant.

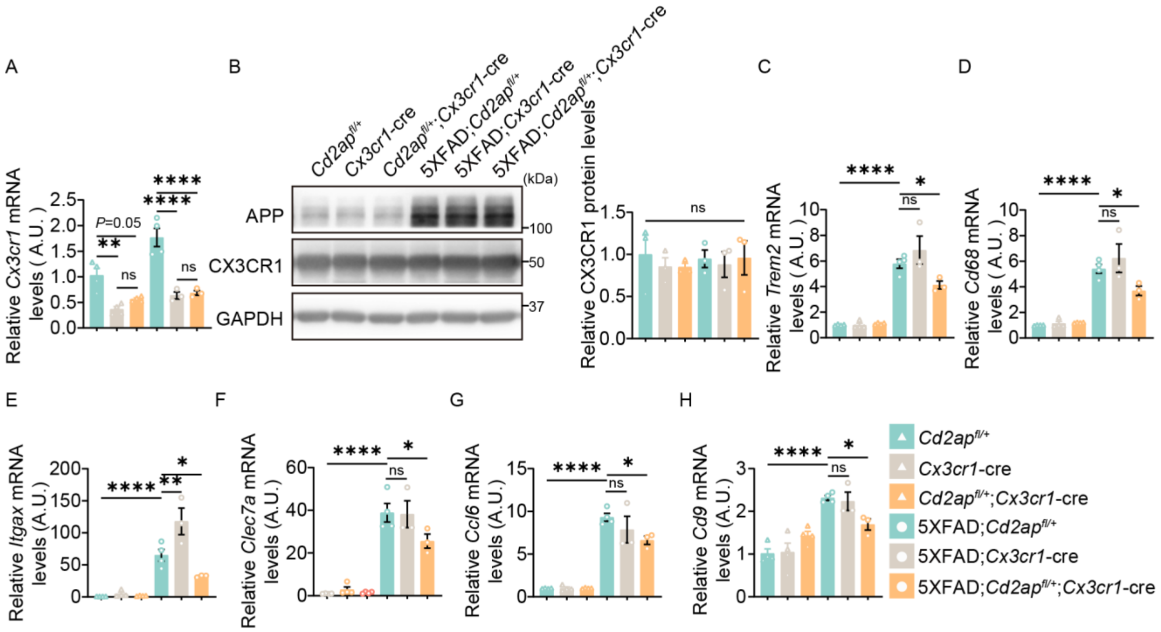

Supplemental Figure 5. Microglial CD2AP haploinsufficiency reverses DAM-associated gene expression in pathological 5xFAD mice. (**A**) *Cx3cr1* mRNA levels relative to those of *Gapdh* in the hippocampus of 7-month-old *Cd2ap*$^{fl/+}$ (n = 4), *Cx3cr1*-cre (n = 4), *Cd2ap*$^{fl/+}$;*Cx3cr1*-cre (n = 4), 5xFAD;*Cd2ap*$^{fl/+}$ (n = 4), 5xFAD;*Cx3cr1*-cre (n = 3) and 5xFAD;*Cd2ap*$^{fl/+}$;*Cx3cr1*-cre (n = 3) mice were determined for comparison. 1-way ANOVA followed by Tukey's post hoc test. (**B**) CX3CR1 protein levels in the hippocampus of 7-month-old mice with different genotypes were analyzed by western blot and quantified for comparison. n = 3 mice per genotype. 1-way ANOVA followed by Tukey's post hoc test. (**C**-**H**) mRNA levels of *Trem2* (**C**), *Cd68* (**D**), *Itgax* (**E**), *Clec7a* (**F**), *Ccl6* (**G**), and *Cd9* (**H**) relative to those of *Gapdh* in the hippocampus of 7-month-old mice with different genotypes were determined for comparison. Mouse numbers were the same as in (**A**). 1-way ANOVA followed by Dunnett's post hoc test. Data are presented as mean ± SEM. *$P < 0.05$; **$P < 0.01$; ****$P < 0.0001$; ns, not significant.

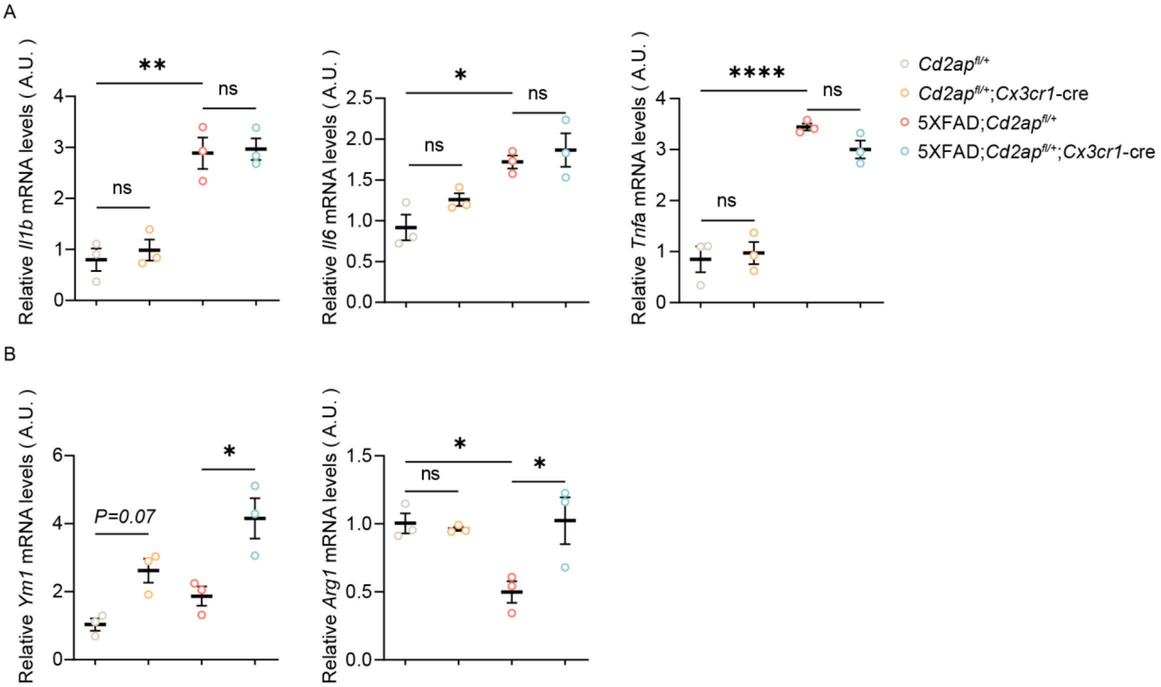

Supplemental Figure 6. Microglial CD2AP haploinsufficiency promotes anti-inflammatory factor expression in pathological 5xFAD mice. (**A**) mRNA levels of pro-inflammatory factors including *Il1b*, *Il6*, and *Tnfa* relative to those of *Gapdh* in the hippocampus of 7-month-old *Cd2ap*$^{fl/+}$, *Cd2ap*$^{fl/+}$;*Cx3cr1*-cre, 5xFAD;*Cd2ap*$^{fl/+}$, and 5xFAD;*Cd2ap*$^{fl/+}$;*Cx3cr1*-cre mice were determined for comparison. n = 3 mice per genotype. 1-way ANOVA followed by Tukey's post hoc test. (**B**) mRNA levels of anti-inflammatory factors including *Ym1* and *Arg1* relative to those of *Gapdh* in the hippocampus of 7-month-old mice with different genotypes were determined for comparison. n = 3 mice per genotype. 1-way ANOVA followed by Tukey's post hoc test. Data are presented as mean ± SEM. *$P < 0.05$; **$P < 0.01$; ****$P < 0.0001$; ns, not significant.

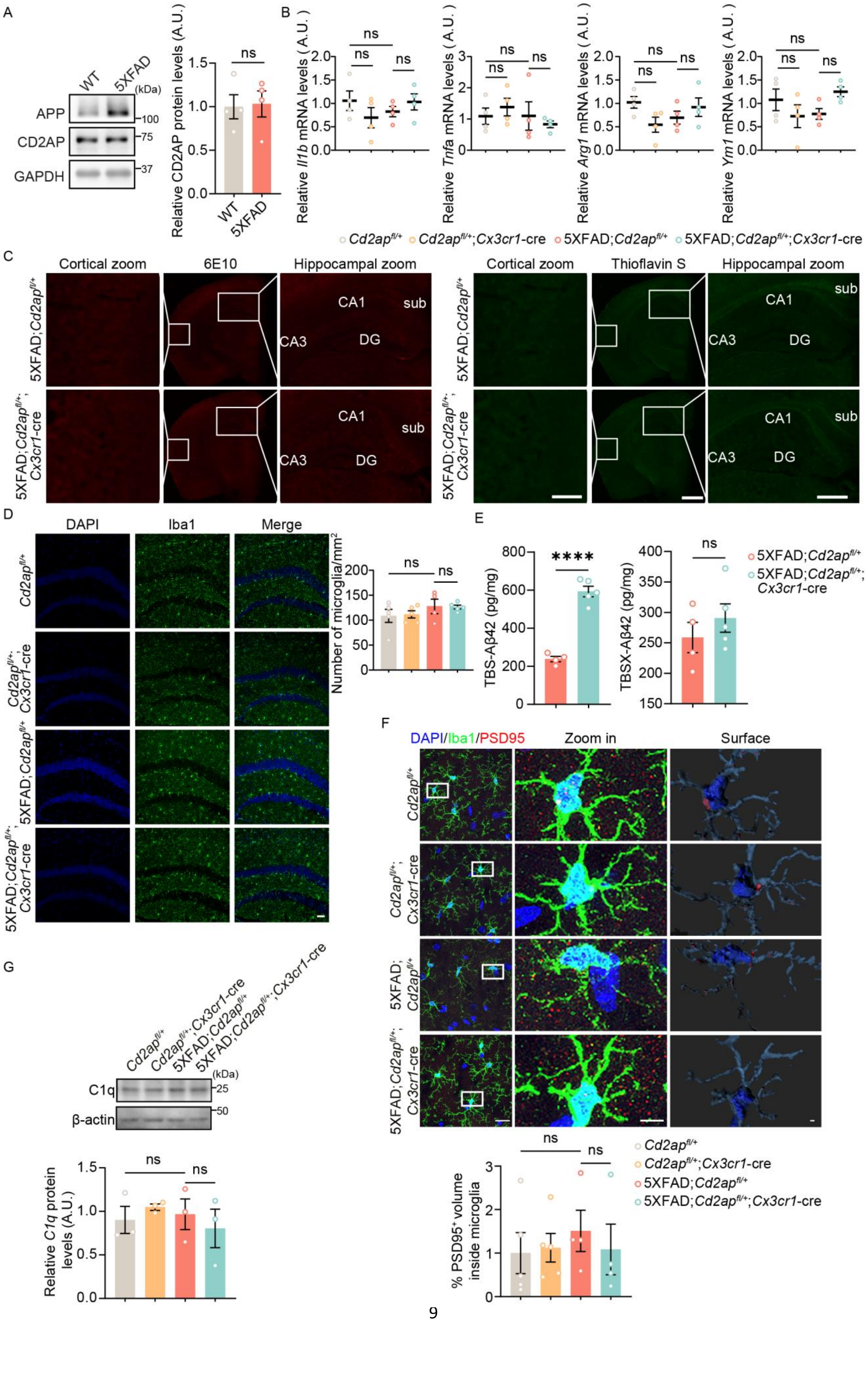
A
WT
5XFAD
(kDa)
APP
CD2AP
GAPDH
100
75
37
Relative CD2AP protein levels (A.U.)
ns
B
Relative Il1b mRNA levels (A.U.)
Relative Tnfa mRNA levels (A.U.)
Relative Arg1 mRNA levels (A.U.)
Relative Ym1 mRNA levels (A.U.)
Cd2apfl/+
Cd2apfl/+;Cx3cr1-cre
5XFAD;Cd2apfl/+
5XFAD;Cd2apfl/+;Cx3cr1-cre
C
Cortical zoom
6E10
Hippocampal zoom
Thioflavin S
CA1
CA3
DG
sub
D
DAPI
Iba1
Merge
Number of microglia/mm2
E
****
TBS-Aβ42 (pg/mg)
TBSX-Aβ42 (pg/mg)
F
DAPI/Iba1/PSD95
Zoom in
Surface
% PSD95+ volume inside microglia
G
C1q
β-actin
Relative C1q protein levels (A.U.)

Supplemental Figure 7. Microglial CD2AP haploinsufficiency has no effects on microglial activity in pre-pathological 5xFAD mice. (**A**) CD2AP protein levels in hippocampal tissues of WT and 5xFAD mice at 2 months of age were analyzed by western blot and quantified for comparison. n = 4 per group. Unpaired Student's *t* test. (**B**) mRNA levels of pro-inflammatory factors (including *Il1b* and *Tnfa*) and anti-inflammatory factors (including *Arg1* and *Ym1*) relative to those of *Gapdh* in the hippocampus of 2-month-old *Cd2ap*$^{fl/+}$, *Cd2ap*$^{fl/+}$;*Cx3cr1*-cre, 5xFAD;*Cd2ap*$^{fl/+}$, and 5xFAD;*Cd2ap*$^{fl/+}$;*Cx3cr1*-cre mice were determined for comparison. n = 4 mice per genotype. 1-way ANOVA followed by Tukey's post hoc test. (**C**) Representative images of amyloid plaques immunostained with the 6E10 antibody (in red, left panels) and stained with ThioS (in green, right panels) in the brain of 2-month-old 5xFAD;*Cd2ap*$^{fl/+}$ and 5xFAD;*Cd2ap*$^{fl/+}$;*Cx3cr1*-cre mice. Part of the cortical and hippocampal regions were zoomed in. Scale bars for regular, cortical zoom, and hippocampal zoom are 1 mm, 250 μm, and 500 μm, respectively. DG: dentate gyrus; sub: subiculum. (**D**) Representative images showing hippocampal regions of 2-month-old mice with different genotypes immunostained with an Iba1 antibody (in green) and stained with DAPI (in blue). Scale bar: 100 μm. Microglia numbers were quantified for comparison. n = 4 or 5 mice per genotype. 1-way ANOVA followed by Tukey's post hoc test. (**E**) Aβ42 levels in TBS-extractions and TBSX-extractions of the hippocampal tissues from 2-month-old 5xFAD;*Cd2ap*$^{fl/+}$ and 5xFAD;*Cd2ap*$^{fl/+}$;*Cx3cr1*-cre mice were analyzed by ELISA. n = 4 or 5 mice per genotype. Unpaired Student's *t* test. (**F**) Representative images and 3D surface rendering of Iba1^{+} microglia (in green) containing PSD95^{+} puncta (in red) and the

nuclei (in blue) in the hippocampus of 2-month-old mice with different genotypes. Each 2D image is generated from superposing a series of 13 pictures with 1 μm step size in a z-stack direction to maximally reveal microglial branches. Scale bars for regular, zoom in, and 3D surface rendering images are 20 μm, 5 μm, and 1 μm, respectively. The percentages of $PSD95^+$ puncta volume entirely within the microglia volume but outside the nucleus region were quantified for comparison. n = 4 or 5 mice per genotype. An average of data from approximately 4 cells in each replicate was indicated. 1-way ANOVA followed by Tukey's post hoc test. (**G**) C1q protein levels in the hippocampus of 2-month-old mice with different genotypes were analyzed by western blot and quantified for comparison. n = 3 mice per genotype. 1-way ANOVA followed by Tukey's post hoc test. Data are presented as mean ± SEM. ****$P < 0.0001$; ns, not significant.

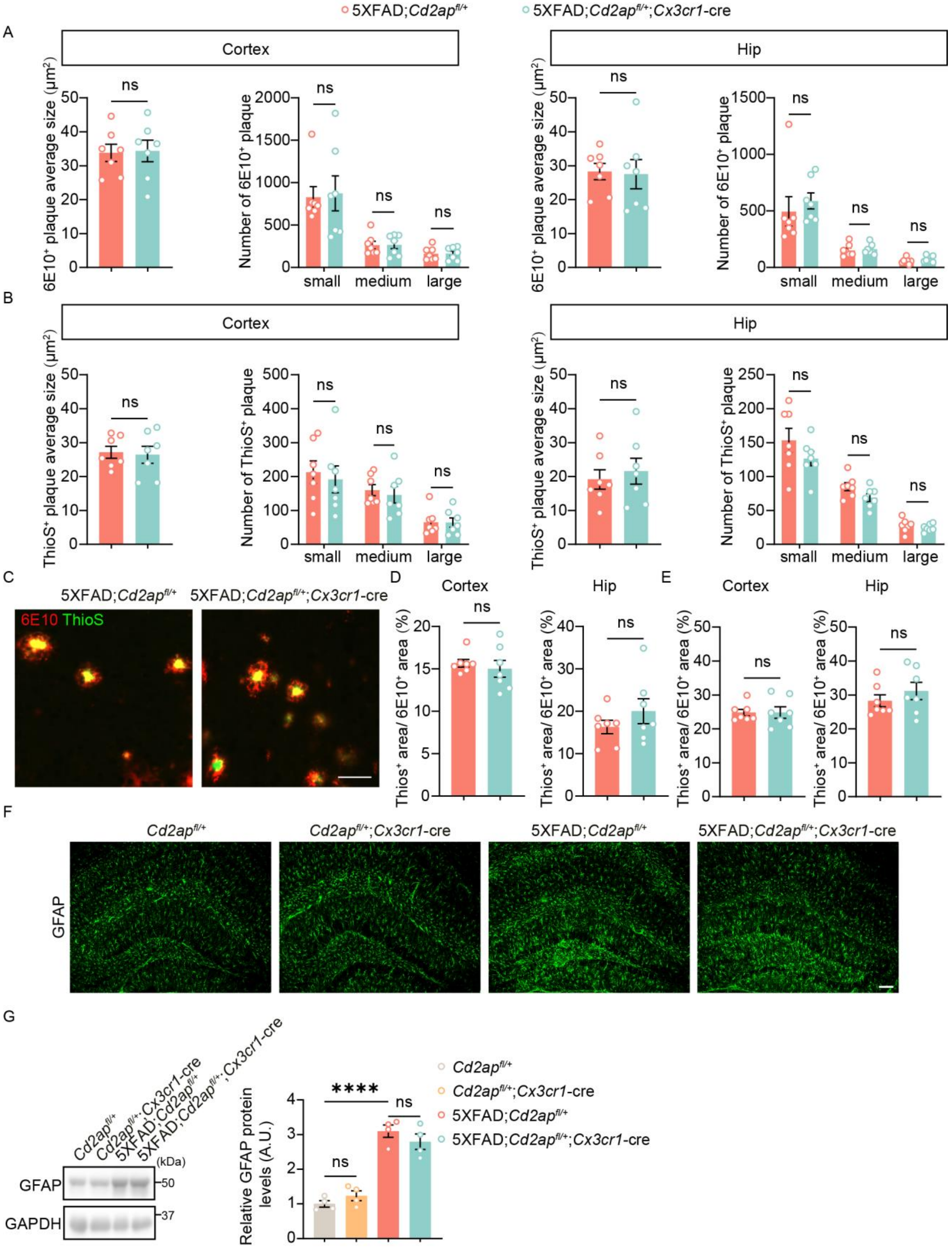

Supplemental Figure 8. Microglial CD2AP haploinsufficiency has no effect on amyloid compaction and astrogliosis in 5xFAD mice. (**A**) The average size of 6E10^{+}

plaques and the numbers of $6E10^+$ plaques with different sizes were determined for comparison in the cortex and hippocampus (Hip) of 7-month-old mice with different genotypes. Small size: 1-20 μm^2, medium size: 20-200 μm^2, large size: >200 μm^2. n = 7 mice per genotype. Unpaired Student's *t* test for average size comparison and 2-way ANOVA followed by Tukey's post hoc test for plaque number comparison. (**B**) The average size of $ThioS^+$ plaques and the numbers of $ThioS^+$ plaques with different sizes were determined for comparison in the cortex and hippocampus of 7-month-old mice with different genotypes. Small size: 1-20 μm^2, medium size: 20-200 μm^2, large size: >200 μm^2. n = 7 mice per genotype. Unpaired Student's *t* test for average size comparison and 2-way ANOVA followed by Tukey's post hoc test for plaque number comparison. (**C**) Representative images of Aβ plaques stained with ThioS (in green) and immunostained with 6E10 (in red) in the brain of 7-month-old 5xFAD;$Cd2ap^{fl/+}$ and 5xFAD;$Cd2ap^{fl/+}$;*Cx3cr1*-cre mice. Scale bars: 50 μm. (**D**) The ratio of total $ThioS^+$ plaque area over total $6E10^+$ plaque area was determined for comparison in the cortex and hippocampus of 7-month-old mice with different genotypes. n = 7 mice per genotype. Unpaired Student's *t* test. (**E**) The ratio of single $ThioS^+$ plaque area over its corresponding $6E10^+$ plaque area was determined comparison in the cortex and hippocampus of 7-month-old mice with different genotypes. An average of data from about 16 plaques in the hippocampus and an average of data from about 26 plaques in the cortex in each mouse are presented. n = 7 mice per genotype. Unpaired Student's *t* test. (**F**) Representative images showing $GFAP^+$ astrocytes in the hippocampal regions of 7-month-old mice with different

genotypes. Scale bar: 100 μm. (**G**) GFAP protein levels in the hippocampus of 7-month-old mice with different genotypes were analyzed by western blot and quantified for comparison. n = 4 mice per genotype. 1-way ANOVA followed by Tukey's post hoc test. Data are presented as mean ± SEM. $****P < 0.0001$; ns, not significant.

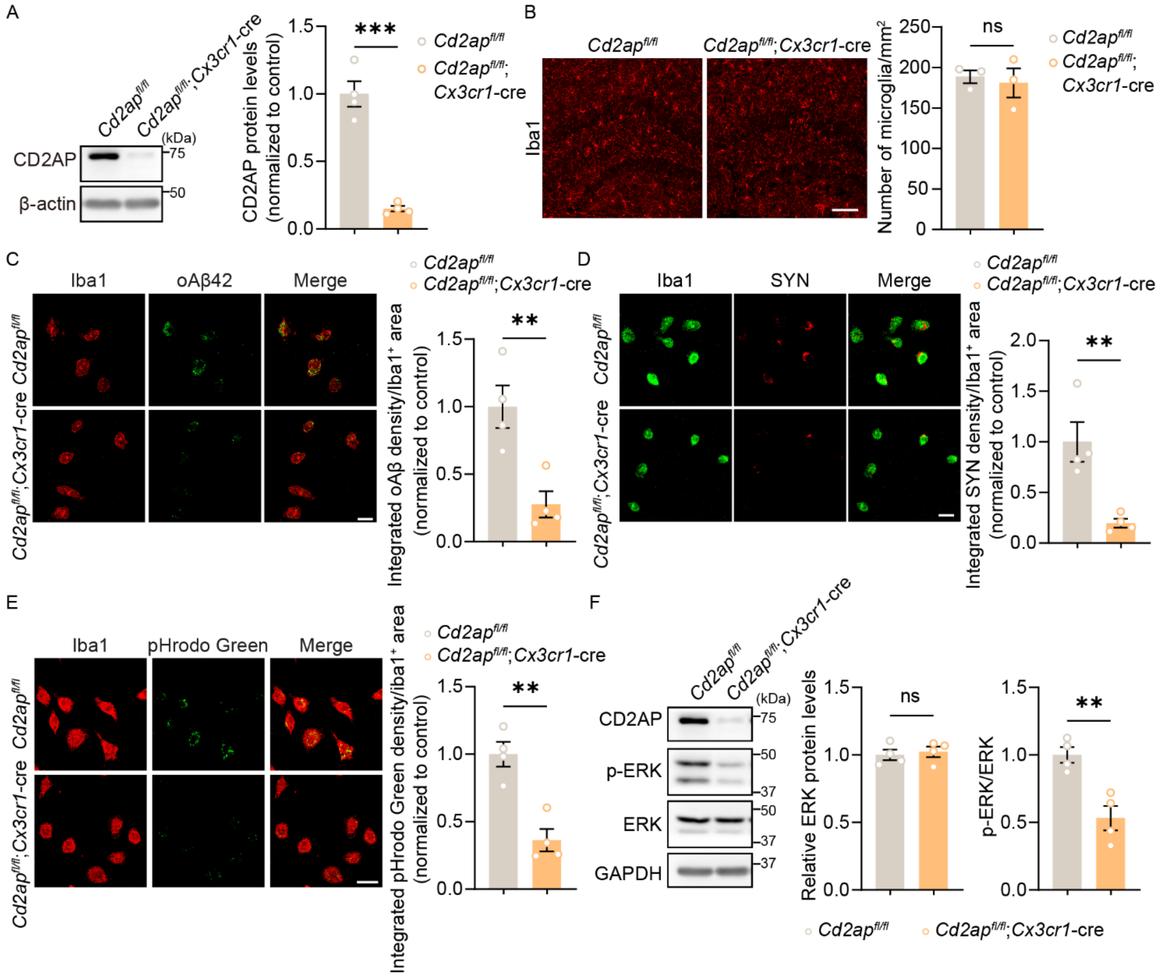

Supplemental Figure 9. Microglia with complete CD2AP depletion have decreased uptake ability and decreased ERK signaling. (**A**) CD2AP protein levels in cultured primary microglia of *Cd2ap^fl/fl^* and *Cd2ap^fl/fl^*;*Cx3cr1*-cre mice were analyzed by western blot and quantified for comparison. n = 4 mice per genotype. (**B**) Representative images showing Iba1$^+$ microglia in the hippocampal region of 2-month-old *Cd2ap^fl/fl^* and *Cd2ap^fl/fl^*;*Cx3cr1*-cre mice. Scale bar: 100 μm. Microglia numbers were quantified for comparison. n = 3 mice per genotype. (**C**) Representative images and analysis of FAM-oAβ (in green) engulfed by Iba1$^+$ primary microglia (in red) derived from *Cd2ap^fl/fl^* and *Cd2ap^fl/fl^*;*Cx3cr1*-cre mice. Scale bar: 20 μm. The densities of FAM-oAβ in Iba1$^+$ microglia

area were quantified for comparison. n = 4 mice per genotype. An average of data from approximately 18 cells in each replicate was indicated. (**D**) Representative images showing pHrodo red dye-labeled synaptosomes (SYN, in red) engulfed by Iba1$^+$ primary microglia (in green) derived from *Cd2ap*$^{fl/fl}$ and *Cd2ap*$^{fl/fl}$;*Cx3cr1*-cre mice. Scale bar: 20 μm. The densities of pHrodo red dye-labeled SYN in the Iba1$^+$ area were quantified for comparison. n = 4 mice per genotype. An average of data from approximately 15 cells in each replicate was indicated. (**E**) Representative images showing pHrodo green *E. coli* bioparticles (in green) engulfed by Iba1$^+$ primary microglia (in red) derived from *Cd2ap*$^{fl/fl}$ and *Cd2ap*$^{fl/fl}$;*Cx3cr1*-cre mice. Scale bar: 100 μm. The densities of pHrodo green *E. coli* bioparticles in Iba1$^+$ microglia area were quantified for comparison. n = 4 mice per genotype. An average of data from approximately 15 cells in each replicate was indicated. (**F**) ERK and phosphorylated ERK (p-ERK) proteins in cultured primary microglia derived from *Cd2ap*$^{fl/fl}$ and *Cd2ap*$^{fl/fl}$;*Cx3cr1*-cre mice were analyzed by western blot and quantified for comparison. n = 4 per genotype. Unpaired Student's *t* test. Data are presented as mean ± SEM and all statistical analyses were performed by unpaired Student's *t* test. $^{**}P < 0.01$; $^{***}P < 0.001$; ns, not significant.

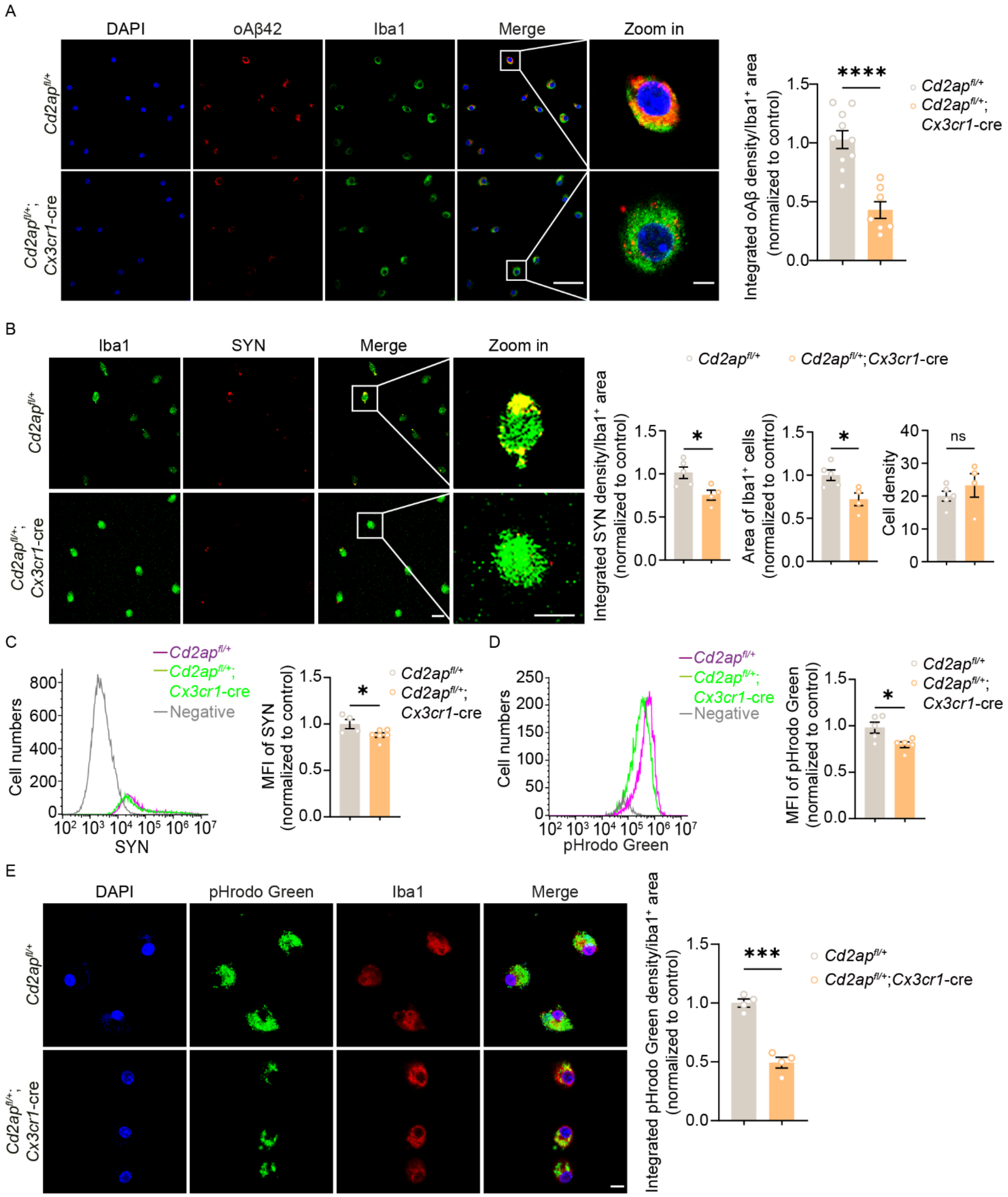

Supplemental Figure 10. Haploinsufficiency of CD2AP in microglia reduces microglial uptake. (**A**) Representative images and analysis of TAMRA-oAβ (in red) engulfed by Iba1$^+$ microglia (in green). The Nuclei were stained with DAPI (in blue). Scale bars for regular and zoom in images are 50 µm and 5 µm, respectively. *Cd2ap$^{fl/+}$* group, n

= 10; *Cd2ap*$^{fl/+}$;*Cx3cr1*-cre group, n = 7. An average of data from approximately 8 cells in each replicate was indicated. (**B**) Representative images showing pHrodo red dye-labeled synaptosomes (SYN, in red) engulfed by Iba1^{+} microglia (in green) derived from mice with different genotypes. Scale bars for regular and zoom in images are 20 μm and 10 μm, respectively. The densities of pHrodo red dye-labeled SYN in the Iba1^{+} area and the area of Iba1^{+} microglia were quantified for comparison. *Cd2ap*$^{fl/+}$ group, n = 5; *Cd2ap*$^{fl/+}$;*Cx3cr1*-cre group, n = 4. An average of data from approximately 20 cells in each replicate was indicated. (**C**) Primary microglia from *Cd2ap*$^{fl/+}$ and *Cd2ap*$^{fl/+}$;*Cx3cr1*-cre mice were treated with pHrodo red dye-labeled SYN and analyzed by flow cytometry. Untreated WT microglia were used as a negative control. The MFI of SYN^{+} cells in different genotype groups were compared. *Cd2ap*$^{fl/+}$ group, n = 4; *Cd2ap*$^{fl/+}$;*Cx3cr1*-cre group, n = 6. (**D**) Flow cytometry analysis of different microglia cells treated with pHrodo green *E. coli* bioparticles. The MFI of pHrodo green^{+} cells in different genotype groups were compared. n = 5 per genotype. (**E**) Representative images showing pHrodo green *E. coli* bioparticles (in green) engulfed by Iba1^{+} microglia (in red) derived from different mice. Scale bars: 10 μm. The density of pHrodo green *E. coli* bioparticles in Iba1^{+} microglia area were quantified for comparison. n = 4. An average of data from approximately 4 cells in each replicate was indicated. Data are presented as mean ± SEM and all statistical analyses were performed by unpaired Student's *t* test. *$P < 0.05$; ***$P < 0.001$; ****$P < 0.0001$; ns, not significant.

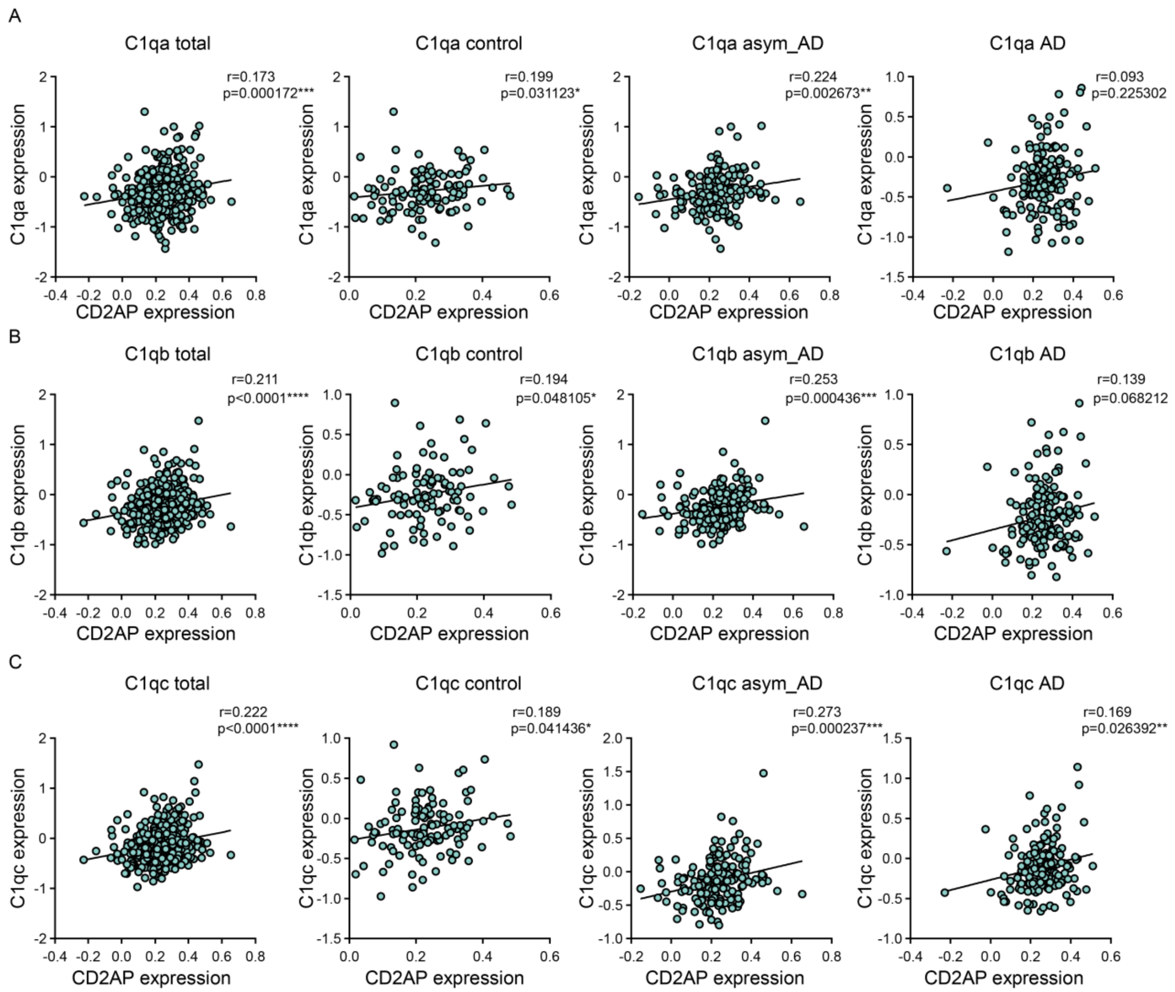

Supplemental Figure 11. CD2AP protein levels positively correlate with C1q protein levels in human brain. (**A-C**) The correlation of CD2AP protein levels with C1qa (A), C1qb (B), and C1qc (C) protein levels in the brain of total, control, asymptomatic AD (Asym_AD), and AD samples were studied using Spearman correlation analysis. Protein level data are from [51]. *$P < 0.05$; **$P < 0.01$; ***$P < 0.001$; ****$P < 0.0001$.

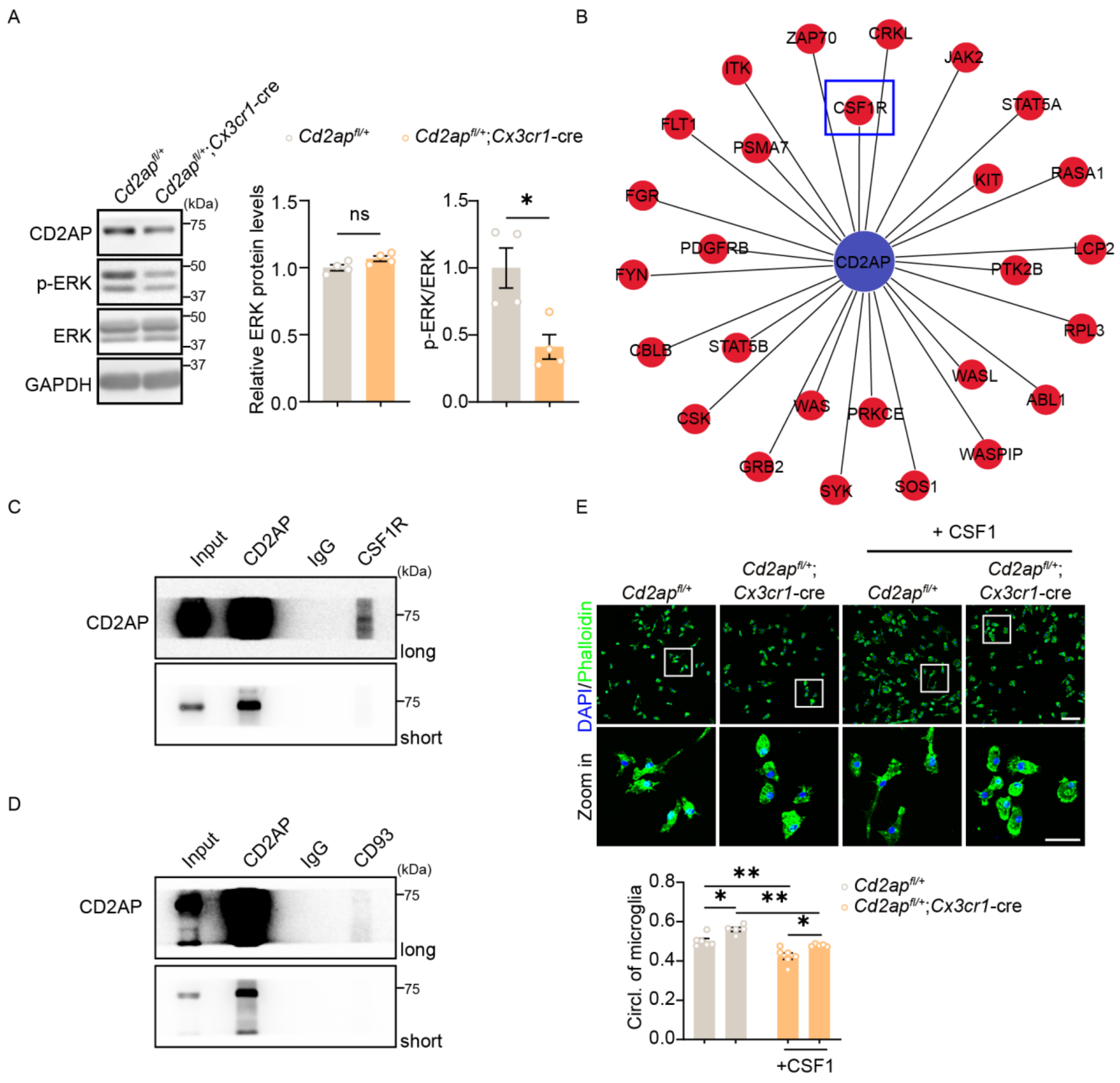

Supplemental Figure 12. CD2AP interacts with CSF1R and modulates CSF1R function. (**A**) ERK and phosphorylated ERK (p-ERK) proteins in cultured primary microglia derived from *Cd2ap*$^{fl/+}$ and *Cd2ap*$^{fl/+}$;*Cx3cr1*-cre pups were analyzed by western blot and quantified for comparison. Data are presented as mean ± SEM. n = 4 per genotype. Unpaired Student's *t* test. (**B**) Proteins predicted to interact with CD2AP in Human Protein-protein Interactions Prediction database (PIPs, likelihood score >=12.5). (**C**) Equal amounts of WT mouse brain lysates were incubated with IgG or antibodies against CD2AP

and CSF1R. Immunoprecipiated proteins were immunoblotted with the anti-CD2AP antibody. Both long and short expoures are shown. (**D**) Equal amounts of WT mouse brain lysates were incubated with IgG or antibodies against CD2AP and CD93. Immunoprecipiated proteins were immunoblotted with the anti-CD2AP antibody. Both long and short expoures are shown. (**E**) Different primary microglia were treated with or without CSF1, and then treated with green fluorescence-labeled phalloidin that specifically binds F-actin (in green) to visualize microglial cytoskeleton. The nuclei were stained with DAPI (in blue). Scale bars for regular and zoom in images are 100 μm and 30 μm, respectively. The circularities of microglia were quantified for comparison. n = 5. An average of data from approximately 200 cells in each replicate was indicated. 2-way ANOVA followed by Tukey's post hoc test. Data are presented as mean ± SEM. $*P < 0.05$; $**P < 0.01$; ns, not significant.

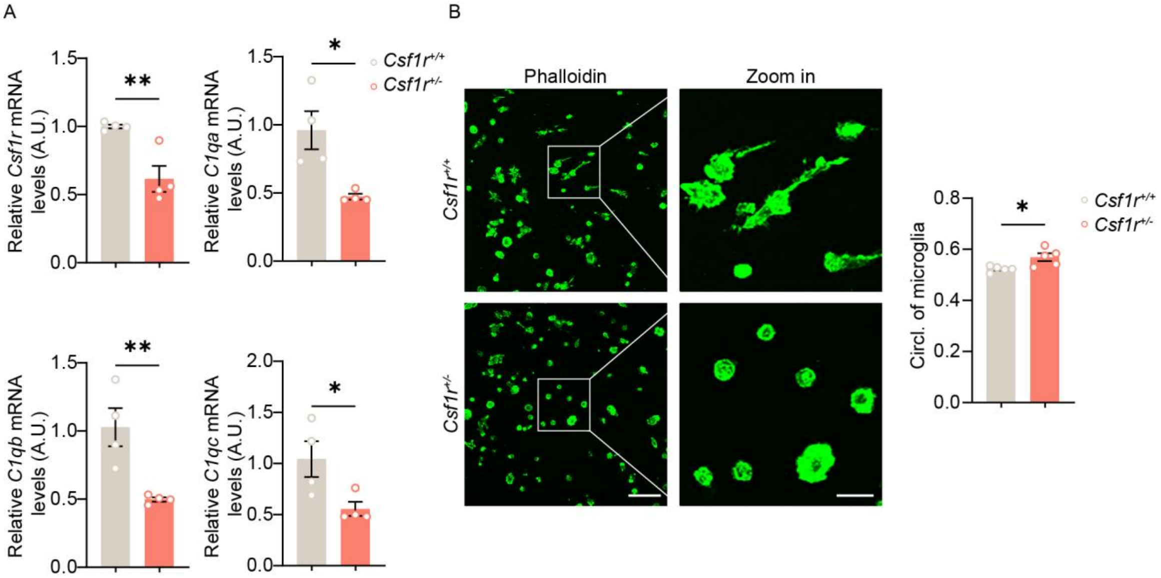

Supplemental Figure 13. CSF1R haploinsufficiency results in C1q expression reduction and microglial morphology change. (**A**) mRNA levels of *C1qa, C1qb, and C1qc* relative to those of *Gapdh* in cultured primary microglia from *Csf1r*$^{+/+}$ and *Csf1r*$^{+/-}$ mouse pups were determined for comparison. n = 4 per genotype. (**B**) Fluorescence-labeled phalloidin was used to stain F-actin (in green) for visualizing the cytoskeleton of cultured primary microglia of *Csf1r*$^{+/+}$ and *Csf1r*$^{+/-}$ mice. Scale bars for regular and zoom in images are 100 μm and 30 μm, respectively. The circularities of microglia were quantified for comparison. n = 5. An average of data from approximately 180 cells in each replicate was indicated. Data are presented as mean ± SEM and all statistical analyses were performed by unpaired Student's *t* test. *$P < 0.05$; **$P < 0.01$.
