# Supplementary material for: Microglial CD2AP deficiency exerts protection in an Alzheimer’s disease model of amyloidosis
Source: Mol Neurodegener. 2024 Dec 18;19:95. doi: 10.1186/s13024-024-00789-7 (PMC11658232; doi:10.1186/s13024-024-00789-7)
Supplement: Supplementary file 2 — Supplementary Material 2. [file 13024_2024_789_MOESM2_ESM.pdf]

Full unedited gel for Figure 1B

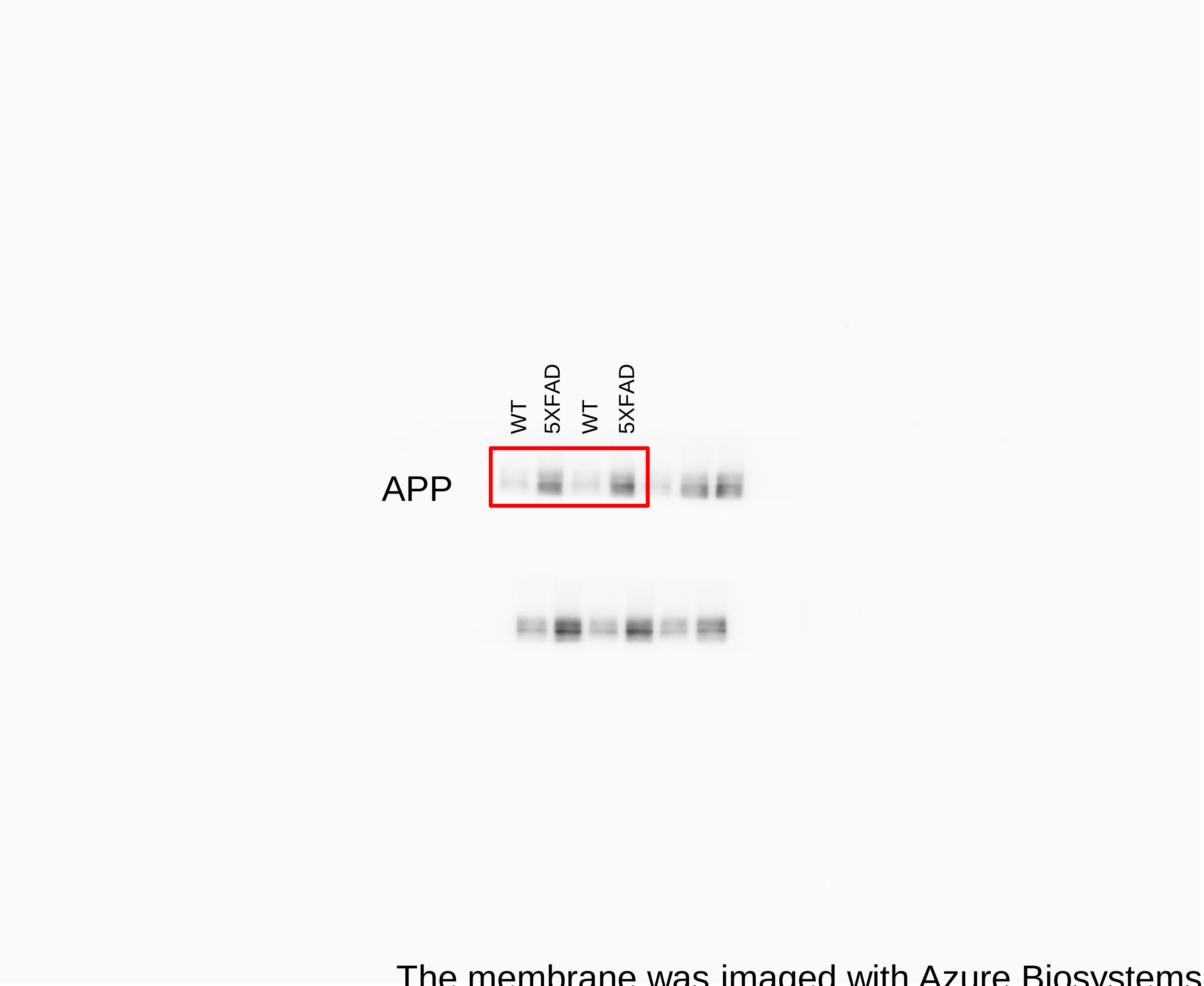

The membrane was imaged with Azure Biosystems 300

Full unedited gel for Figure 1B

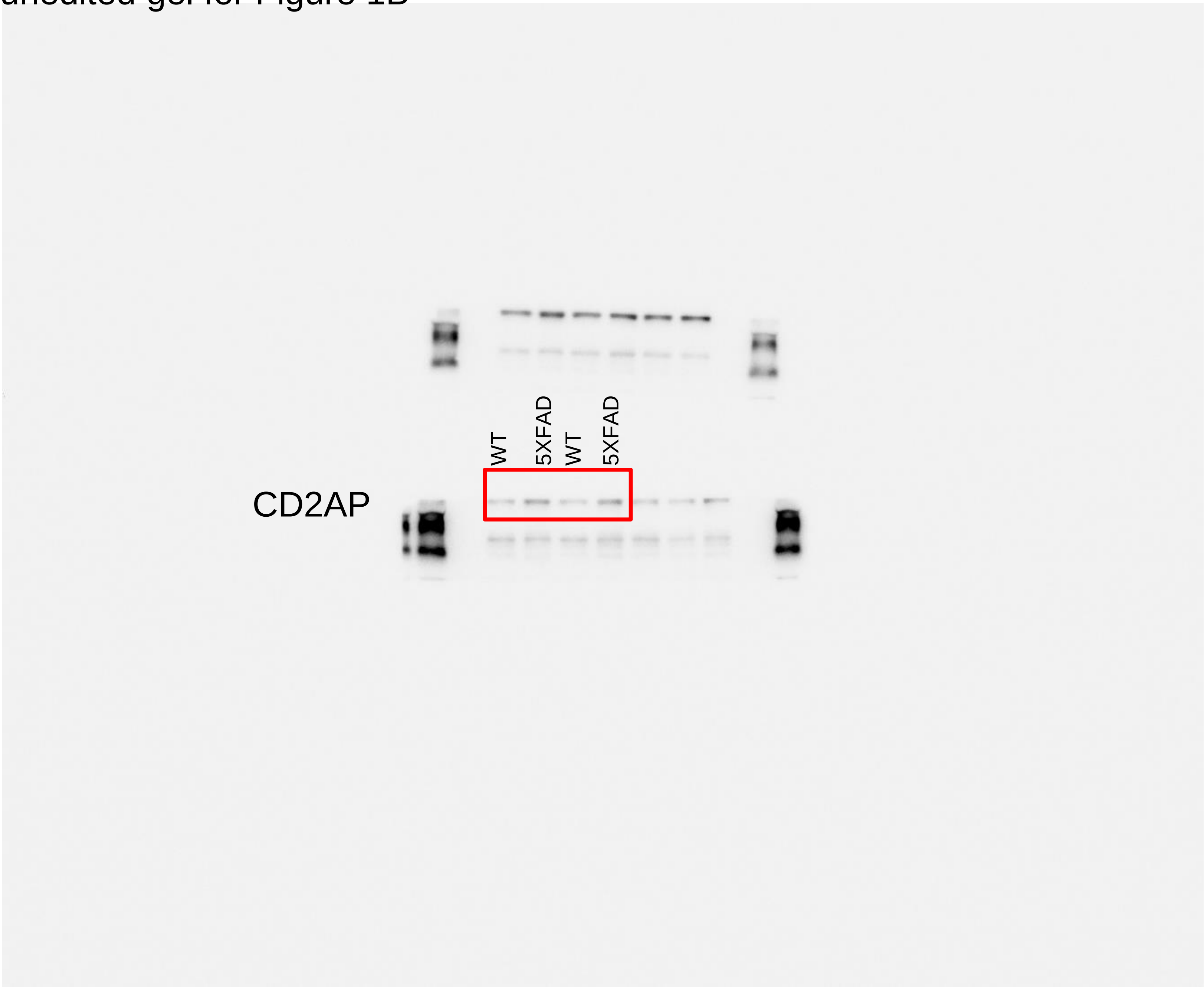

The membrane was imaged with Azure Biosystems 300

Full unedited gel for Figure 1B

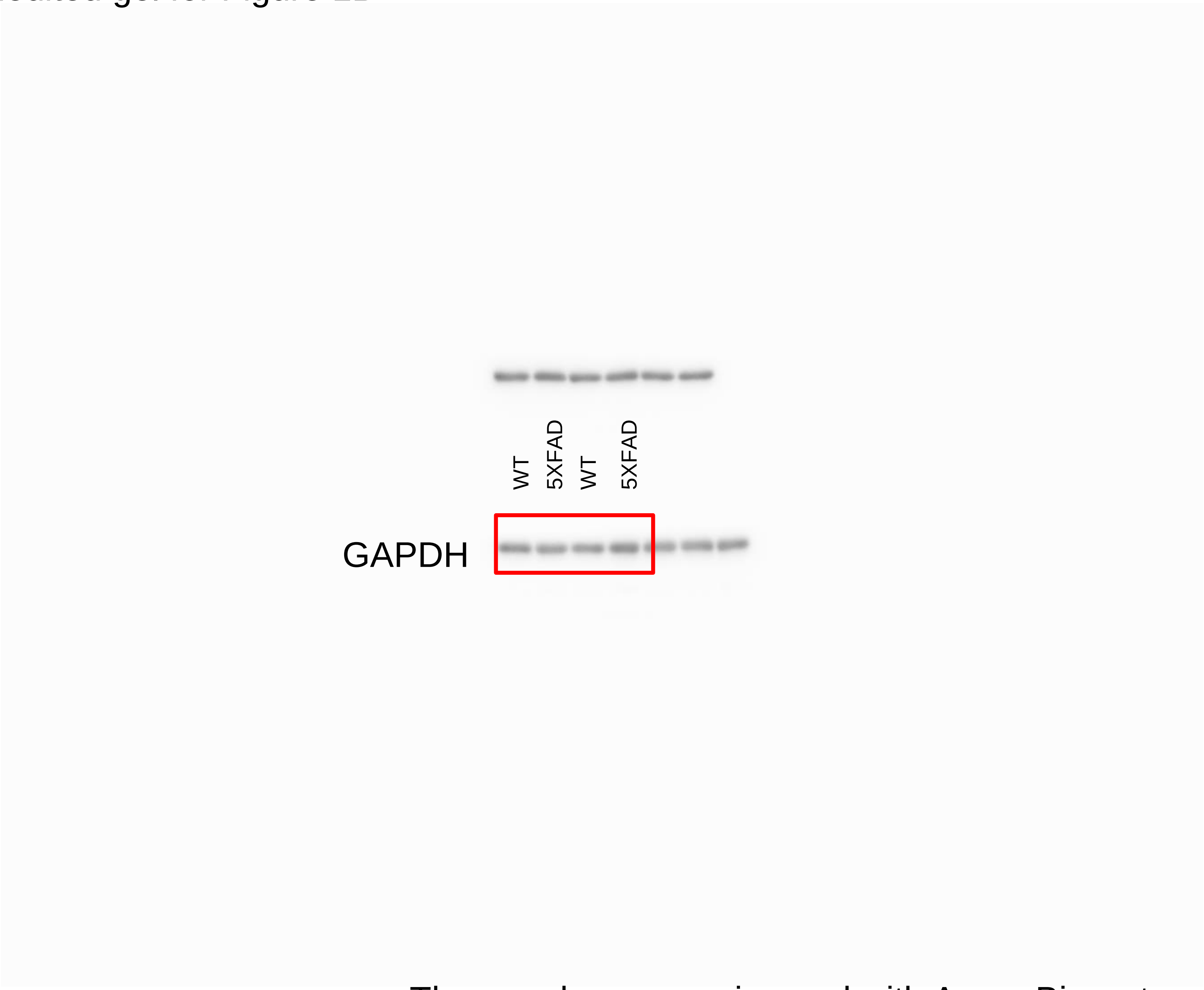

The membrane was imaged with Azure Biosystems 300

Full unedited gel for Figure 1C

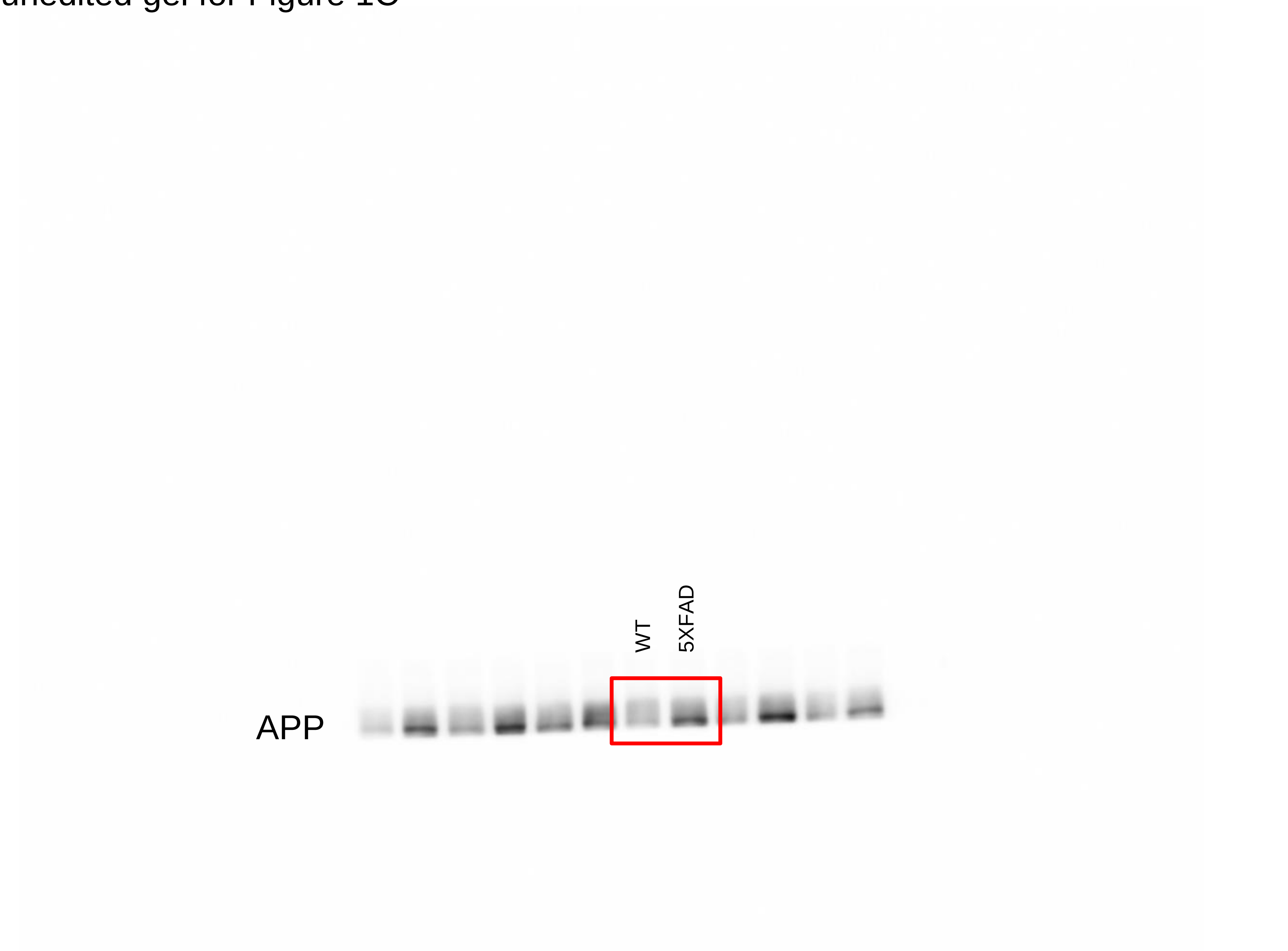

The membrane was imaged with Azure Biosystems 300

Full unedited gel for Figure 1C

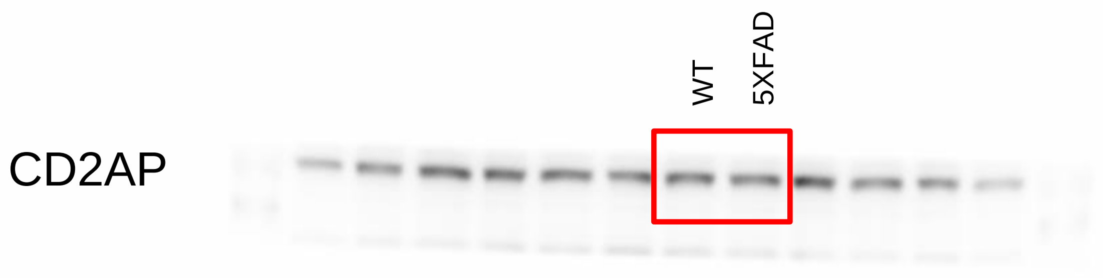

The membrane was imaged with Azure Biosystems 300

Full unedited gel for Figure 1C

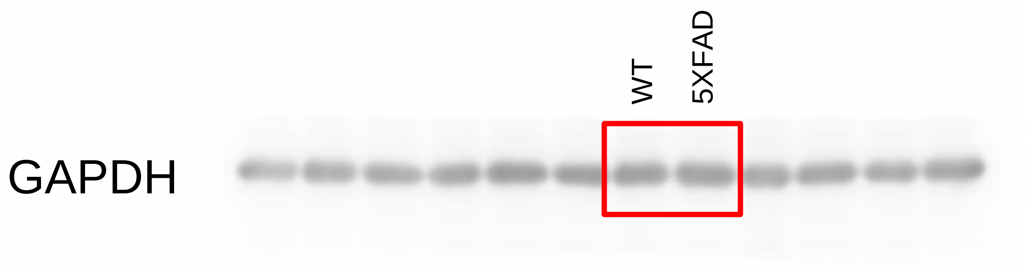

The membrane was imaged with Azure Biosystems 300

Full unedited gel for Figure 1D

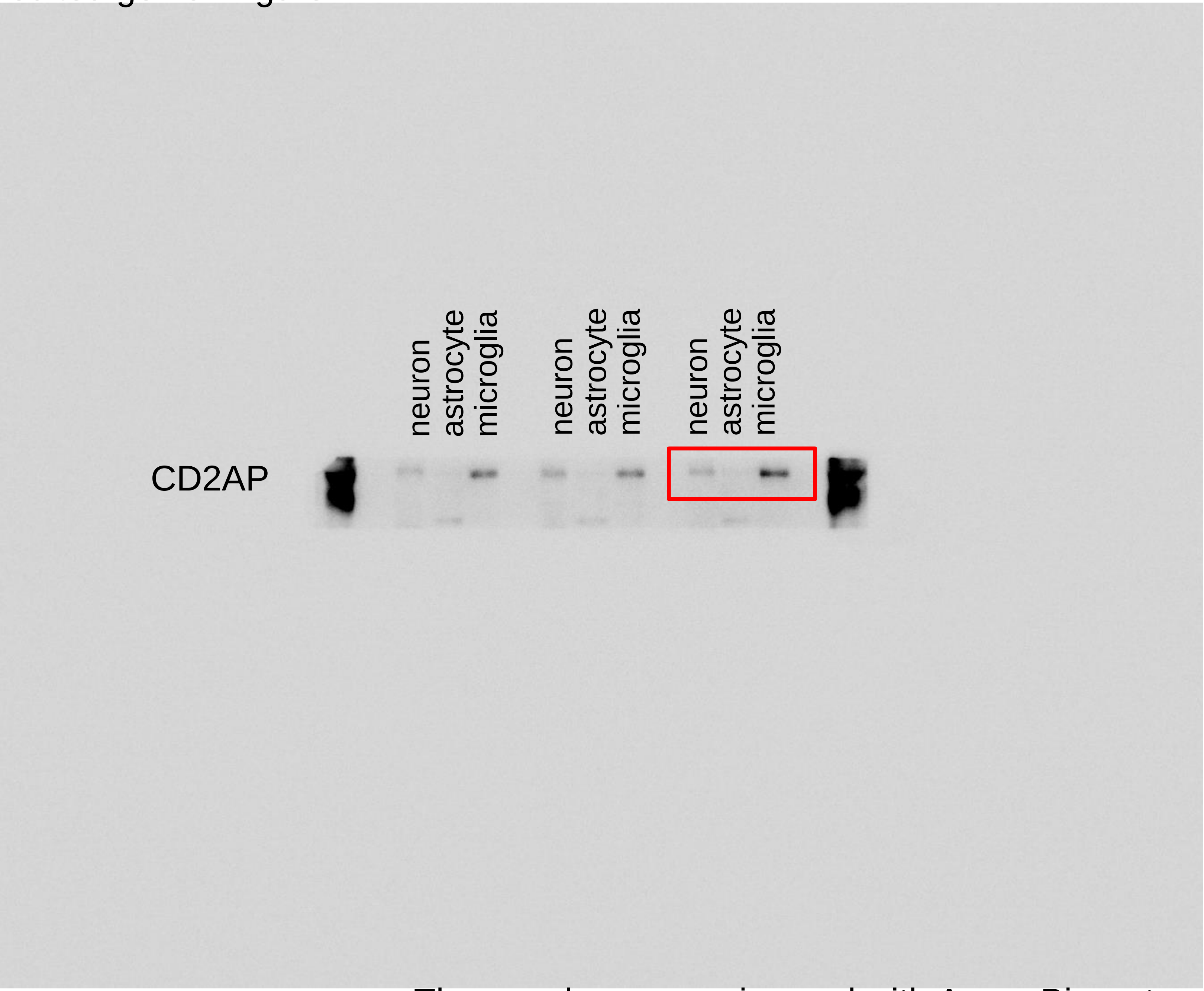

The membrane was imaged with Azure Biosystems 300

Full unedited gel for Figure 1D

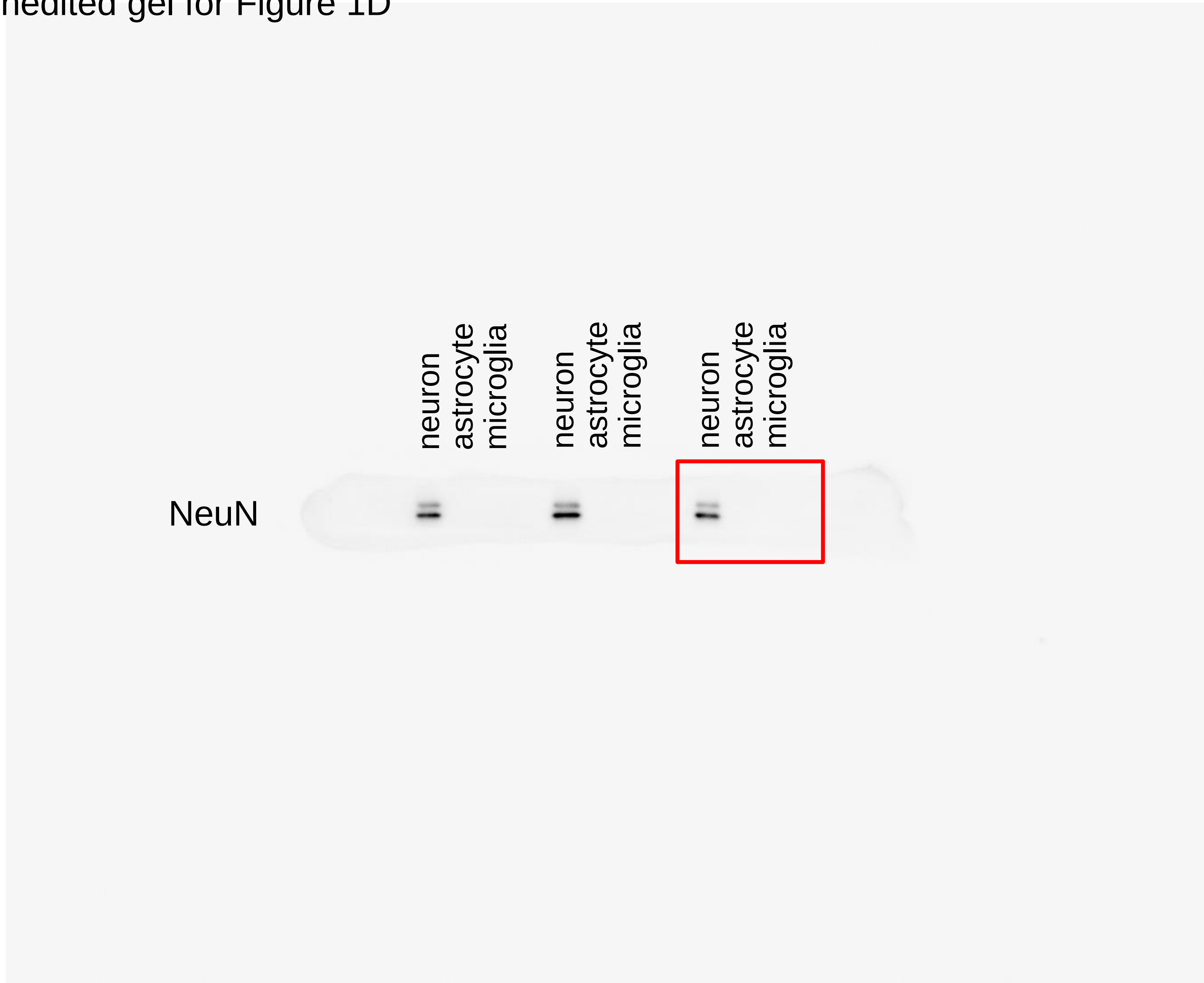

The membrane was imaged with Azure Biosystems 300

Full unedited gel for Figure 1D

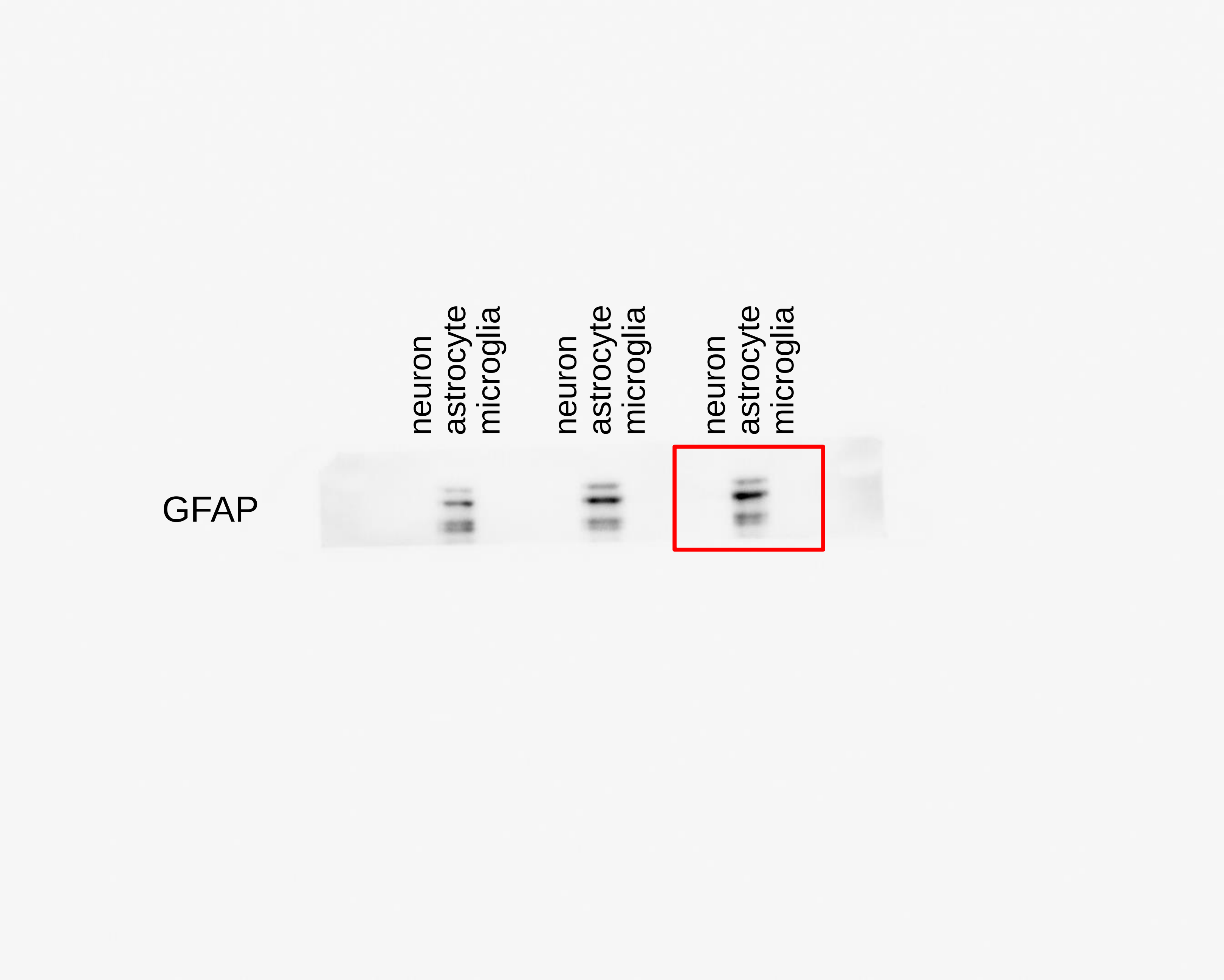

The membrane was imaged with Azure Biosystems 300

Full unedited gel for Figure 1D

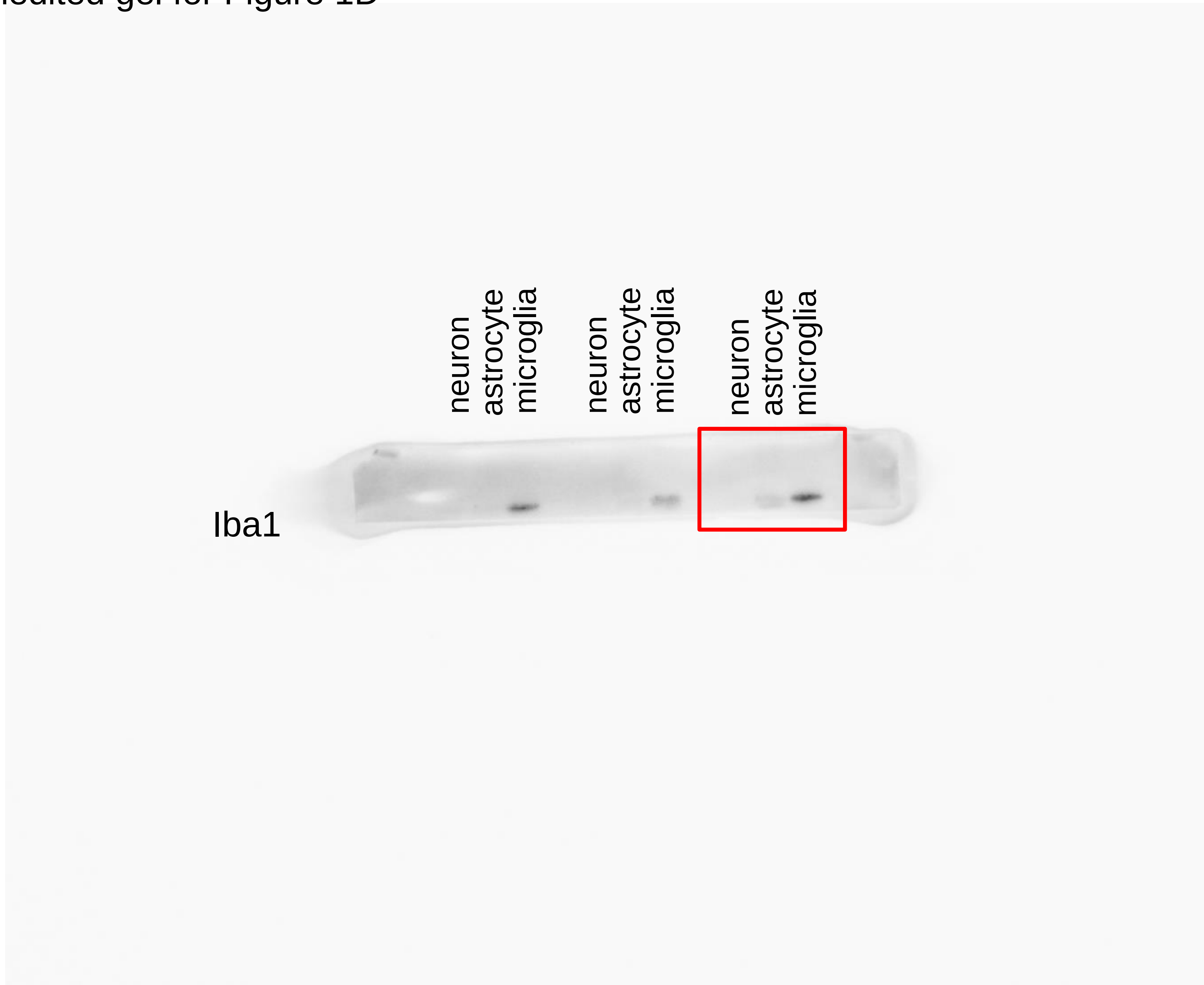

The membrane was imaged with Azure Biosystems 300

Full unedited gel for Figure 1D

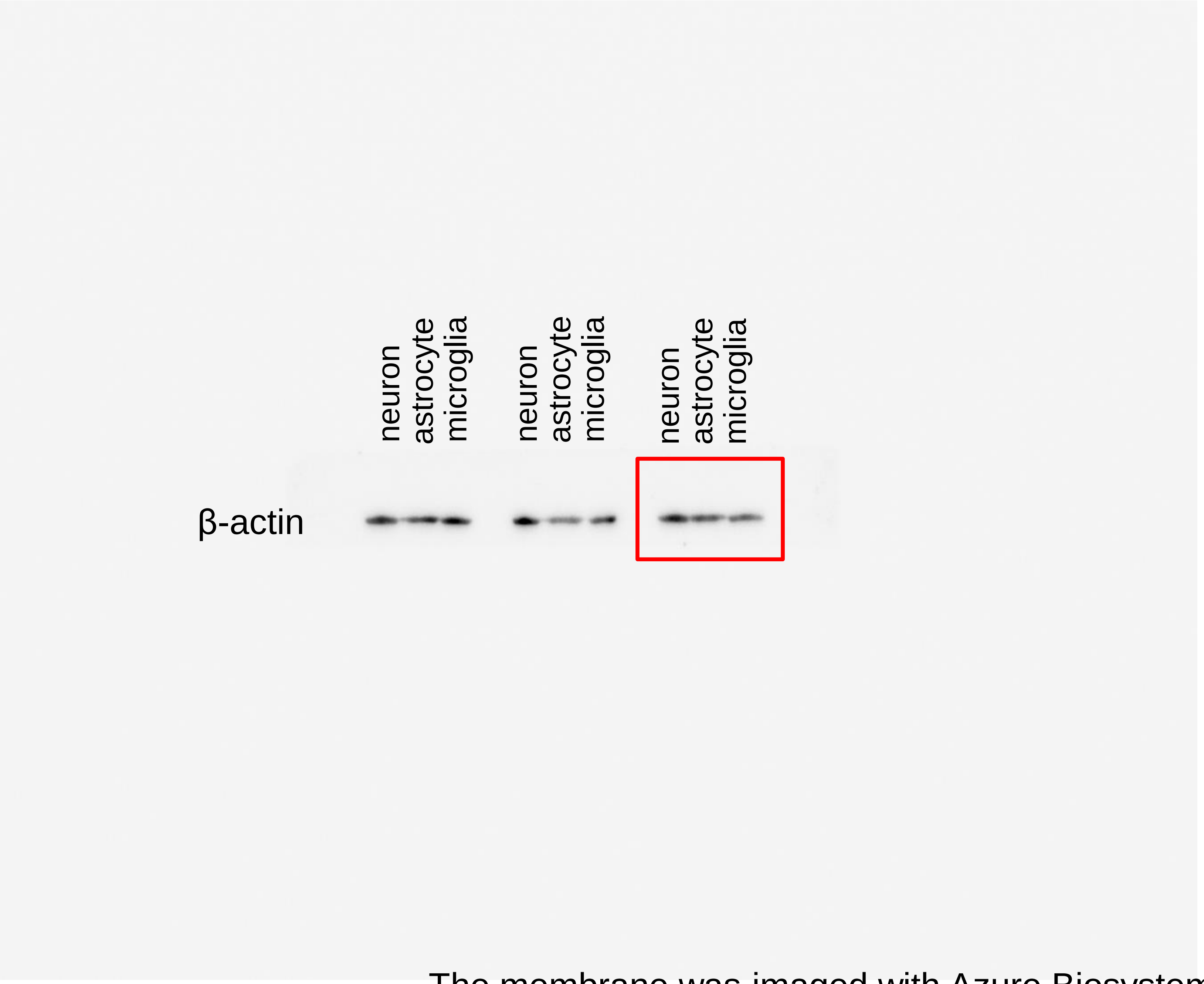

The membrane was imaged with Azure Biosystems 300

Full unedited gel for Figure 1E

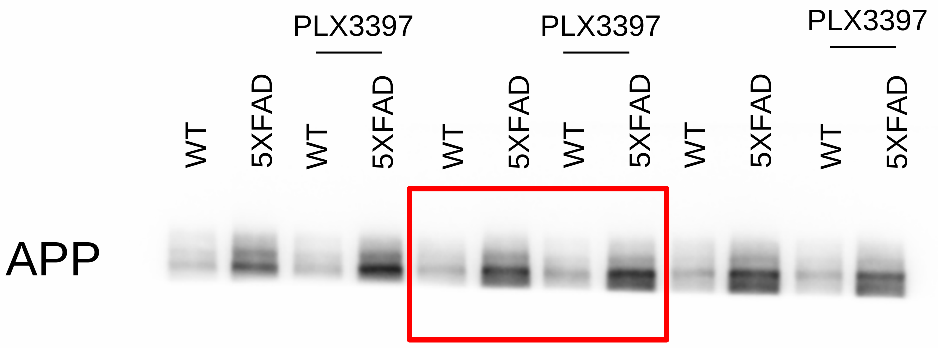

The membrane was imaged with Azure Biosystems 300

Full unedited gel for Figure 1E

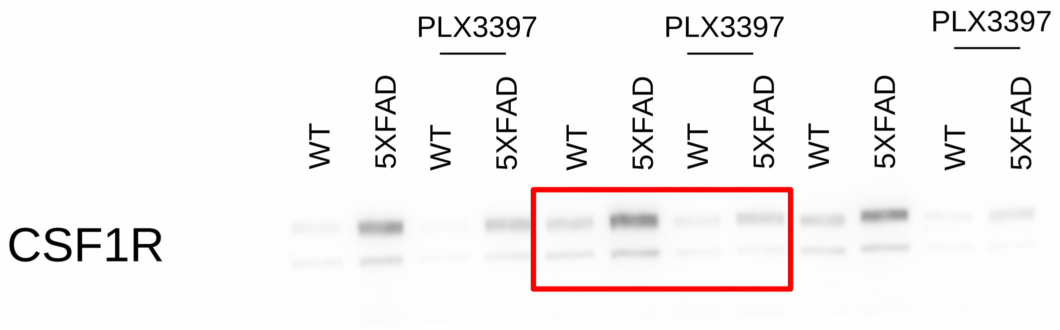

The membrane was imaged with Azure Biosystems 300

Full unedited gel for Figure 1E

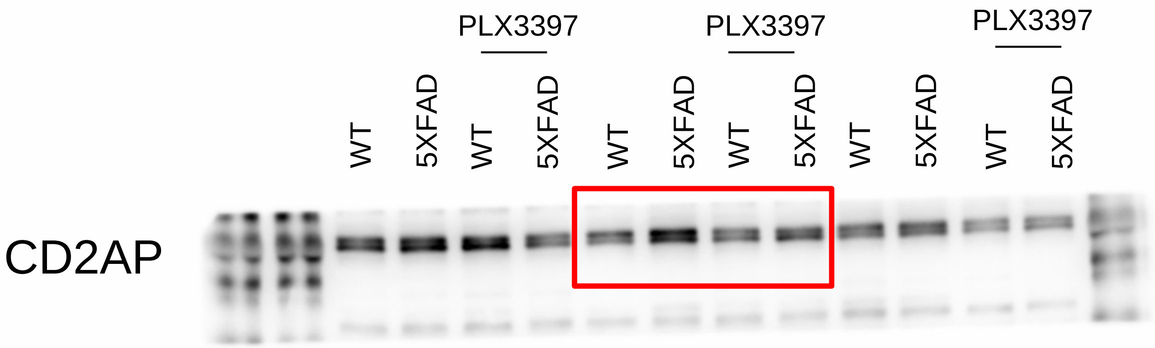

The membrane was imaged with Azure Biosystems 300

Full unedited gel for Figure 1E

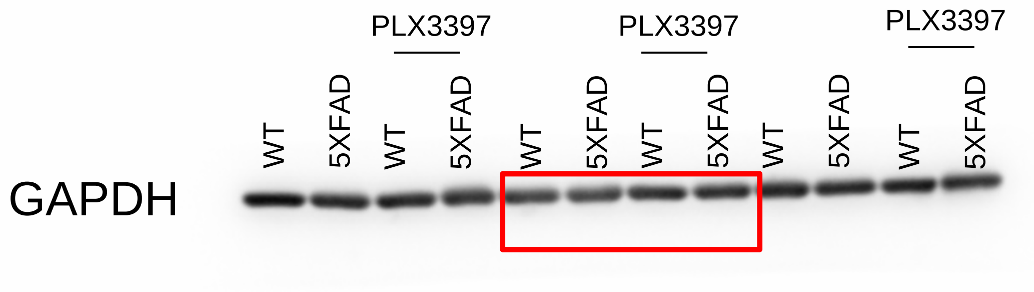

The membrane was imaged with Azure Biosystems 300

Full unedited gel for Figure 7B

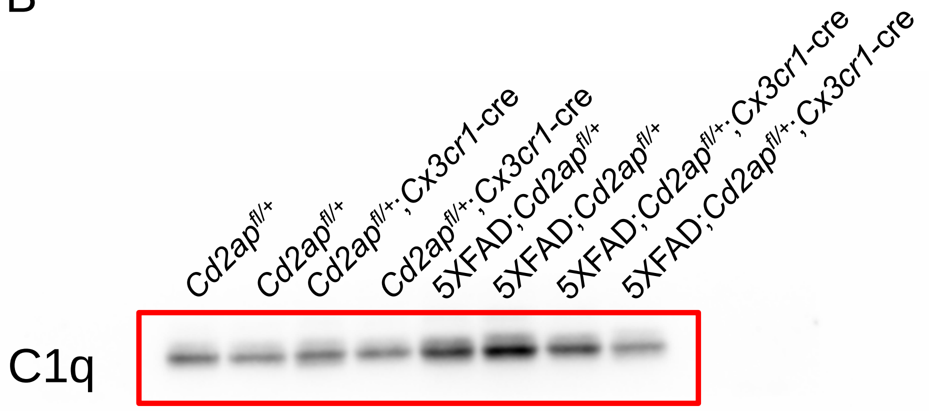

The membrane was imaged with Azure Biosystems 300

Full unedited gel for Figure 7B

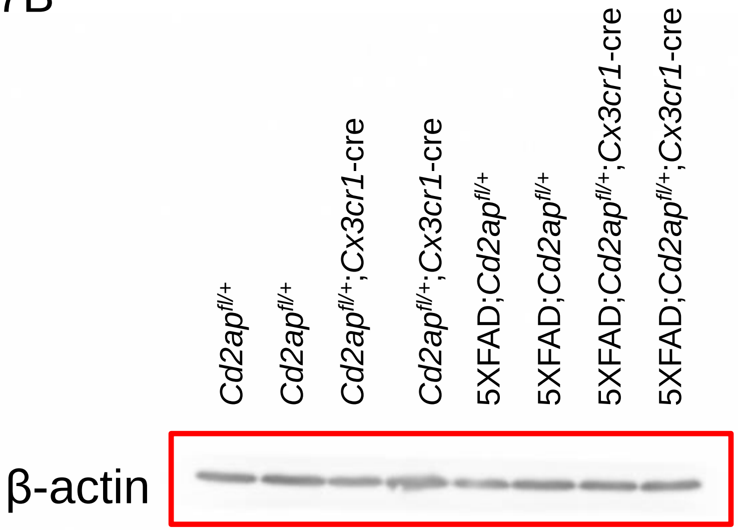

The membrane was imaged with Azure Biosystems 300

Full unedited gel for Figure 8A

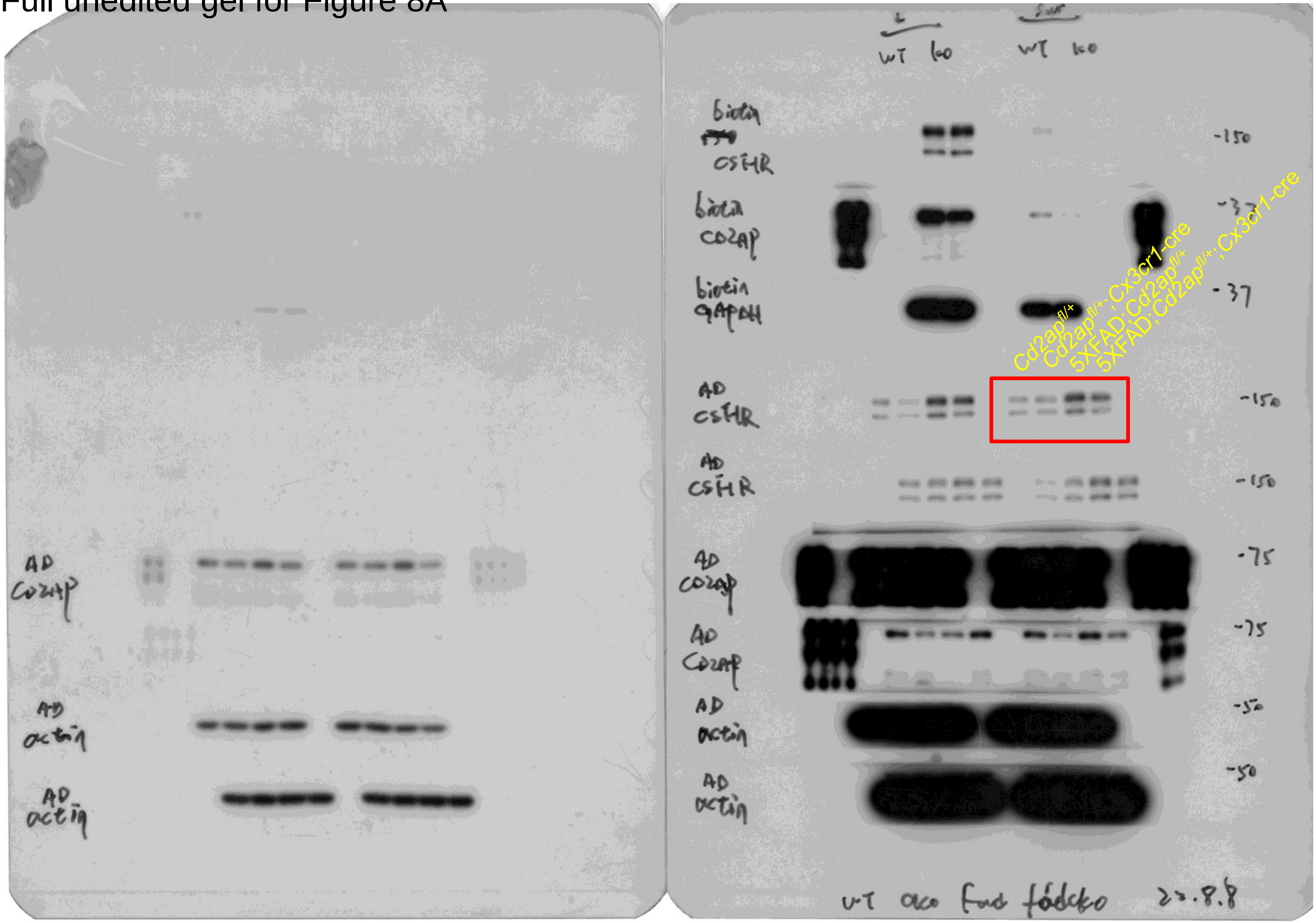

Full unedited gel for Figure 8A

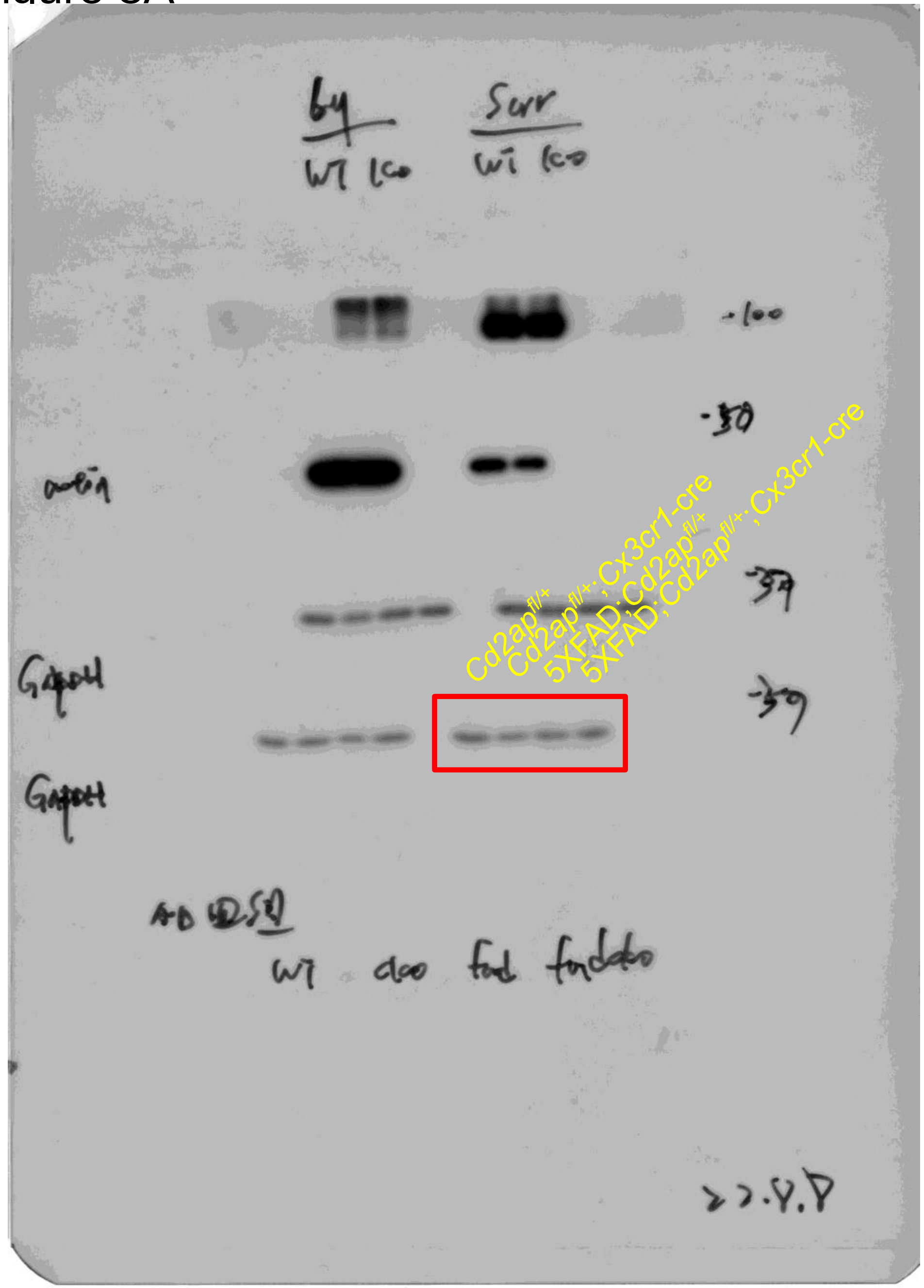

Full unedited gel for Figure 8B

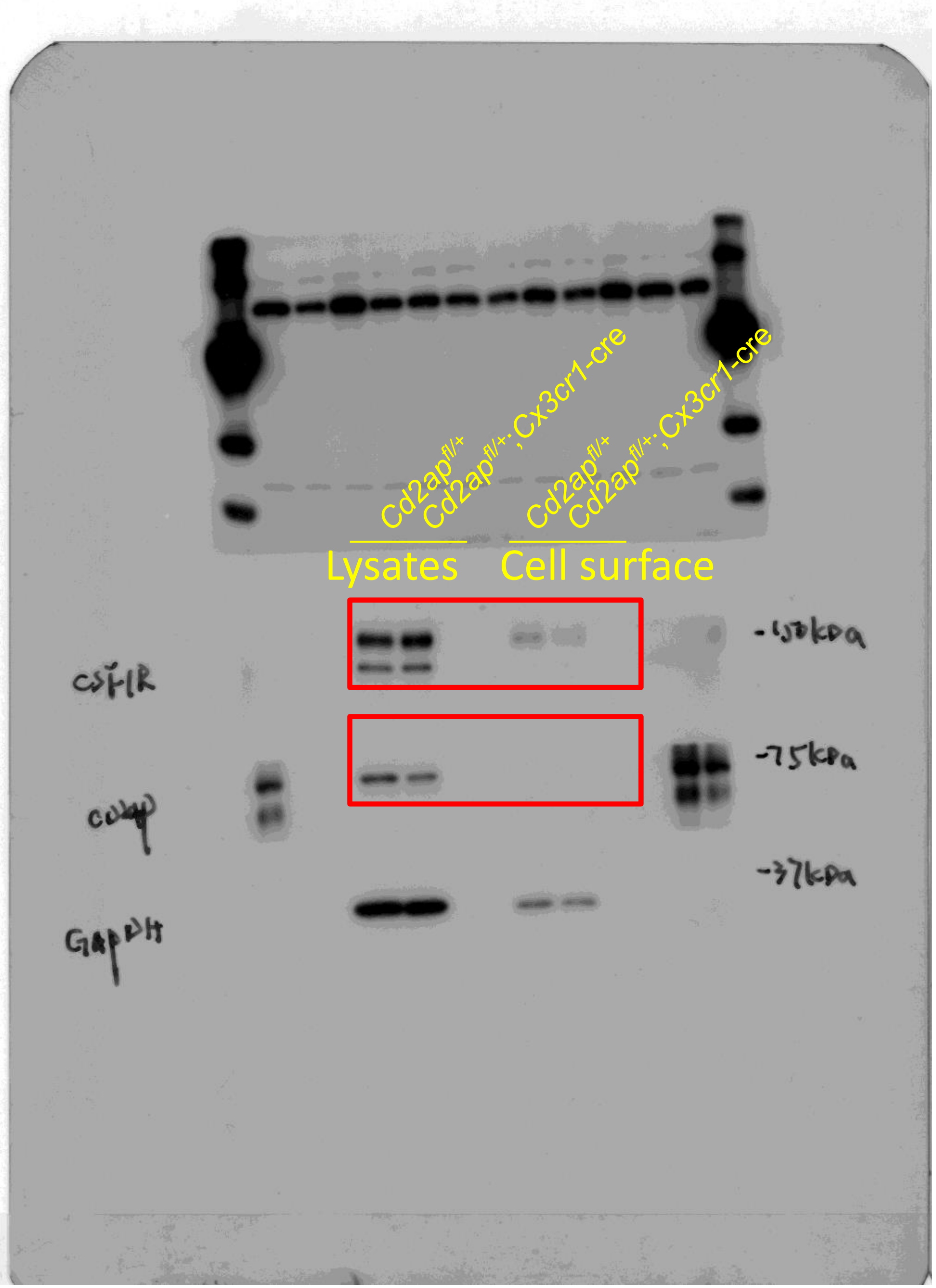

Full unedited gel for Figure 8B

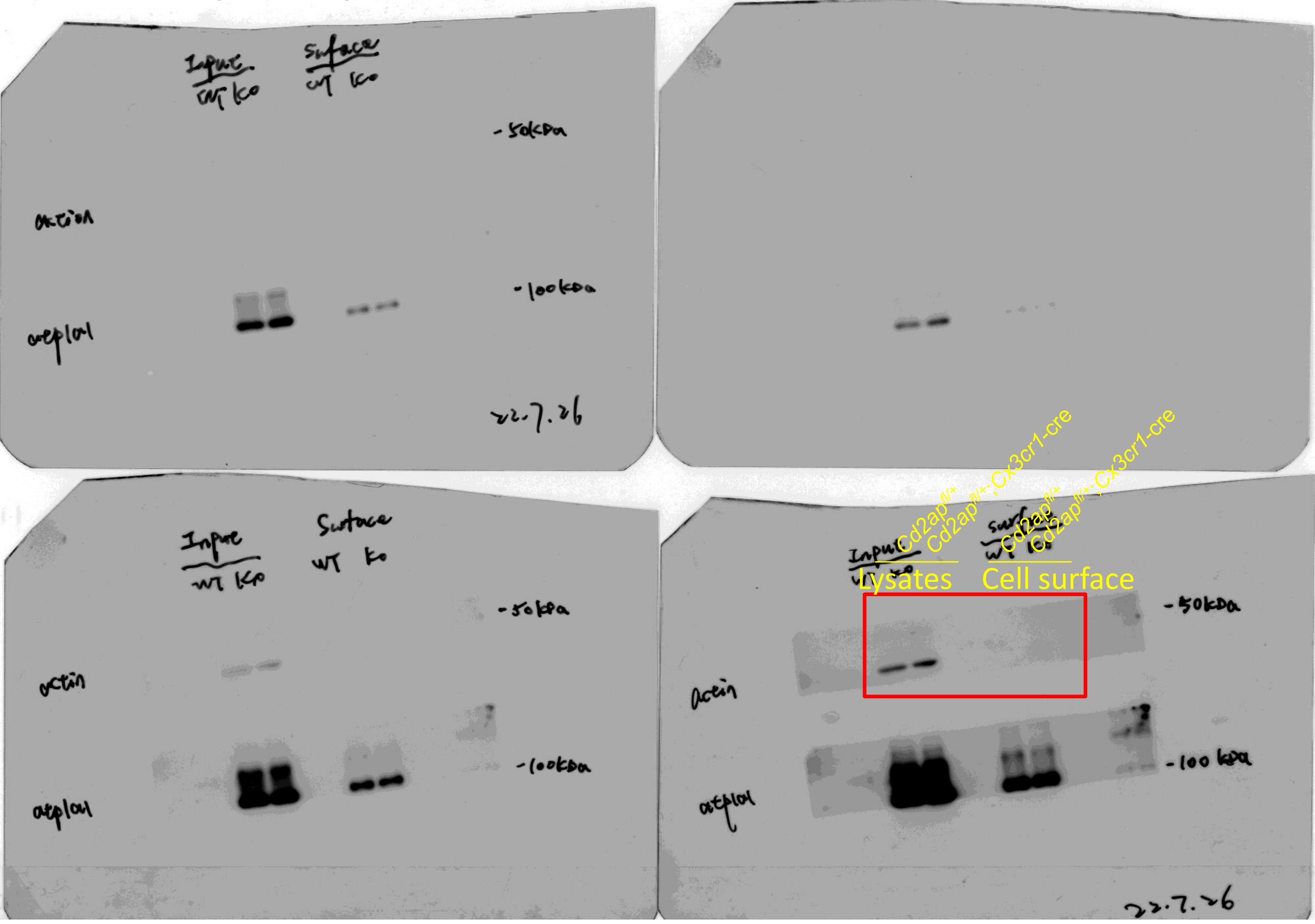

Full unedited gel for Figure 8B

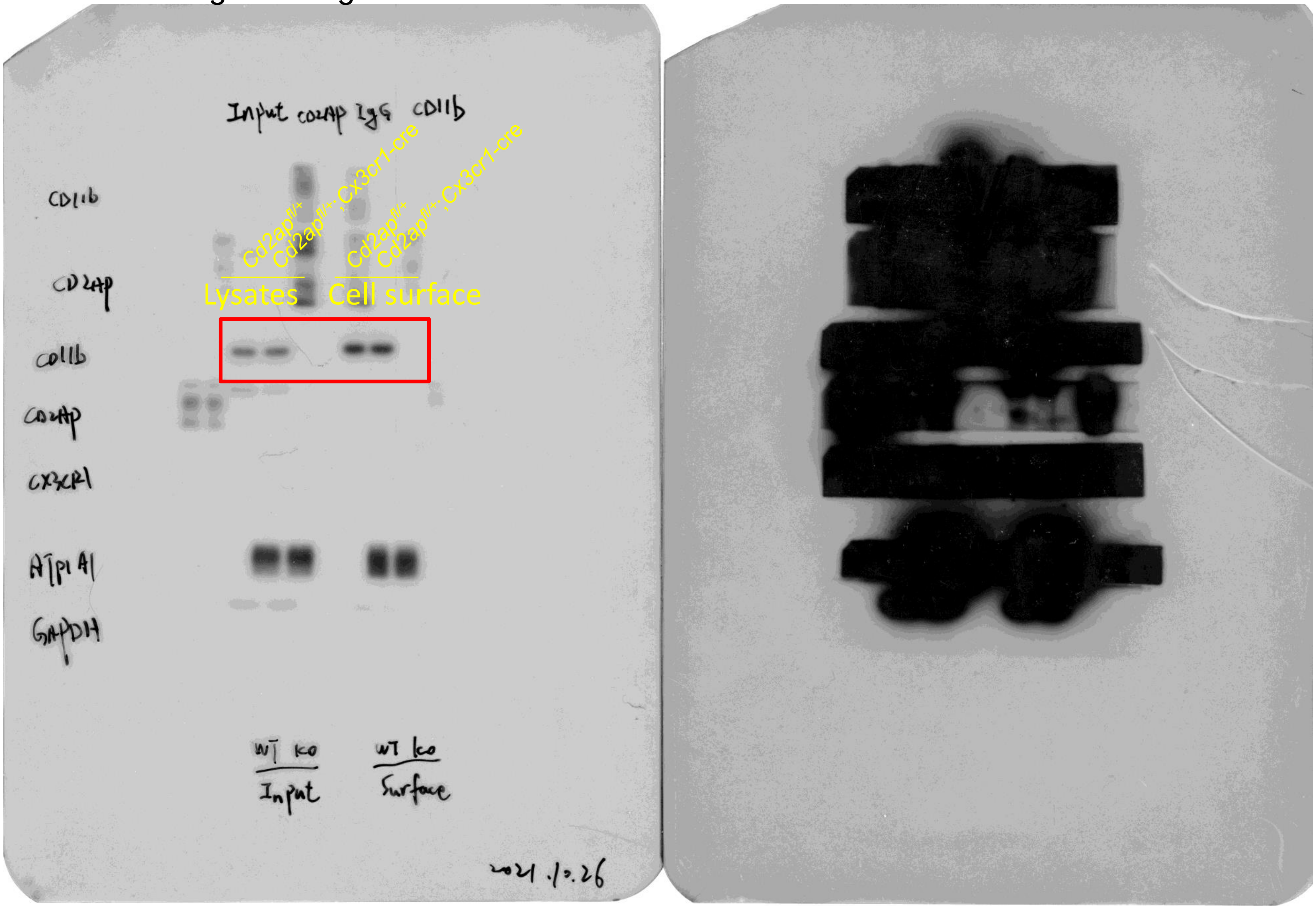

Full unedited gel for Figure 8B

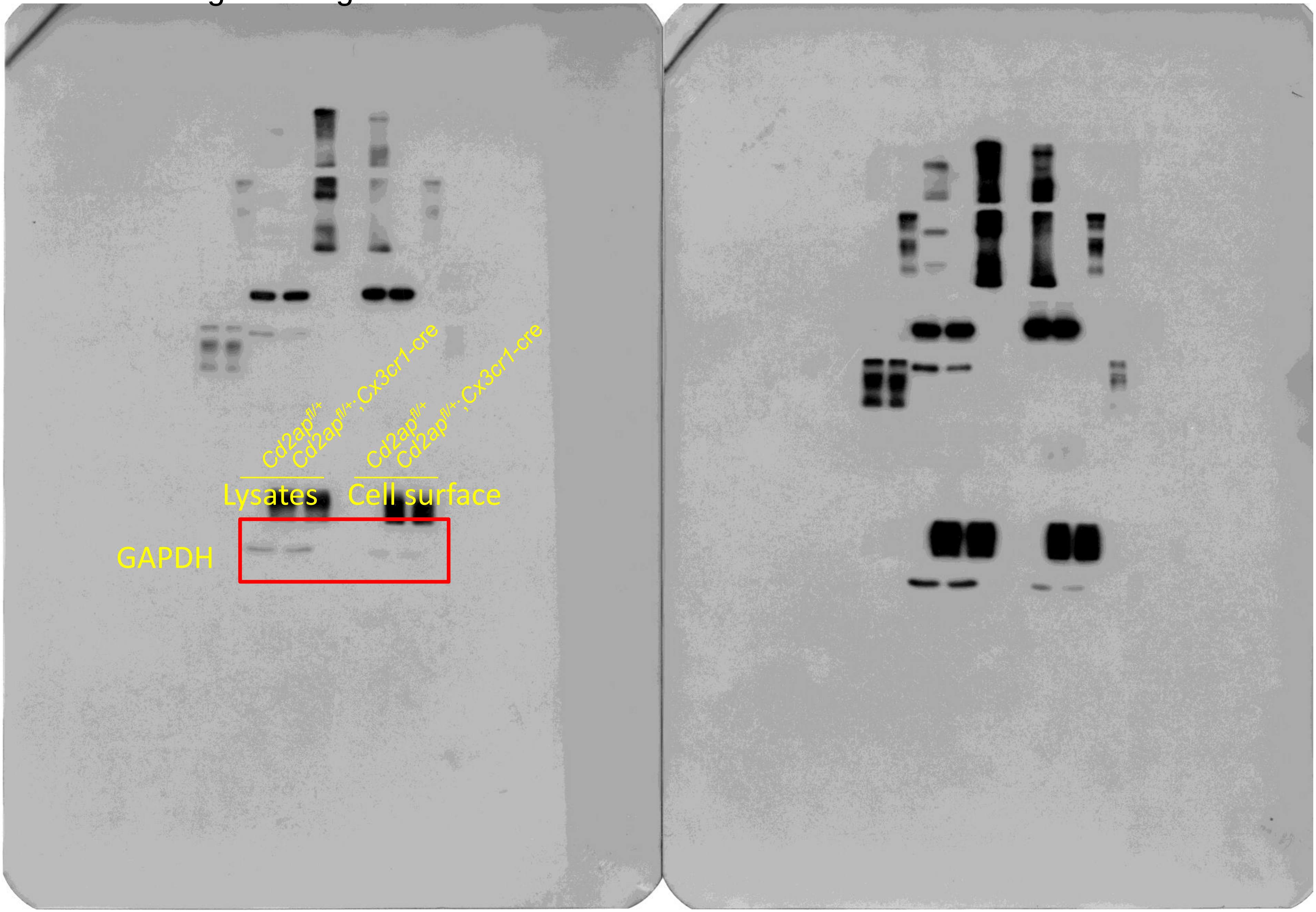

Full unedited gel for Figure 8D

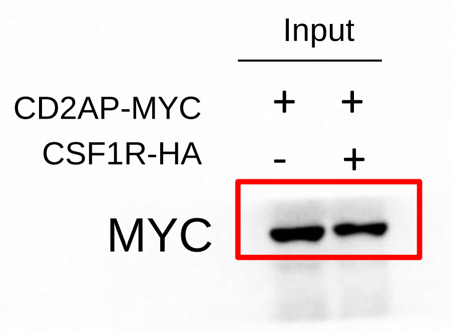

The membrane was imaged with Azure Biosystems 300

Full unedited gel for Figure 8D

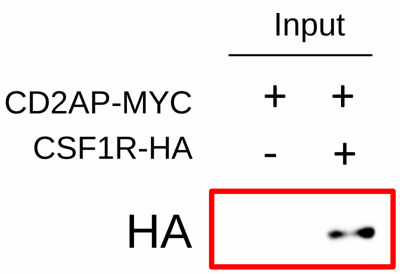

The membrane was imaged with Azure Biosystems 300

Full unedited gel for Figure 8D

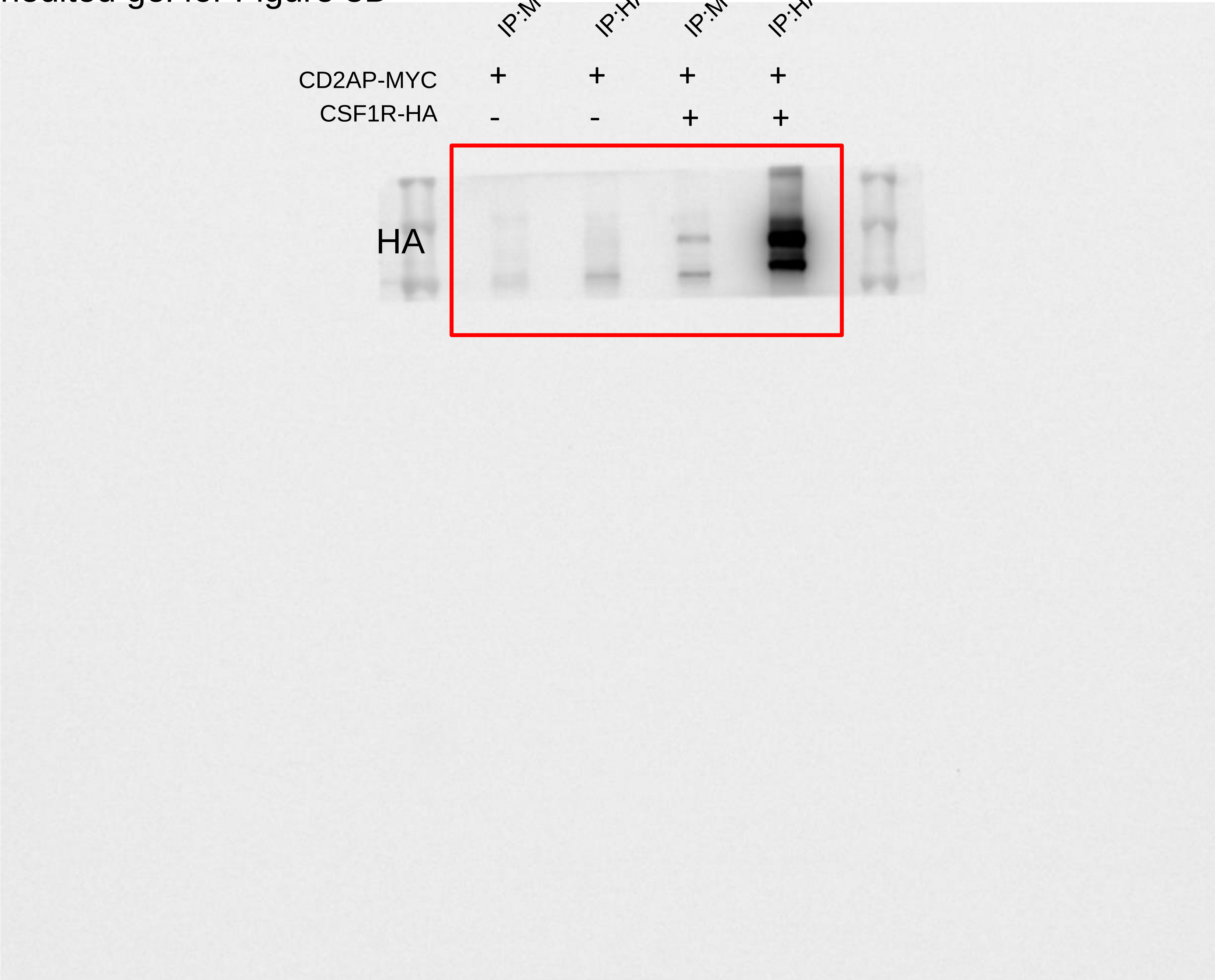

The membrane was imaged with Azure Biosystems 300

Full unedited gel for Supplemental Figure 3D

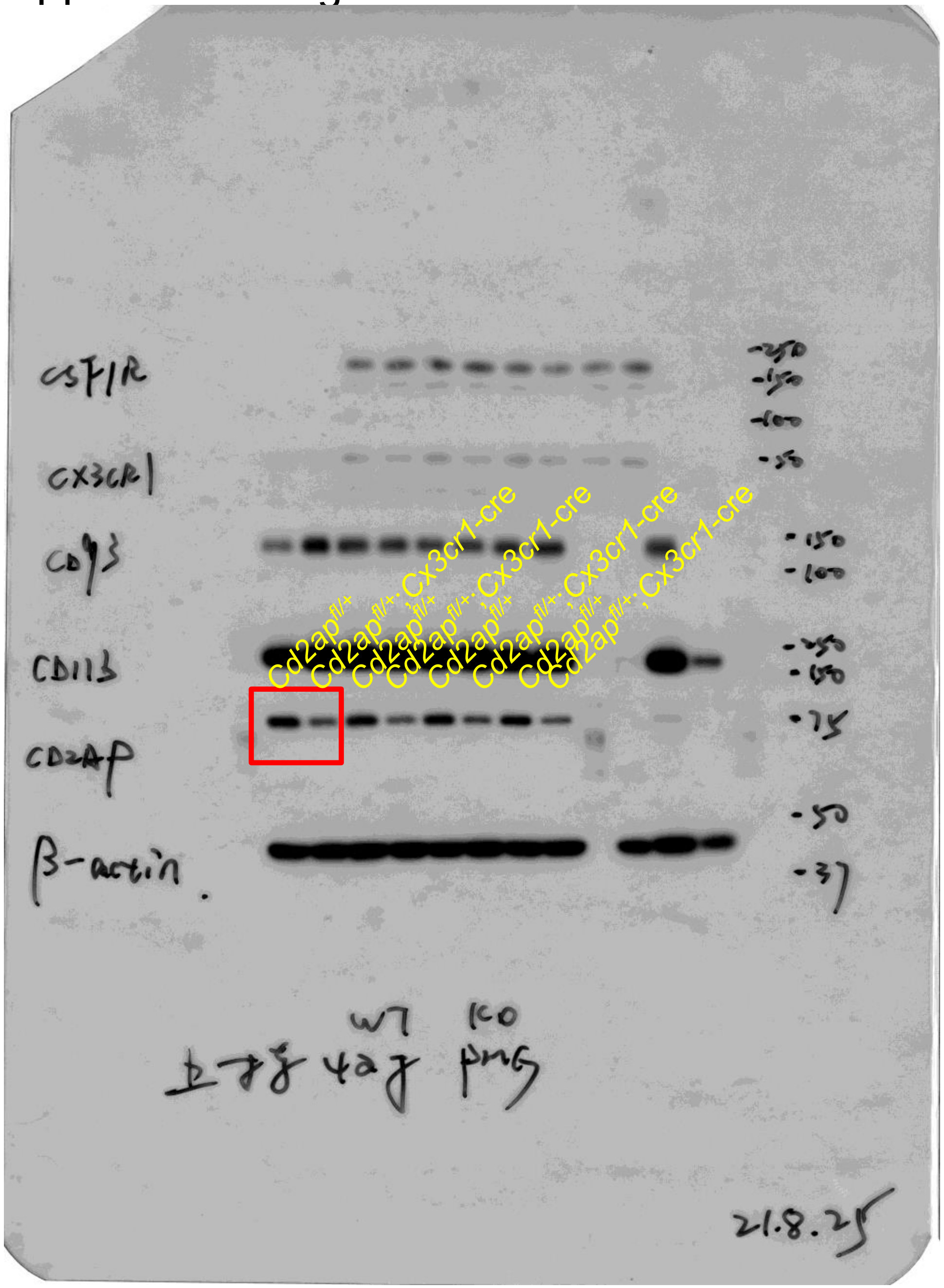

Full unedited gel for Supplemental Figure 3D

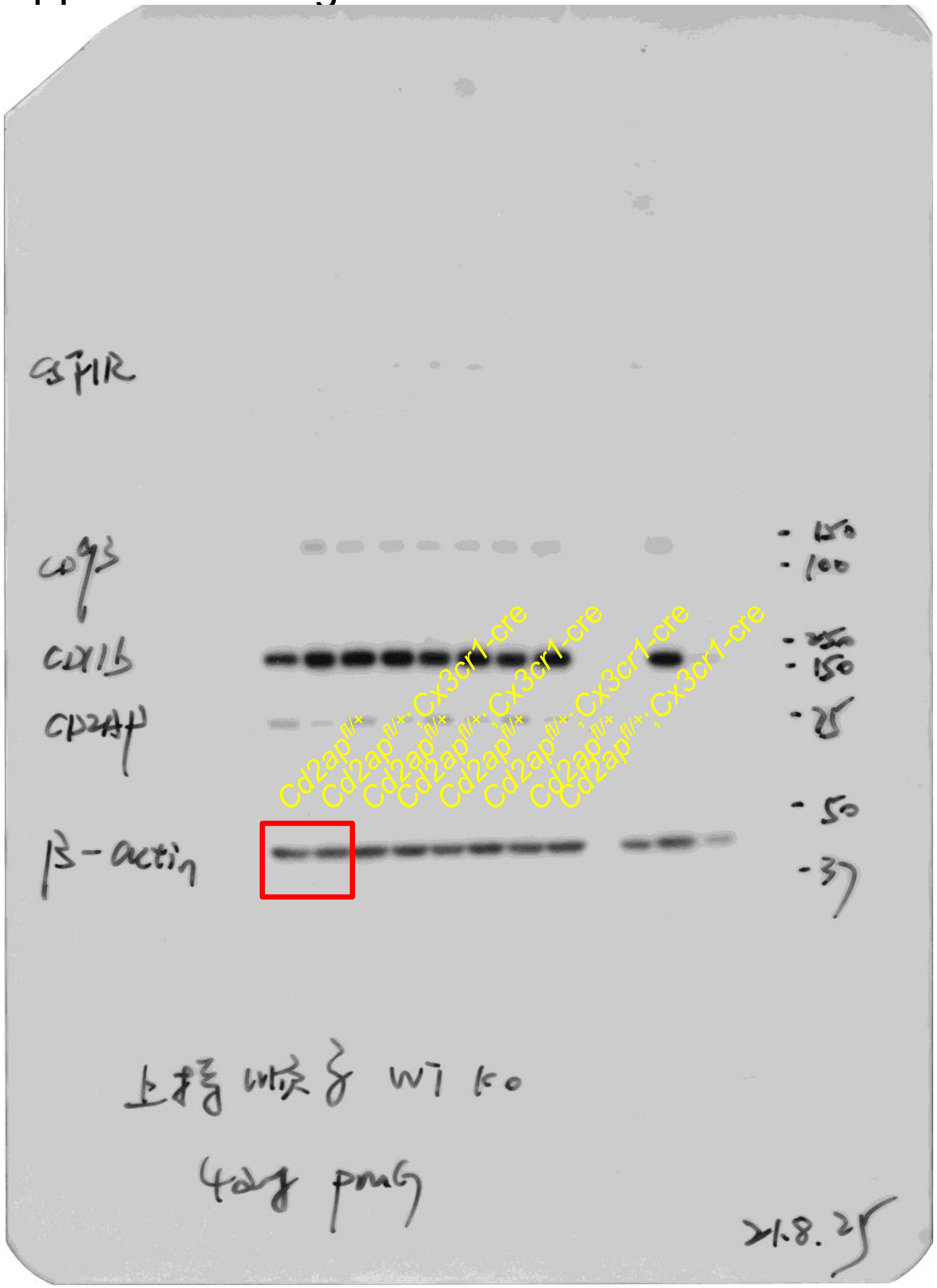

Full unedited gel for Supplemental Figure 3E

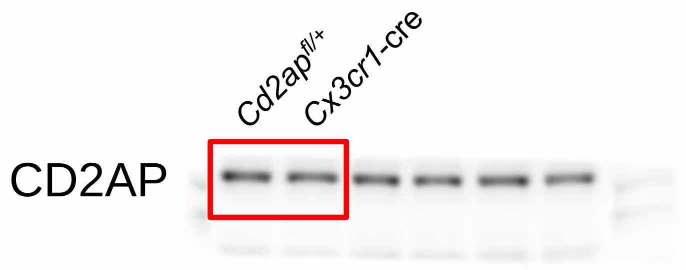

The membrane was imaged with Azure Biosystems 300

Full unedited gel for Supplemental Figure 3E

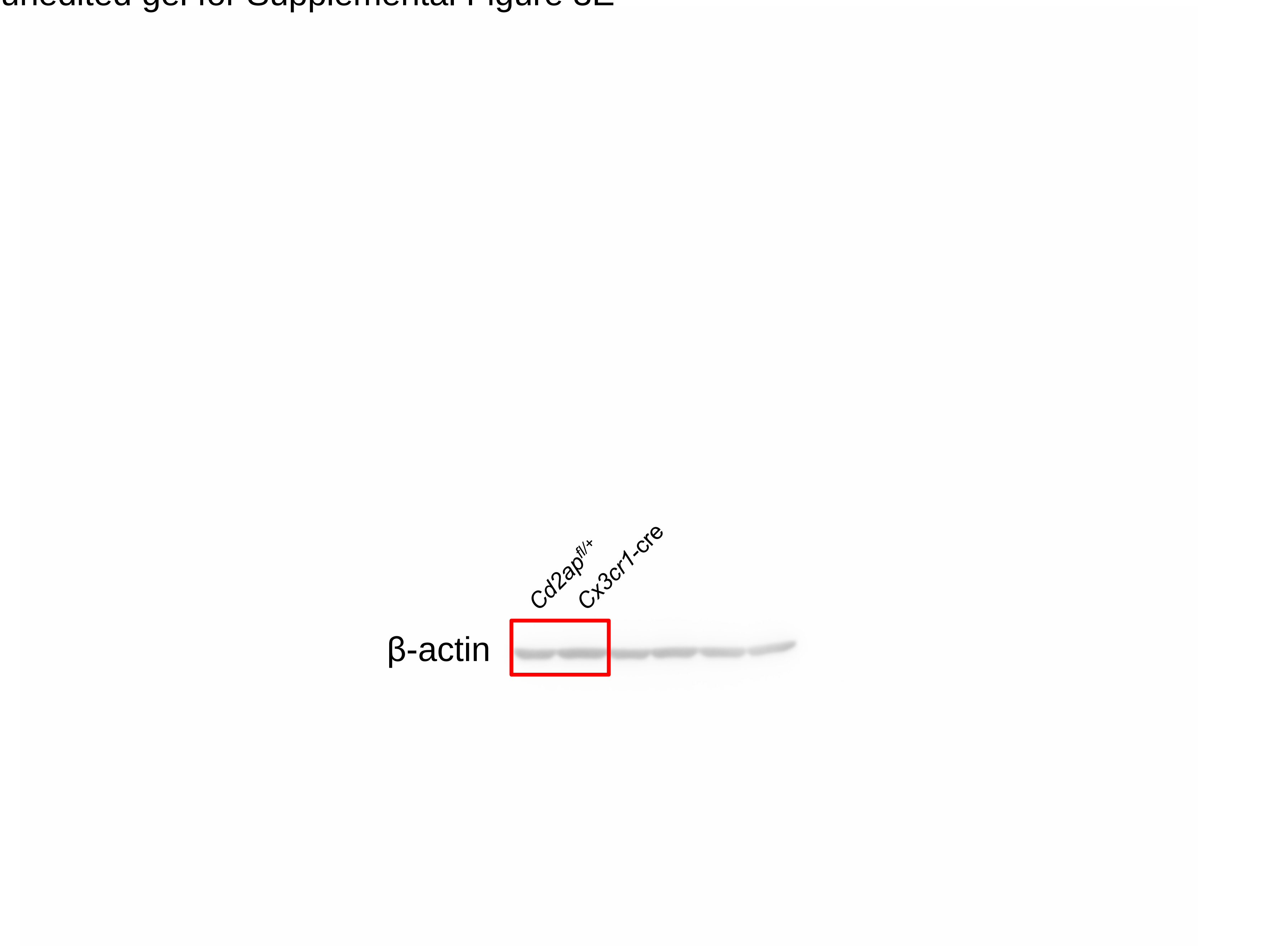

The membrane was imaged with Azure Biosystems 300

Full unedited gel for Supplemental Figure 3F

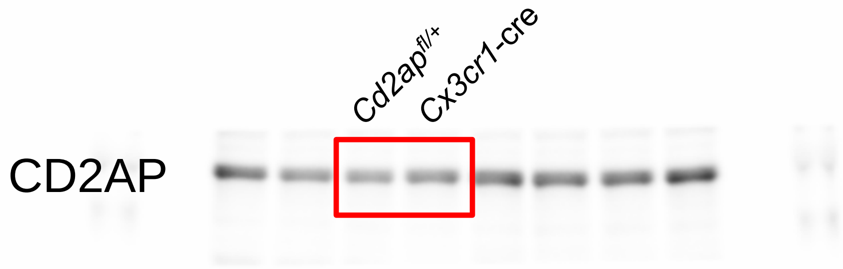

The membrane was imaged with Azure Biosystems 300

Full unedited gel for Supplemental Figure 3F

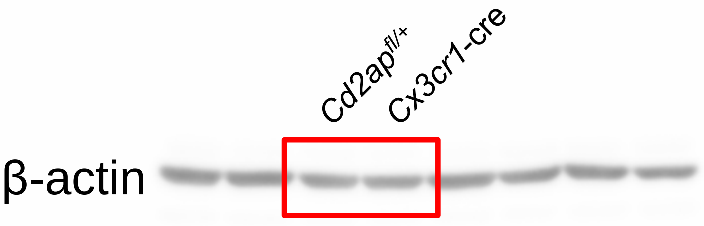

The membrane was imaged with Azure Biosystems 300

Full unedited gel for Supplemental Figure 5B

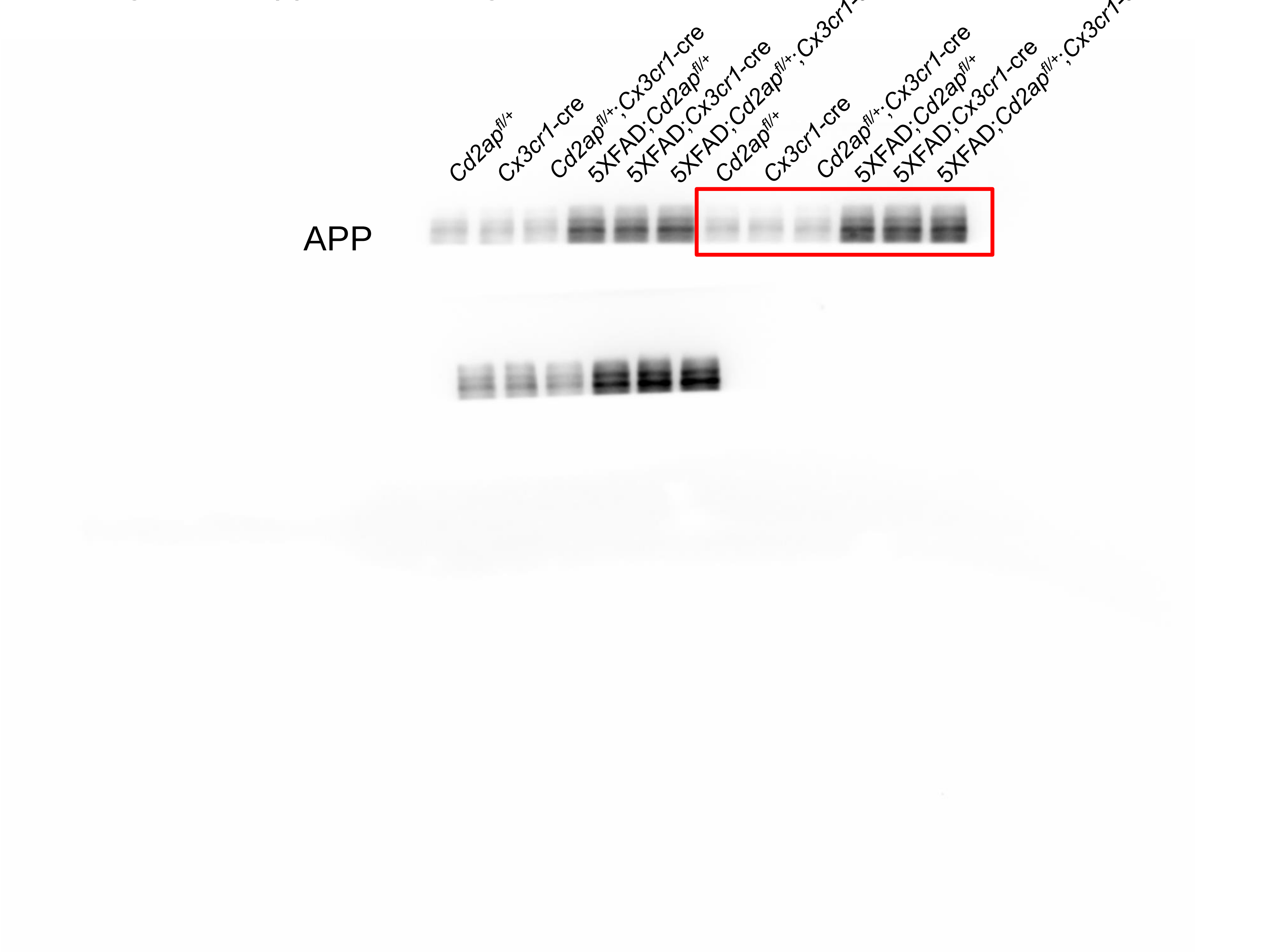

The membrane was imaged with Azure Biosystems 300

Full unedited gel for Supplemental Figure 5B

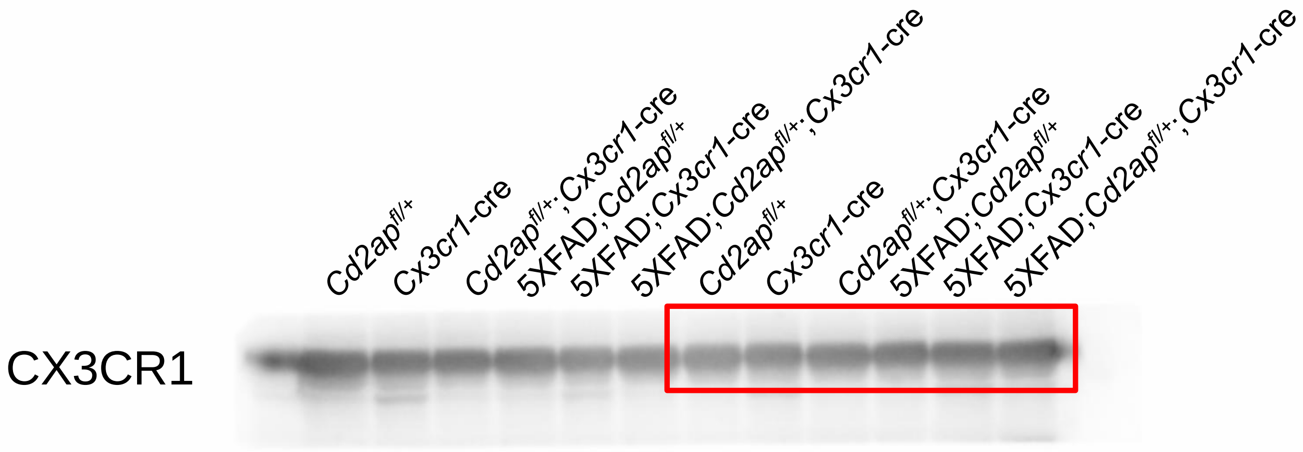

The membrane was imaged with Azure Biosystems 300

Full unedited gel for Supplemental Figure 5B

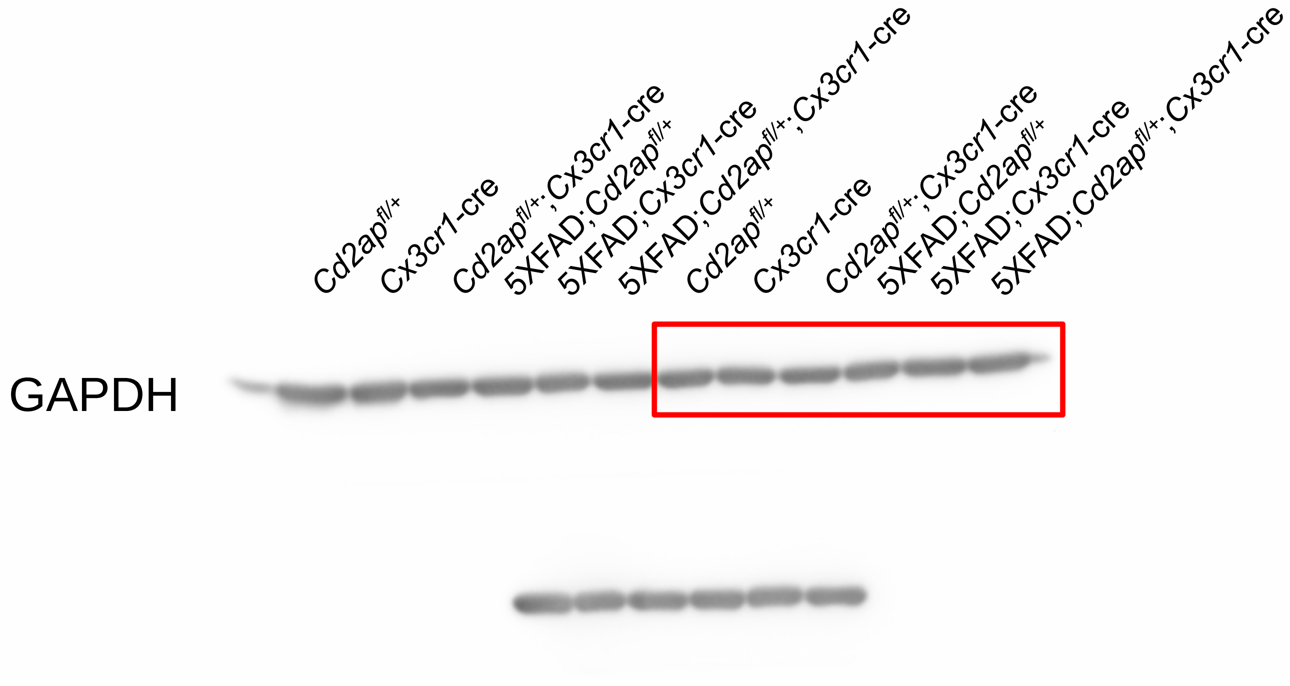

The membrane was imaged with Azure Biosystems 300

Full unedited gel for Supplemental Figure 7A

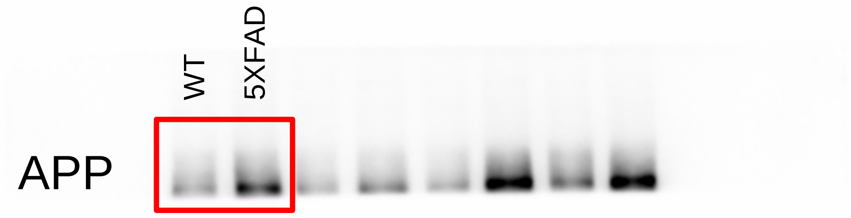

The membrane was imaged with Azure Biosystems 300

Full unedited gel for Supplemental Figure 7A

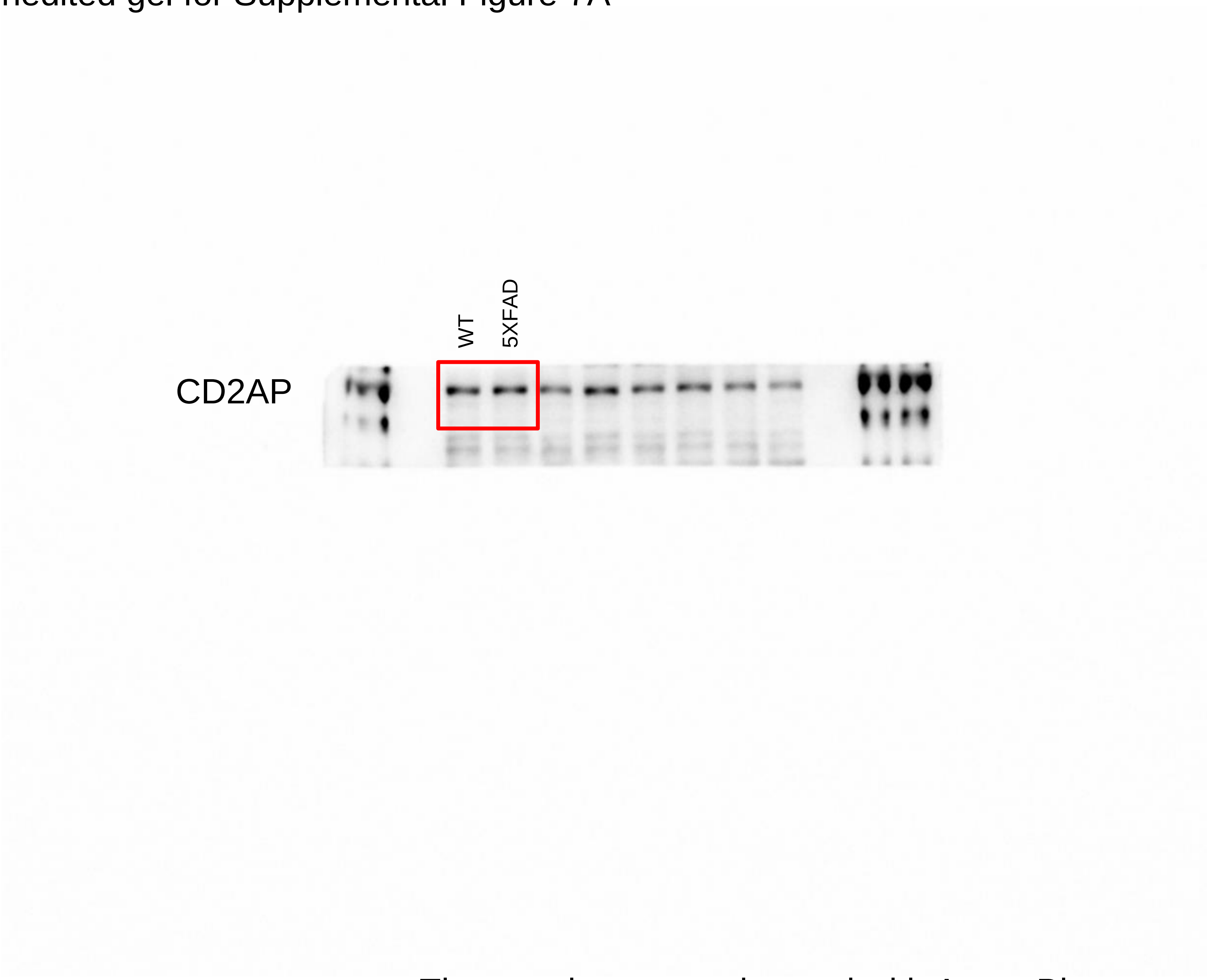

The membrane was imaged with Azure Biosystems 300

Full unedited gel for Supplemental Figure 7A

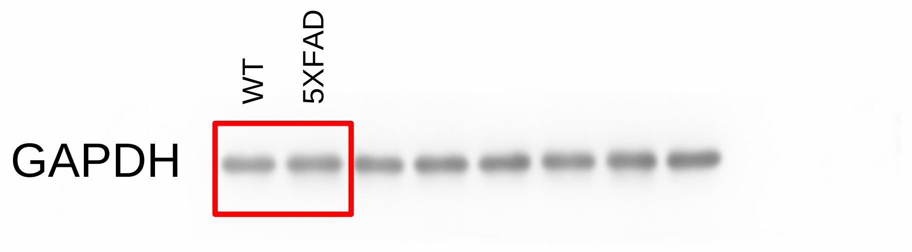

The membrane was imaged with Azure Biosystems 300

Full unedited gel for Supplemental Figure 7F

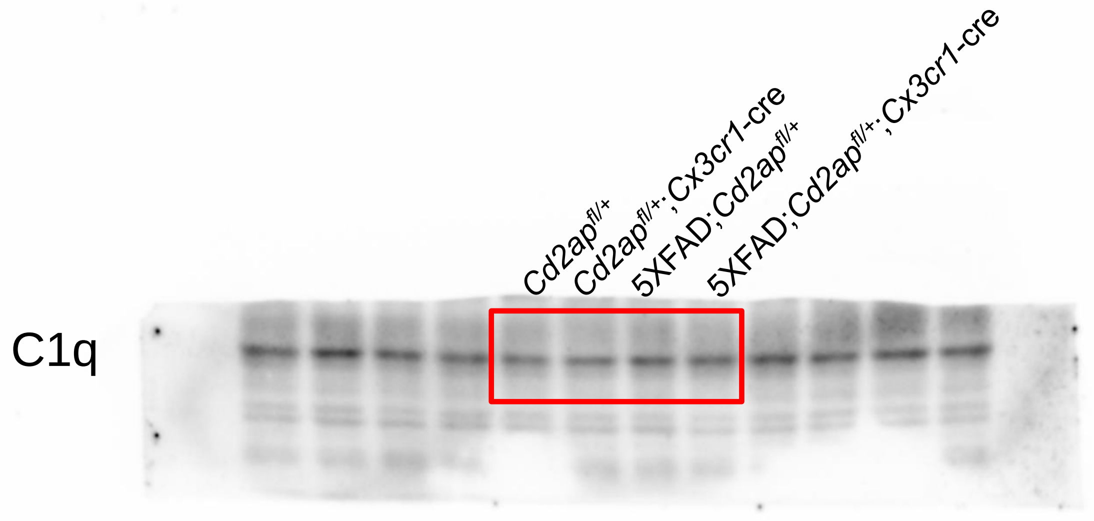

The membrane was imaged with Azure Biosystems 300

Full unedited gel for Supplemental Figure 7F

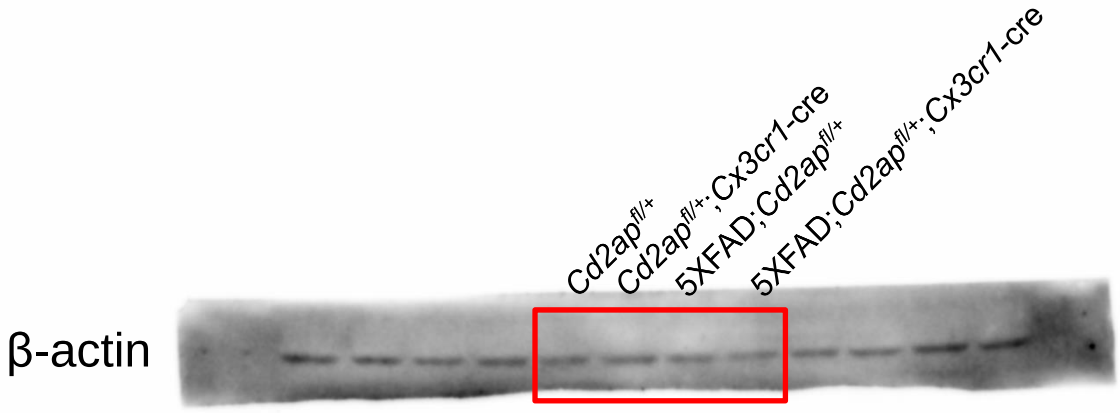

The membrane was imaged with Azure Biosystems 300

Full unedited gel for Supplemental Figure 8G

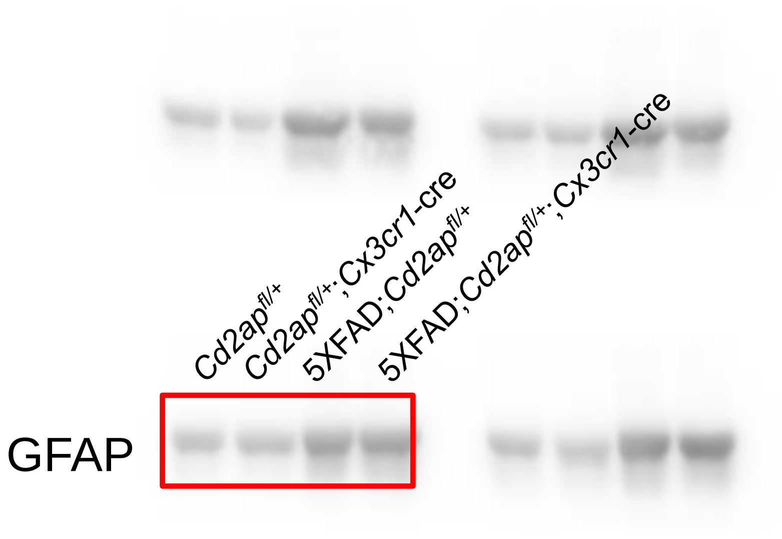

The membrane was imaged with Azure Biosystems 300

Full unedited gel for Supplemental Figure 8G

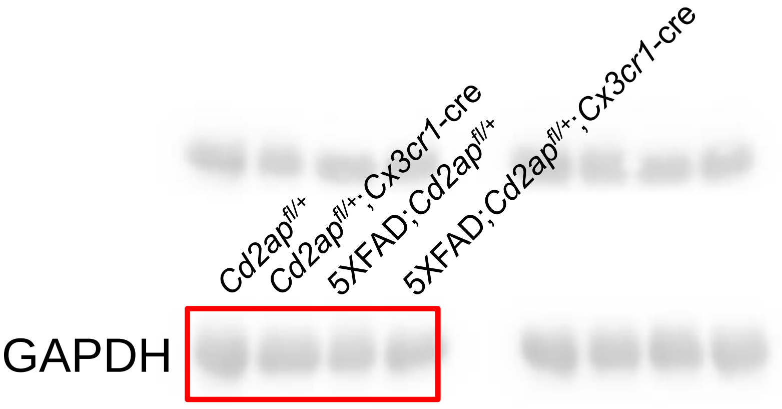

The membrane was imaged with Azure Biosystems 300

Full unedited gel for Supplemental Figure 9A

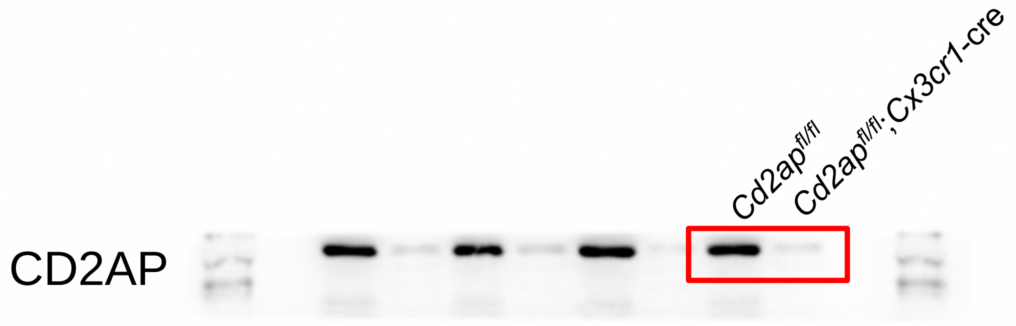

The membrane was imaged with Azure Biosystems 300

Full unedited gel for Supplemental Figure 9A

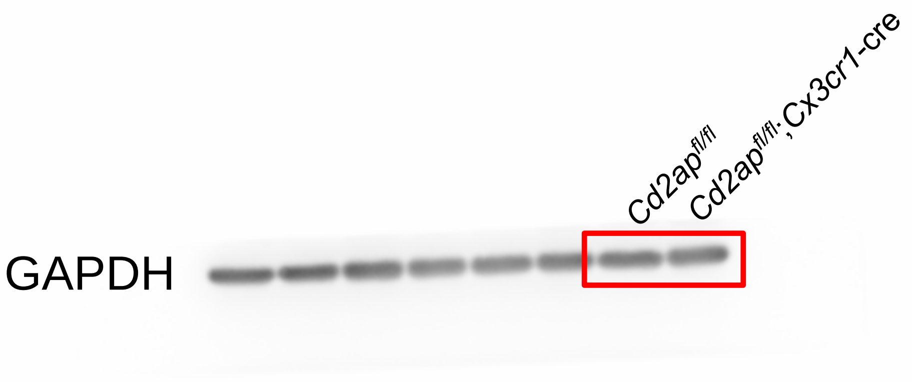

The membrane was imaged with Azure Biosystems 300

Full unedited gel for Supplemental Figure 9F

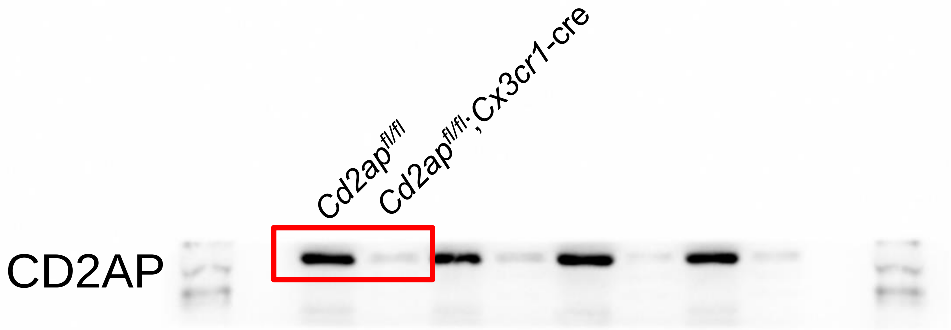

The membrane was imaged with Azure Biosystems 300

Full unedited gel for Supplemental Figure 9F

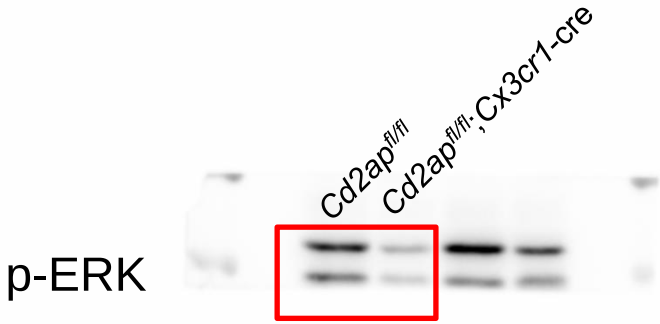

The membrane was imaged with Azure Biosystems 300

Full unedited gel for Supplemental Figure 9F

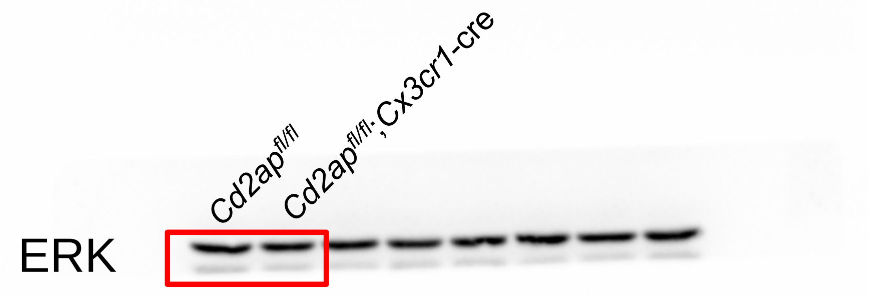

The membrane was imaged with Azure Biosystems 300

Full unedited gel for Supplemental Figure 9F

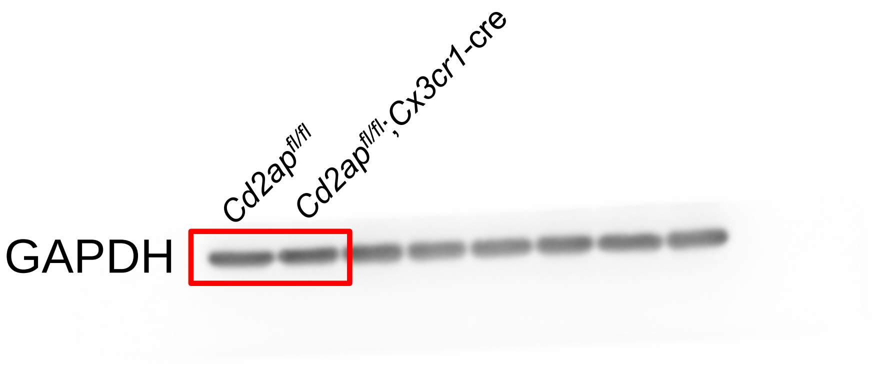

The membrane was imaged with Azure Biosystems 300

Full unedited gel for Supplemental Figure 12A

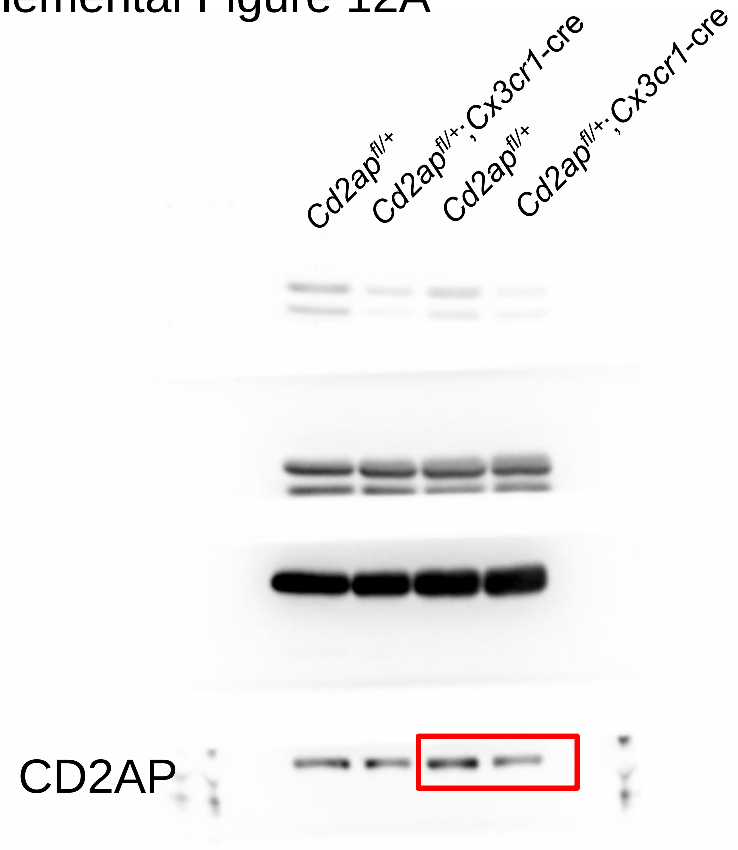

The membrane was imaged with Azure Biosystems 300

Full unedited gel for Supplemental Figure 12A

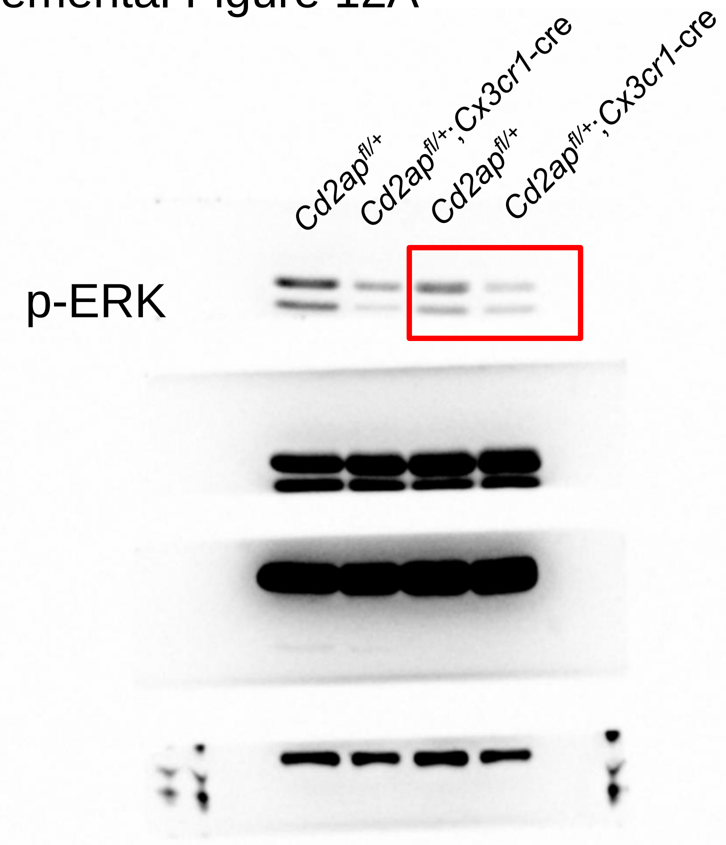

The membrane was imaged with Azure Biosystems 300

Full unedited gel for Supplemental Figure 12A

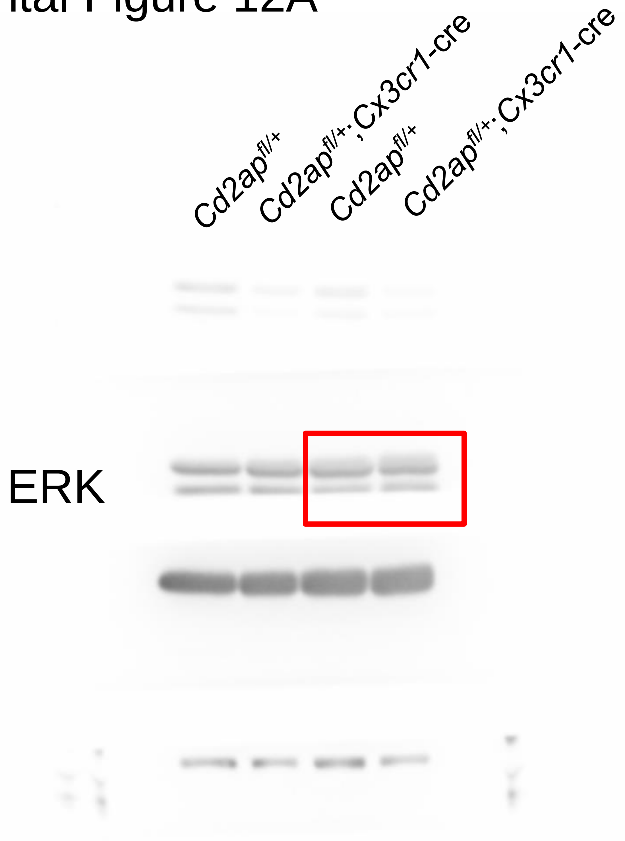

The membrane was imaged with Azure Biosystems 300

Full unedited gel for Supplemental Figure 12A

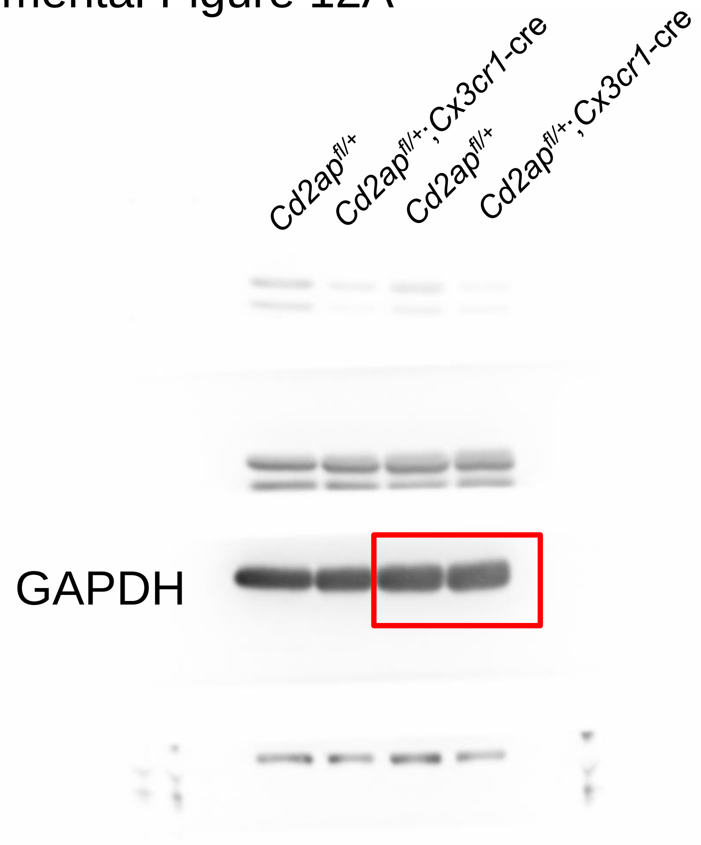

The membrane was imaged with Azure Biosystems 300

Full unedited gel for Supplemental Figure 12C

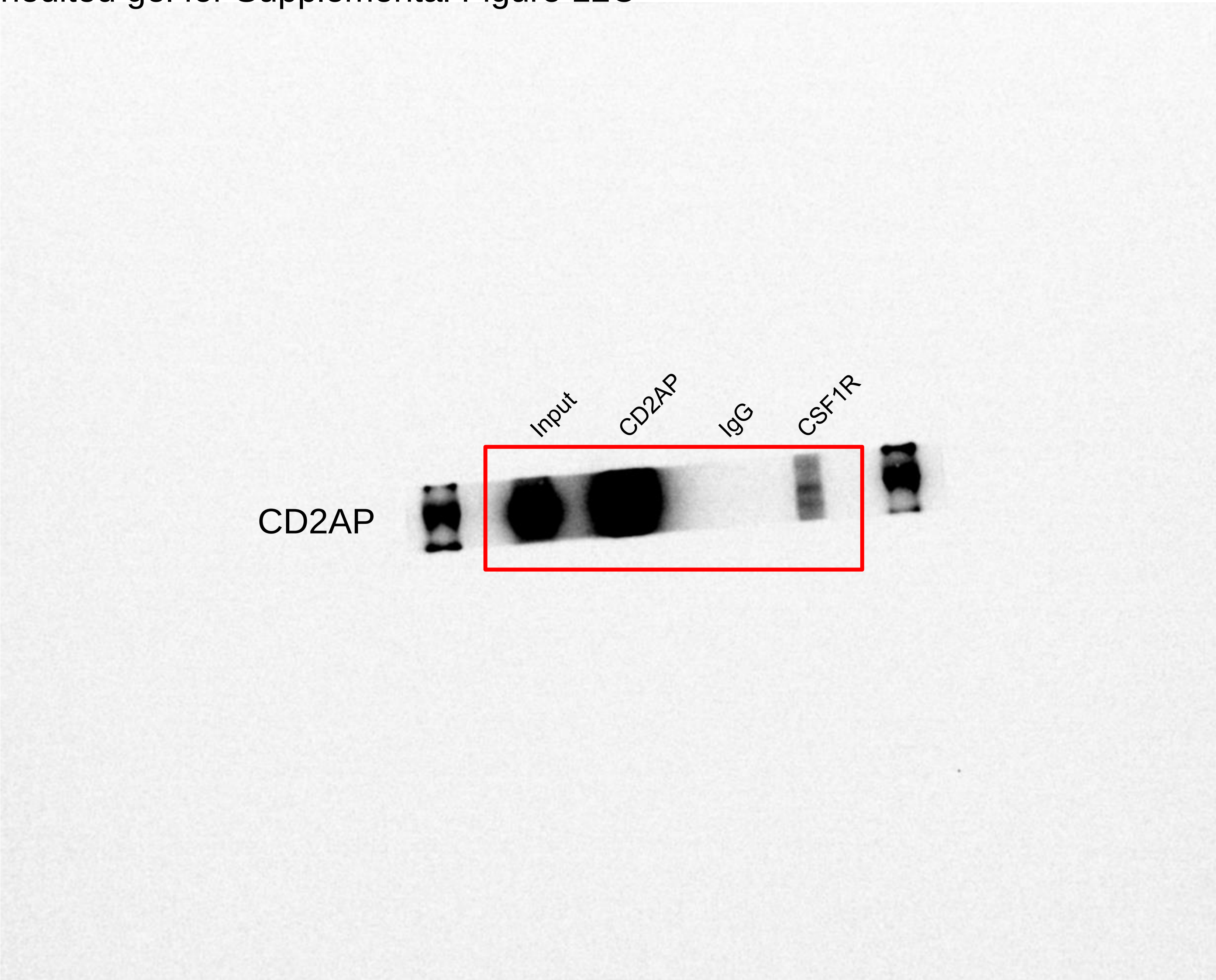

The membrane was imaged with Azure Biosystems 300

Full unedited gel for Supplemental Figure 12C

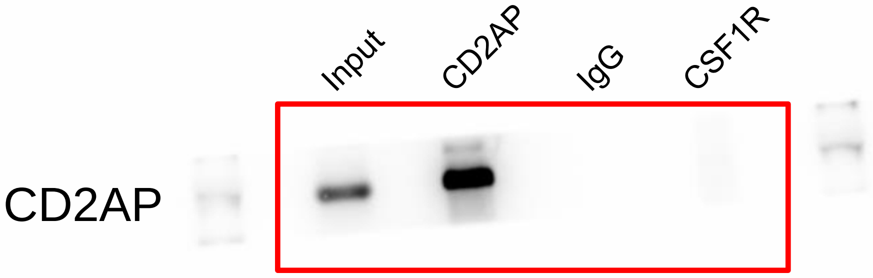

The membrane was imaged with Azure Biosystems 300

Full unedited gel for Supplemental Figure 12D

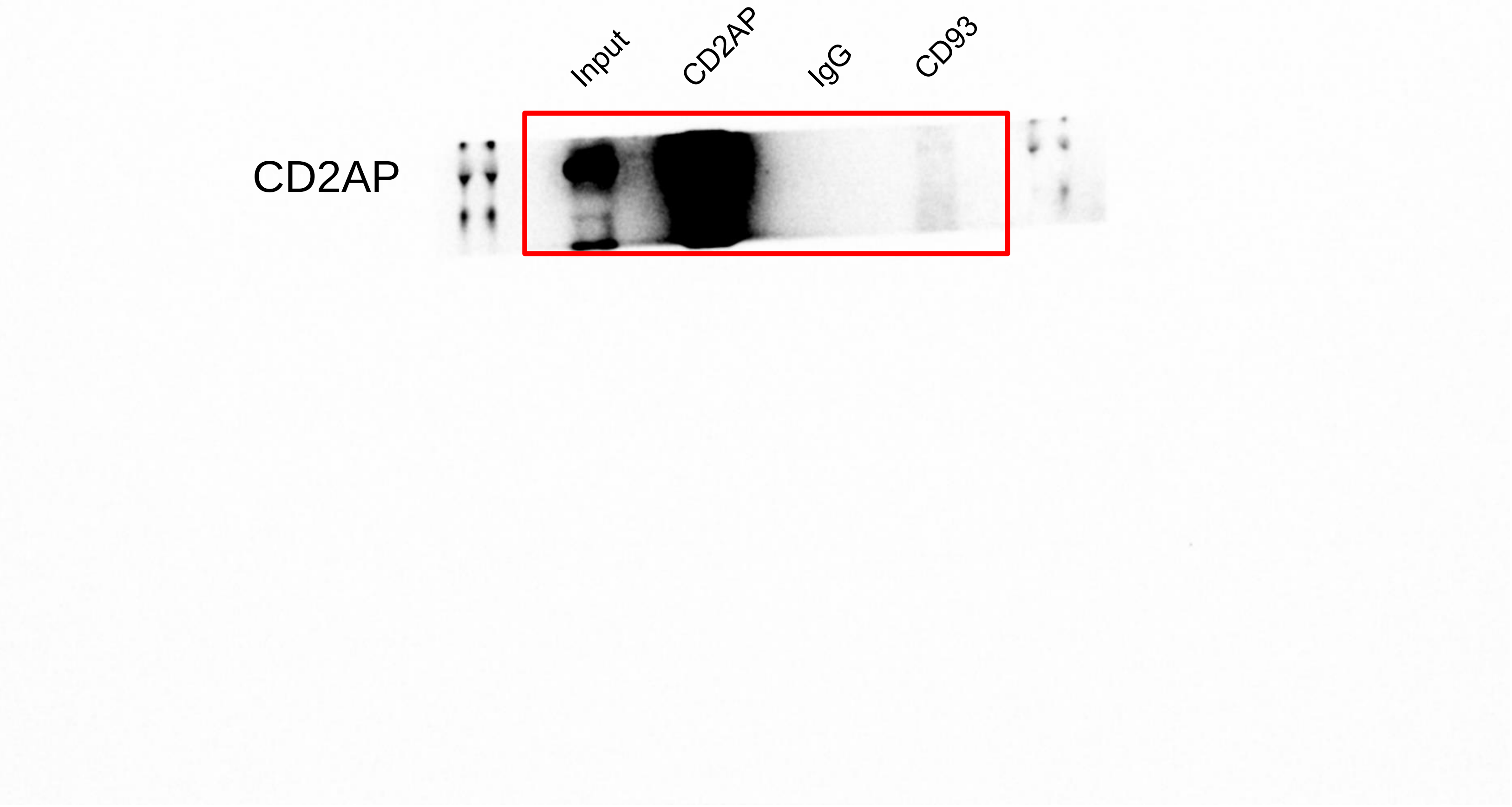

The membrane was imaged with Azure Biosystems 300

Full unedited gel for Supplemental Figure 12D

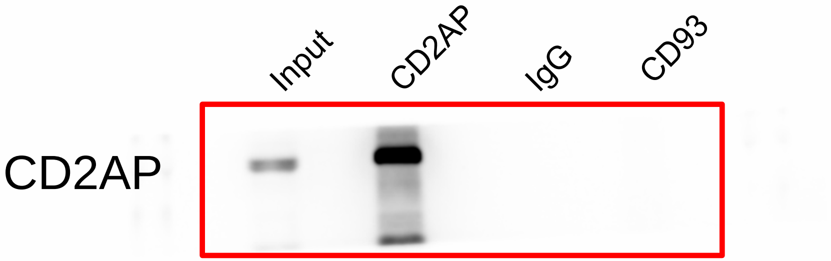

The membrane was imaged with Azure Biosystems 300
